# Connectivity characterization of the mouse basolateral amygdalar complex

**DOI:** 10.1038/s41467-021-22915-5

**Published:** 2021-05-17

**Authors:** Houri Hintiryan, Ian Bowman, David L. Johnson, Laura Korobkova, Muye Zhu, Neda Khanjani, Lin Gou, Lei Gao, Seita Yamashita, Michael S. Bienkowski, Luis Garcia, Nicholas N. Foster, Nora L. Benavidez, Monica Y. Song, Darrick Lo, Kaelan R. Cotter, Marlene Becerra, Sarvia Aquino, Chunru Cao, Ryan P. Cabeen, Jim Stanis, Marina Fayzullina, Sarah A. Ustrell, Tyler Boesen, Amanda J. Tugangui, Zheng-Gang Zhang, Bo Peng, Michael S. Fanselow, Peyman Golshani, Joel D. Hahn, Ian R. Wickersham, Giorgio A. Ascoli, Li I. Zhang, Hong-Wei Dong

**Affiliations:** 1 Stevens Neuroimaging and Informatics Institute, Laboratory of Neuro Imaging, Keck School of Medicine, University of Southern California, Los Angeles, CA USA; 2 Zilkha Neurogenetic Institute, Keck School of Medicine, University of Southern California, Los Angeles, CA USA; 3 Department of Physiology, School of Basic Medical Sciences, Southern Medical University, Guangzhou, China; 4 Brain Research Institute, Department of Psychology, University of California, Los Angeles, CA USA; 5 Department of Neurology, David Geffen School of Medicine, University of California, Los Angeles, CA USA; 6 Semel Institute for Neuroscience and Human Behavior, University of California, Los Angeles, CA USA; 7 West Los Angeles Veterans Administration Medical Center, Los Angeles, CA USA; 8 Department of Biological Sciences, University of Southern California, Los Angeles, CA USA; 9 McGovern Institute for Brain Research, Massachusetts Institute of Technology, Cambridge, MA USA; 10 Krasnow Institute for Advanced Study, George Mason University, Fairfax, VA USA; 11 Center for Neural Circuitry & Sensory Processing Disorders, Zilkha Neurogenetic Institute, Keck School of Medicine, University of Southern California, Los Angeles, CA USA; 12 Present Address: UCLA Brain Research & Artificial Intelligence Nexus, Department of Neurobiology, David Geffen School of Medicine, University of California, Los Angeles, CA USA

**Keywords:** Computational neuroscience, Neural circuits

## Abstract

The basolateral amygdalar complex (BLA) is implicated in behaviors ranging from fear acquisition to addiction. Optogenetic methods have enabled the association of circuit-specific functions to uniquely connected BLA cell types. Thus, a systematic and detailed connectivity profile of BLA projection neurons to inform granular, cell type-specific interrogations is warranted. Here, we apply machine-learning based computational and informatics analysis techniques to the results of circuit-tracing experiments to create a foundational, comprehensive BLA connectivity map. The analyses identify three distinct domains within the anterior BLA (BLAa) that house target-specific projection neurons with distinguishable morphological features. We identify brain-wide targets of projection neurons in the three BLAa domains, as well as in the posterior BLA, ventral BLA, posterior basomedial, and lateral amygdalar nuclei. Inputs to each nucleus also are identified via retrograde tracing. The data suggests that connectionally unique, domain-specific BLAa neurons are associated with distinct behavior networks.

## Abstract (web summary)

The basolateral amygdala is implicated in several behavior-related states including anxiety, autism, and addiction. The authors apply circuit-level pathway tracing methods combined with computational techniques to provide a comprehensive connectivity atlas of the mouse basolateral amygdala complex.

## Introduction

The basolateral amygdalar complex (BLA) contains the lateral (LA), anterior and posterior basolateral (BLAa and BLAp), anterior and posterior basomedial (BMAa and BMAp), as well as the ventral (BLAv) amygdalar nuclei. Although recognized mostly for its role in fear conditioning and extinction^1,2^ the BLA is implicated in several behavior-related states and disorders including anxiety^3^, autism^4^, and addiction^5^. Optogenetic technology has enabled specialized investigations of circuit-specific functional assignments. It is therefore timely to advance understanding of connectivity-specific BLA neurons that will inform higher resolution cell type-specific interrogations.

BLA cell populations with unique connectional-functional phenotypes have been identified. For example, a population of BLA projection neurons that target the prelimbic cortical area (PL) are primarily activated during high fear conditions, while neurons that innervate the infralimbic cortical area (ILA) respond predominantly during fear extinction^6^. These functionally distinct cell groups defined by their projection targets are designated as “fear” and “extinction” neurons, respectively. Similarly, activation of pathways from BLA to the lateral part of the central amygdalar nucleus (BLA→CEAl) and to the anterodorsal part of the bed nuclei of stria terminalis (BLA→BSTad) result in anxiolytic-like behaviors^3,7^, while BLA projections to ventral hippocampus (BLA→vHPC) have been implicated in anxiogenesis^8^. There is the supposition that these functionally specific, uniquely connected neurons are primarily intermingled within BLA^6,9^.

Studies also have demonstrated clear segregation of projection defined BLA cells. Anterior BLA (BLAa) neurons that project to PL^10^ and nucleus accumbens^11^ occupy medial aspects, and those that target the CEA localize in lateral aspects^11^, while ventral hippocampus projecting BLAa neurons are reported in more caudal regions^11,12^. Topographic input to distinct BLAa regions also have been reported^13,14^, suggesting the existence of distinct BLAa domains with unique connections. So where are these target-specific BLA projection neurons located and where would investigators begin to probe cell type function? One approach is to acquire a systematic and detailed overview of BLA connectivity. Although BLA connectivity has been described previously^15^, a systematically acquired whole-brain input/output connectivity profile for BLA neurons has not been created for any species.

In this work, we apply circuit-level pathway tracing methods combined with computational techniques to provide a comprehensive connectivity atlas of the mouse BLA that includes three newly delineated BLAa domains. Online resources for viewing raw, reconstructed, and analyzed data are provided.

## Results

### Pathway tracing, computational analysis, and visualizations

**Fig. 1:**
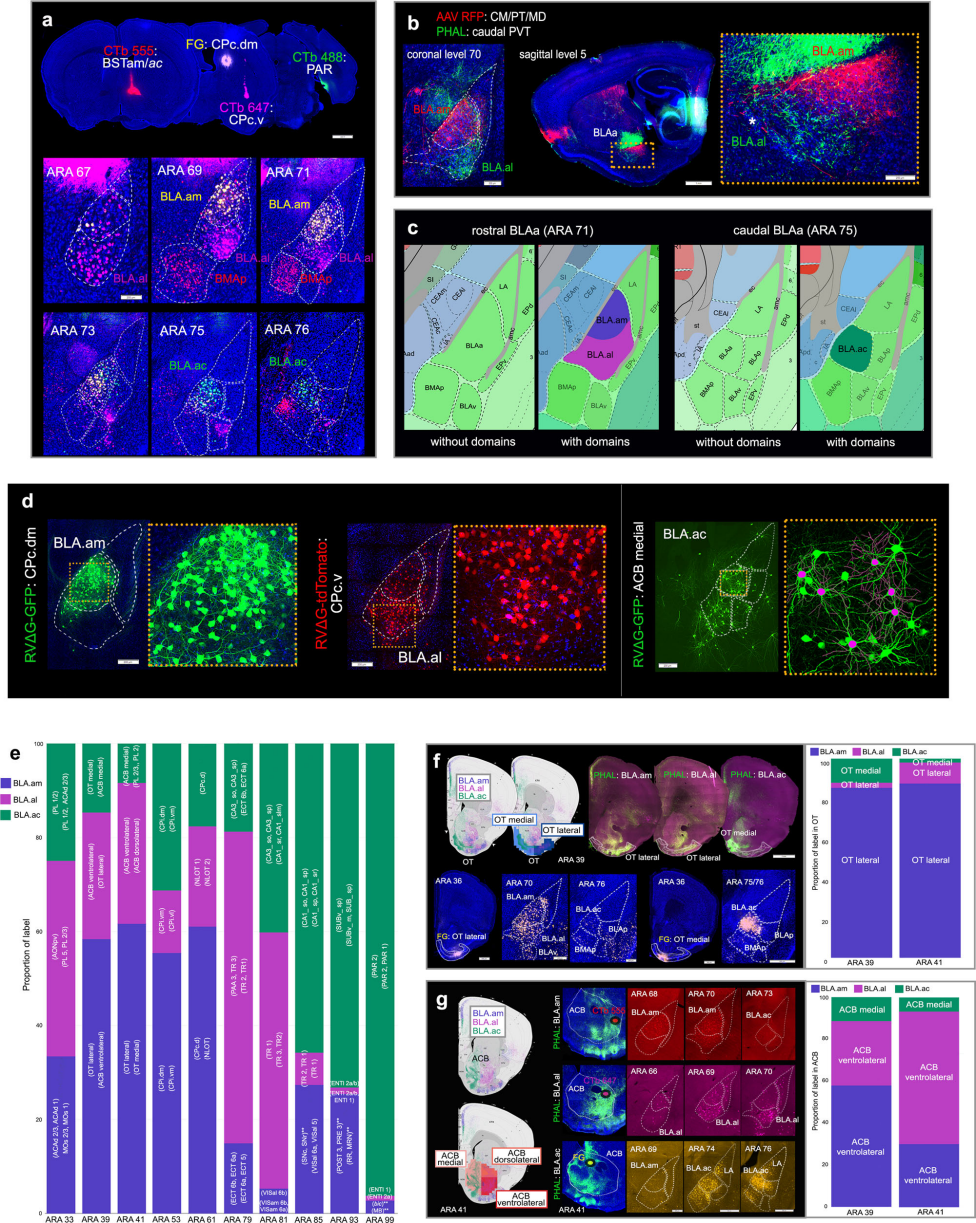
Uniquely connected BLAa neurons. **a** Four retrograde tracer injections in BSTam (CTb 555: red), CPc.dm (FG: yellow), CPc.v (CTb 647: pink), and in PAR (CTb 488: green) reveal uniquely connected projection neurons in BLAa. Note segregation of FG (yellow) and CTb 647 (pink) labeled cells in BLA.am and BLA.al, respectively at ARA levels 69 and 71. Also note absence of PAR projecting CTb 488 (green) labeled cells in at ARA levels 67–71. **b** Anterograde tracers AAV-RFP and PHAL injected in different thalamic nuclei distinctly label BLA.am and BLA.al, which is evident in coronal (left) and sagittal (right) planes. Inset shows magnified version of boxed BLAa region. An asterisk denotes outer boundary of BLAa. **c**. Atlas representations of rostral (ARA 71) and caudal (ARA 75) regions of BLAa with and without domains. **d** Left: RVΔG injected in CPc.dm (green) and CPc.v (red) distinctly label BLA.am and BLA.al projection neurons, respectively. Insets show magnified version of boxed regions. Right: RVΔG injected in ACB medial labels BLA.ac neurons. Soma and dendrites of neurons selected for reconstruction are shown in pink. **e** Bar chart describes proportion of label per ARA section from representative anterograde tracer injections in BLA.am, BLA.al, and BLA.ac (*n* = 1 each) to show their discrete brain-wide connectional patterns. The ROIs for grids with strongest projections from each injection are displayed in parentheses (e.g., strongest projections from BLA.am neurons at ARA 39 are to OT lateral and ACB ventrolateral). Overall, BLA.am has stronger projections at rostral levels compared to BLA.al and BLA.ac. Caudal levels (ARA 85–99) receive more projections from BLA.ac than from BLA.am or BLA.al, primarily targeted to hippocampal structures CA1, SUB, and PAR. Each grid can include more than one ROI [e.g., (PL2, PL1)]. ** denotes anterograde projections that were not validated with retrograde tracers. **f** BLA.am, BLA.al, and BLA.ac projections to OT at ARA 39, which was split into medial (light blue) and lateral (dark blue) regions. BLA.am and BLA.al neurons target OT lateral, while those in BLA.ac target OT medial. Bottom panels show validation of these projections via retrograde FG injections. An OT lateral FG injection strongly labels BLA.am and BLA.al cells, but also BLAv cells, while an OT medial FG injection labels BLA.ac neurons and BLAp and BMAp neurons. The bar chart quantifies and visualizes the density of BLAa→OT connections at ARA levels 39 and 41 (*n* = 1 each). **g** BLA.am, BLA.al, and BLA.ac projections to ACB at ARA 41, which was split into medial (light orange), dorsolateral (orange), and ventrolateral (dark orange) regions. BLA.am and BLA.al neurons target mostly ACB ventrolateral, while those in BLA.ac innervate ACB medial. BLAa→ACB projections are shown with PHAL (green) injections made into BLA.am, BLA.al, and BLA.ac. Ellipses denote locations of retrograde tracer injections used to validate BLAa→ACB connections. CTb 555 injected into ACB ventrolateral regions labels BLA.am neurons (BLA.am→ACB ventrolateral), CTb 647 into ACB ventrolateral regions labels BLA.al neurons (BLA.al→ACB ventrolateral), and FG injected into ACB medial labels BLA.ac neurons (BLA.ac→ACB medial). Bar charts show quantified and visualized density of BLAa→ACB connections at ARA levels 39 and 41 (*n* = 1 each). Abbreviations: ac anterior commissure, ACAd dorsal anterior cingulate area, ACB nucleus accumbens, AONpv anterior olfactory nucleus posteroventral part, bic brachium of the inferior colliculus, BSTam anteromedial bed nucleus of stria terminalis, CA1_so CA1 stratum oriens, CA1_sp CA1 pyramidal layer, CA1_sr CA1 stratum radiatum, CA3_so CA3 stratum oriens, CA3_sp CA3 pyramidal layer, ccg genu of the corpus callosum, CM central medial thalamic nucleus, CPc caudal caudoputamen, CPc.dm caudal caudoputamen, dorsomedial part, CPc.v caudal caudoputamen, ventral part, CPi.dm intermediate caudoputamen, dorsomedial part, CPi.vl intermediate caudoputamen, ventrolateral part, CPi.vm intermediate caudoputamen, ventromedial part, CPc.d caudal caudoputamen, dorsal part, ECT ectorhinal cortical area, ENTl entorhinal cortex, lateral part, MB midbrain, MD mediodorsal thalamic nucleus, MOs secondary motor area, MRN midbrain reticular nucleus, NLOT nucleus of the lateral olfactory tract, OT olfactory tubercle, PAR parasubiculum, PL prelimbic cortical area, POST postsubiculum, PRE presubiculum, PT parataenial thalamic nucleus, PVT paraventricular thalamic nucleus, RR retrorubral area, SNc substantia nigra, compact part, SNr substantia nigra reticular part, SUBv_m ventral subiculum molecular layer, SUBv_sp ventral subiculum pyramidal layer, TR postpiriform transition area, VISal anterolateral visual area, VISam anteromedial visual area. See Table 1 for full list of abbreviations.

Connectivity data was collected at USC as part of the Mouse Connectome Project (MCP), currently at UCLA. Anterograde (PHAL, BDA, AAV) and retrograde (CTb, FG, AAV retro Cre) pathway tracers were placed in BLAa, BLAp, BLAv, BMAp, and LA (cases can be viewed at https://mouseconnectomeproject.github.io/amygdalar/iconnectome). Tracing data from injections targeting medial prefrontal cortex (MPF) and hippocampus (HPF) clearly delineated three BLAa domains: medial (BLA.am), lateral (BLA.al), and caudal (BLA.ac). Additional injections in cortex, thalamus, and striatum validated these domain distinctions (Fig. 1a–c; Supplementary Fig. 1a–c).

**Fig. 2:**
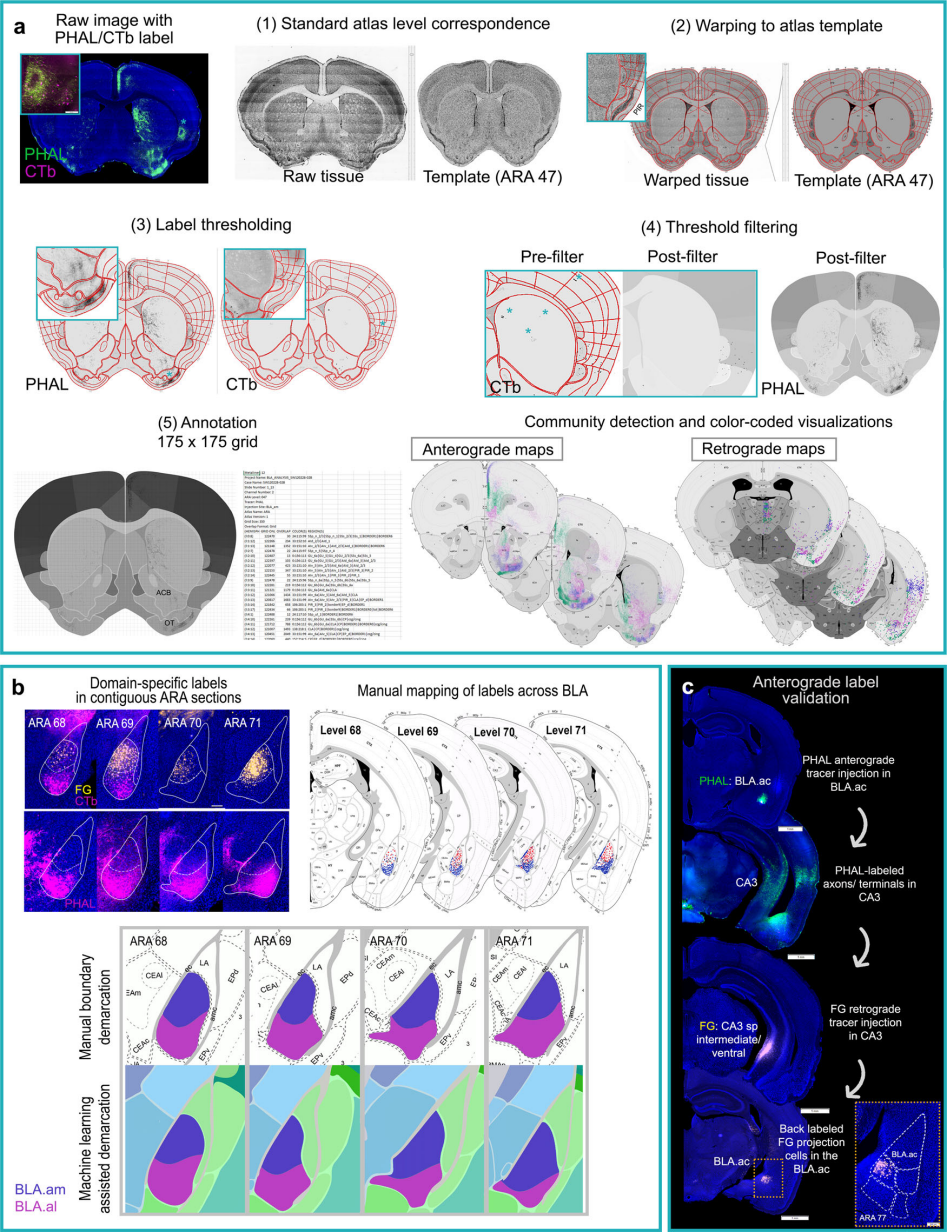
Data processing and analysis workflows. **a** Summary of our 2D post-image processing pipeline. Images with fluorescent tracers (e.g., green PHAL fibers and pink CTb cells) are acquired under 10x magnification. Images are then imported into an in house software Connection Lens for (1) atlas correspondence to match each section to its corresponding ARA atlas template, (2) to warp data sections to atlas templates, (3) to threshold labeling, (4) filter artifacts, and (5) annotate the labels. Boxed region in (2) shows magnification of piriform cortex (PIR) to illustrate accuracy of registration details. Insets in (3) display magnified regions demonstrating accuracy of segmented PHAL fibers and CTb cells. In (4) asterisks highlight filtered artifacts to reduce false positive signals. Annotation was performed at the grid level (175 × 175 pixels) to capture topographic labels within ROIs that otherwise would go undetected [e.g., olfactory tubercle (OT), nucleus accumbens (ACB)]. Overlap processing (5) results in an file with annotated values: pixel density for anterograde tracers and cell counts for retrograde tracers. A modularity maximization algorithm is applied to the annotated data to assign labels to an injection site based on label density. Community assignments are color-coded by injection site and visualized for 32 ARA sections for anterograde and retrograde maps (available at https://mouseconnectomeproject.github.io/amygdalar/). **b** Workflow for delineating BLAa domain boundaries (also Supplementary Fig. 2). First, cases with domain-distinct labels were selected (*n* = 7) and contiguous sections through the BLAa were collected, imaged, and registered. Labels for each case were manually mapped onto a standard atlas in individual layers. Borders were manually drawn guided by mapped labels, but also by Nissl cytoarchitecture (Supplementary Fig. 3b). The same data was used to train a machine learning algorithm, which produced similar automated delineations of BLAa domains (Supplementary Fig. 3a). **c** Validation of anterograde labeling with retrograde tracers. PHAL injected in BLA.ac labels CA3. A FG injection in CA3_sp pyramidal layer back-labels BLA.ac projection cells confirming the BLA.ac→CA3 connection. Inset is magnification of boxed region showing selective and confined CA3 projecting FG-labeled cells in BLA.ac.

To determine BLAa domain boundaries, representative cases with distinct labeling in each domain were selected and all consecutive sections across the BLA were analyzed (Supplementary Fig. 2). Sections underwent image processing through our in-house software Connection Lens, where each section was matched and warped to its corresponding atlas level of the Allen Reference Atlas (ARA) and the labeling was segmented (Fig. 2a). Labels were manually mapped and aggregated atop the ARA, and approximate boundaries were delineated for BLA.am, BLA.al, and BLA.ac (Fig. 2b; Supplementary Fig. 3a, b). The same data was used as a training set for a machine learning algorithm to compute BLAa domain boundaries^16–18^. The automated boundary demarcations highly corroborated those of the manual delineations, returning an average agreement of 92% (Fig. 2b; Supplementary Fig. 3a). Utilizing these boundary approximations, anterograde and retrograde tracer injections were made to involve primarily BLA.am, BLA.al, or BLA.ac (Supplementary Fig. 4a) as well as BLAp, BLAv, LA (ventromedial region), or BMAp (Supplementary Fig. 4c).

**Fig. 3:**
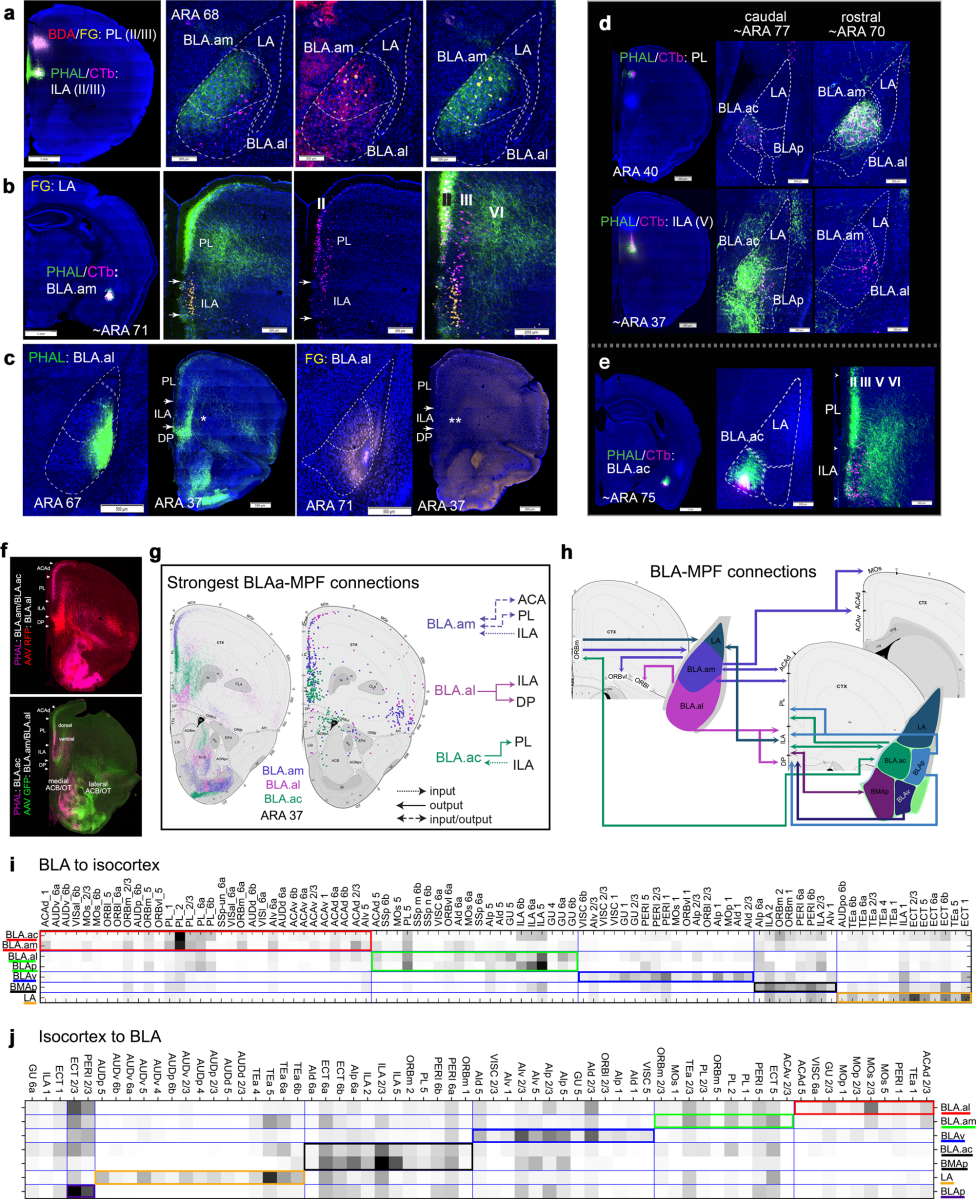
BLAa connections with medial prefrontal cortex. **a** Double co-injections of BDA/FG in PL(II/III) and PHAL/CTb in ILA(II/III) show the medial (BLA.am) and lateral (BLA.al) distinction of BLAa. PHAL and BDA fibers and FG cells are present in BLA.am, while CTb cells are in BLA.al suggesting PL→BLA.am, ILA→BLA.am, and BLA.al→ILA connections. **b** These connections were validated with a BLA.am PHAL/CTb injection, which shows strong fiber labeling in PL, especially layer II/III [BLA.am→PL(II/III)] and CTb labeling in PL(II) [PL(II)→BLA.am] and ILA(III) [ILA(III)→BLA.am]. The LA FG injection delineates the ILA. **c** The BLA.al→ILA connection was validated with a BLA.al PHAL injection, which showed strong fiber labels in ILA and DP. FG injection in BLA.al confirms the absence of an ILA→BLA.al connection. **denotes lack of FG ILA labeling. **d** BLA.ac shows unique connections with MPF. A PHAL/CTb injection in PL shows CTb labeled BLA.ac projection cells, but sparse PHAL fiber labels in BLA.ac, suggesting a BLA.ac→PL connection. PHAL fibers from PL localize mostly in rostral BLA.am. A PHAL/CTb injection in ILA(V) shows strong fiber projections in BLA.ac suggesting a strong ILA→BLA.ac connection. Only a few CTb cells are present in BLA.ac suggesting a weak BLA.ac→ILA connection. **e** A BLA.ac PHAL/CTb injection validates these connections, showing strong projections to PL, especially layer II/III [BLA.ac→PL(II/III)] and CTb labeled cells in layers II–V of ILA (ILA→BLA.ac). **f** Top panel: anterograde labeling from tracer injections made primarily in BLA.am (PHAL) and BLA.al (AAV RFP) shows their topographic projections to PL/ACA and ILA/DP, respectively. Bottom panel: anterograde labeling from tracer injections made primarily in BLA.ac (PHAL) and BLA.al (AAV GFP) shows their distinct connections with PL, ILA, ACB, OT, and CP. Note the stronger projections from BLA.am to dorsal PL/ACA compared to projections from BLA.ac to more ventral parts of PL. This distinction can be seen in the anterograde map in (**g**). **g** BLAa domains share unique input/output connections with MPF, especially layers II/III, which is summarized in this schematic. Note (1) the reciprocal connections between the BLA.am and ACA and PL (dorsal), (2) BLA.al projections mostly to ILA, and (3) the unique BLA.ac connections with strong projections to PL (ventral), but strong input from ILA. **h** Schematic summarizing connections of all BLA nuclei with MPF areas. **i** Community detection confined to BLA projections to isocortical areas was run and visualized in a matrix. The matrix was reordered such that injection sites grouped with their strongest projections are arranged along the diagonal. Grouped injection sites and their connections are boxed in different colors. The weighting of each connection is indicated by a color gradient from black (very strong) to white (very weak). Matrix analysis was ROI based, not grid-based. BLA.am and BLA.ac were grouped with projections to PL(I–III,VI), ORBm, and ACAd among the strongest. The BLA.al and BLAp were grouped with strongest projections to ILA, GU, and PL(V). The BLAv shows strongest projections to AI, GU, and PERI, the BMAp to ILA, ORBm, PERI, and the LA to ECT, TEa, and ILA. **j** Community detection confined to isocortical projections to BLA was run and visualized in a matrix, which shows BLA.ac and BMAp grouped with strongest input from ILA(II/III). The BLA.al, BLA.am, BLAv, LA, and BLAp were individually grouped. Note the strong inputs to BLAv from agranular insular areas (AI) and to LA from auditory cortices (AUD). See Table 1 for full list of abbreviations.

**Fig. 4:**
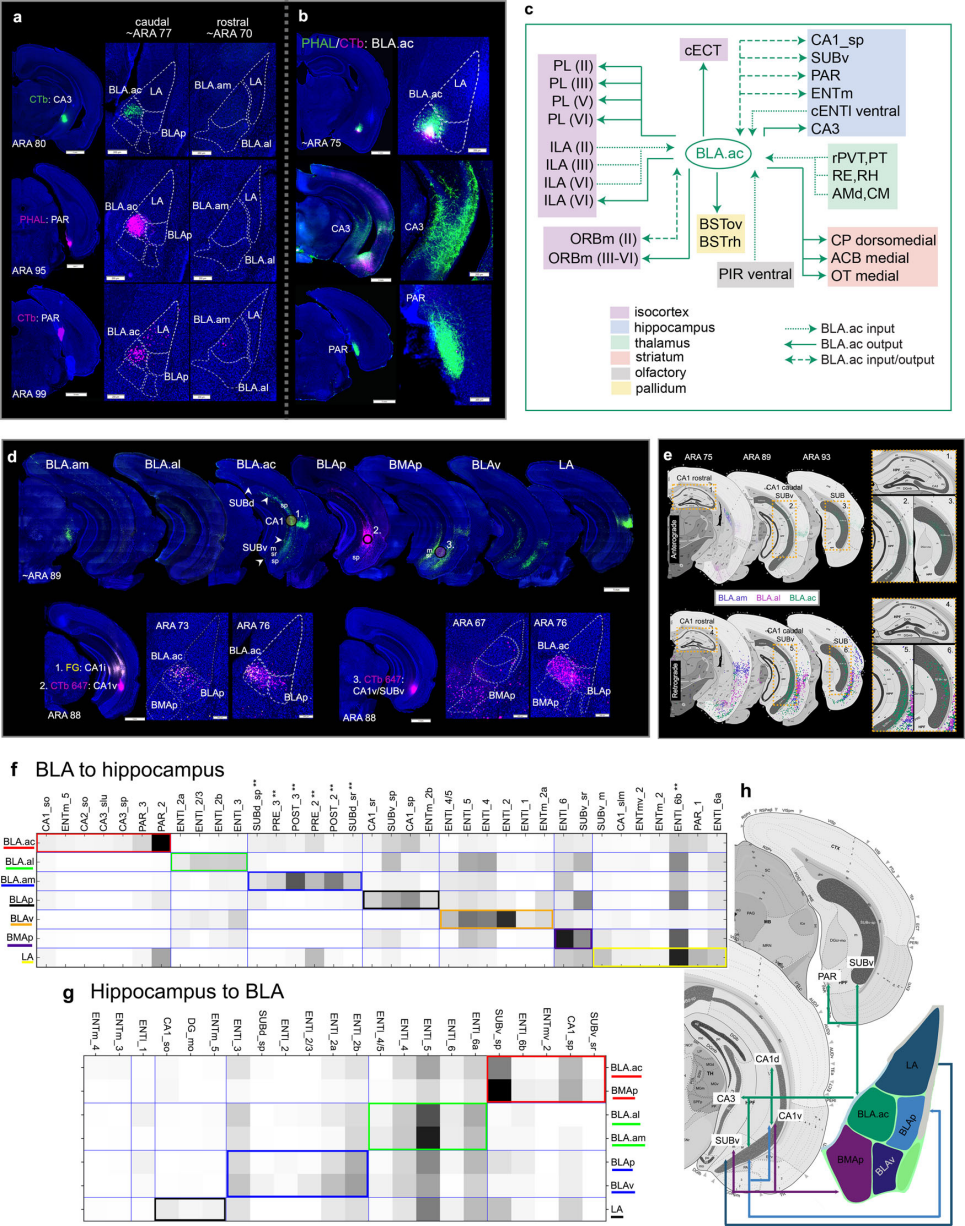
BLA.ac connections with hippocampus. **a** BLA.ac connections with hippocampal regions. A CTb (green) CA3 tracer injection selectively labels BLA.ac projection neurons (BLA.ac→CA3). Note the absence of labeled cells in BLA.am. A PHAL (pink) and CTb (pink) injection in PAR show the BLA.ac→PAR and PAR→BLA.ac connections. **b** These connections were validated with a PHAL/CTb BLA.ac injection that shows strong PHAL fiber labels in CA3 and PAR. **c** Summarized brain-wide connections of BLA.ac projection neurons. For full abbreviation list see Table 1. **d** Top panels show projections from BLA.am, BLA.al, BLA.ac, BLAp, BMAp, BLAv, and LA neurons to hippocampal regions. The BLA.ac, BLAp, and BMAp show strongest projections, with BLA.ac projecting to sp layers of CA1 and SUBv (BLA.ac→CA1_sp/SUBv), BLAp to CA1v and SUBv (BLAp→CA1v_sp/SUBv), and BMAp to sr and m layers of CA1v and SUBv (BMAp→CA1v_sr/SUBv_m/sr). Bottom panels show validation of these connections with FG and CTb injections marked 1–3 on top panels. Injections 1 and 2 show FG and CTb injections in intermediate (CA1i) and ventral (CA1v) CA1, respectively. Both injections back-label projection neurons in BLA.ac, while only the injection in CA1v labels BLAp neurons. Injection 3 is a CTb injection in SUBv, which labels BLA.ac, BLAp, and BMAp neurons. **e** Top panels show BLAa projections to rostral CA1 and more caudal CA1 and SUB. Boxed regions are numbered and magnified to the right. Bottom panels show projections from CA1 and SUB back to BLAa. Note exclusive BLA.ac connections with the hippocampus, particularly its caudal regions (Fig. 7n shows PHAL validation of these connections). **f** Community detection confined to BLA projections to hippocampal areas was run and visualized in a matrix. The matrix was reordered such that grouped injection sites and their strongest projections are arranged along the diagonal and boxed in different colors. The weighting of each connection is indicated by a color gradient from black (very strong) to white (very weak). Note (1) projections from BLA.ac to PAR and CA3, (2) from BLAp to CA1_sp and SUBv_sp, (3) from BLAv to ENTl (layers II and V), and (4) from BMAp to SUBv_sr. ** indicates strong connections that were not validated. **g** Community detection confined to hippocampal inputs to BLA was run and visualized in a matrix. Note (1) BLA.ac and BMAp are grouped with strong inputs from CA1 and SUBv and (2) BLA.am and BLA.al grouped with strong input from ENTl. Matrix analysis was ROI based, not grid based. **h** Schematic summarizing connections of all BLA nuclei with hippocampal areas. See Table 1 for full list of abbreviations.

To attain brain-wide connectivity patterns of each BLA nucleus, all data was processed through Connection Lens (Fig. 2a). Data was annotated at a 175 × 175 pixel grid resolution given the complexity of topographic projections from BLA to ROIs like the nucleus accumbens or olfactory tubercle (Fig. 1f, g). Subsequent Louvain community detection analysis assigned each injection site to a community based on labeling patterns of the individual grid cells. Community assignments were color-coded by injection site and visualized for 32 ARA sections to create anterograde and retrograde connectivity maps (Fig. 2a), which are available through a web application (https://mouseconnectomeproject.github.io/amygdalar/) (Supplementary Fig. 1d). Community analysis was isolated across separate categories of injections to identify regions of unique input and output. Data from BLA.am, BLA.al, and BLA.ac were aggregated, analyzed, and visualized, while separate analyses were conducted to compare BLAp to BLA.al, and BLAv, BMAp, LA. In addition, data for all BLA nuclei were combined and subjected to community detection analysis with resulting outputs visualized in a matrix (Fig. 3i, j; Fig. 4f, g).

**Table 1:** Table of brain structure abbreviations.

Acronym	Full structure name
AAA	Anterior amygdalar area
*ac*	Anterior commissure
ACAd	Anterior cingulate cortical area, dorsal part
cACAd	Anterior cingulate cortical area, dorsal part, caudal region
rACAd	Anterior cingulate cortical area, dorsal part, rostral region
ACAv	Anterior cingulate cortical area, ventral part
cACAv	Anterior cingulate cortical area, ventral part, caudal region
rACAv	Anterior cingulate cortical area, ventral part, rostral region
ACB	Nucleus accumbens
AD	Anterodorsal nucleus of the thalamus
ADP	Anterodorsal preoptic nucleus of the hypothalamus
AHN	Anterior hypothalamic nucleus
AI	Agranular insular cortical area
AId	Agranular insular cortical area, dorsal part
AIp	Agranular insular cortical area, posterior part
AIv	Agranular insular cortical area, ventral part
AM	Anteromedial nucleus of the thalamus
AMd	Anteromedial nucleus of the thalamus, dorsal part
AMv	Anteromedial nucleus of the thalamus, ventral part
AON	Anterior olfactory nucleus
AONm	Anterior olfactory nucleus, medial part
AONpv	Anterior olfactory nucleus, posteroventral part
ARH	Arcuate hypothalamic nucleus
AUD	Auditory cortical area
AUDd	Dorsal auditory cortical area
AUDp	Primary auditory cortical area
AUDv	Ventral auditory cortical area
AV	Anteroventral nucleus of the thalamus
AVP	Anteroventral preoptic nucleus
AVPV	Anteroventral periventricular nucleus
*bic*	Brachium of the inferior colliculus
BLA	Basolateral amygdalar complex
BLAa	Basolateral amygdalar nucleus, anterior part
BLA.ac	Basolateral amydalar nucleus, anterior part, caudal domain
BLA.al	Basolateral amydalar nucleus, anterior part, lateral domain
BLA.am	Basolateral amydalar nucleus, anterior part, medial domain
BLAp	Basolateral amygdalar nucleus, posterior part
BLAv	Basolateral amygdalar nucleus, ventral part
BMA	Basomedial amygdalar nucleus
BMAa	Basomedial amygdalar nucleus, anterior part
BMAp	Basomedial amygdalar nucleus, posterior part
BST	Bed nucleus of the stria terminalis
BSTal	Bed nucleus of the stria terminalis, anterolateral nucleus
BSTam	Bed nucleus of the stria terminalis, anteromedial nucleus
BSTdm	Bed nucleus of the stria terminalis, dorsomedial nucleus
BSTif	Bed nucleus of the stria terminalis, interfascicular nucleus
BSTmg	Bed nucleus of the stria terminalis, magnocellular nucleus
BSTpr	Bed nucleus of the stria terminalis, principal nucleus
BSTtr	Bed nucleus of the stria terminalis, transverse nucleus
BSTv	Bed nucleus of the stria terminalis, ventral nucleus
CA1	Hippocampal field CA1
CA1d	CA1 dorsal
CA1_slm	CA1 stratum lacunosum-moleculare
CA1_so	CA1 stratum oriens
CA1_sp	CA1 pyramidal layer
CA1_sr	CA1 stratum radiatum
CA1v	CA1 ventral
CA2	Hippocampal field CA2
CA2_so	CA2 stratum oriens
CA3	Hippocampal field CA3
CA3_so	CA3 stratum oriens
CA3_sp	CA3 pyramidal layer
CEA	Central amygdalar nucleus
CLA	Claustrum
CM	Central medial nucleus of the thalamus
COA	Cortical amygdalar area
COAa	Cortical amygdalar area, anterior part
COApl	Cortical amygdalar area, posterior part, lateral zone
COApm	Cortical amygdalar area, posterior part, medial zone
CP	Caudoputamen
CPc	Caudoputamen, caudal part
CPc.dm	Caudoputamen, caudal part, dorsomedial domain
CPc.v	Caudoputamen, caudal part, ventral domain
CPi	Caudoputamen, intermediate part
CPi.dm	Caudoputamen, intermediate part, dorsomedial domain
CPi.v	Caudoputamen, intermediate part, ventral domain
CPi.vl	Caudoputamen, intermediate part, ventrolateral domain
CPi.vm	Caudoputamen, intermediate part, ventromedial domain
DMH	Dorsomedial nucleus of the hypothalamus
DP	Dorsal peduncular area
ECT	Ectorhinal cortical area
cECT	Ectorhinal cortical area, caudal region
rECT	Ectorhinal cortical area, rostral region
ENT	Entorhinal cortical area
ENTl	Entorhinal cortical area, lateral part
cENTl	Entorhinal cortical area, lateral part, caudal region
rENTl	Entorhinal cortical area, lateral part, rostral region
ENTm	Entorhinal cortical area, medial part
EP	Endopiriform nucleus
EPd	Endopiriform nucleus, dorsal part
EPv	Endopiriform nucleus, ventral part
*fr*	Fasciculus retroflexus
FS	Fundus of the striatum
GU	Gustatory cortical area
IAD	Interanterodorsal nucleus of the thalamus
ILA	Infralimbic cortical area
IMD	Intermediodorsal nucleus of the thalamus
*int*	Internal capsule
LA	Lateral amygdalar area
LD	Laterodorsal nucleus of the thalamus
LHA	Lateral hypothalamic area
LP	Lateral posterior nucleus of the thalamus
LPO	Lateral preoptic nucleus
LS	Lateral septal nucleus
LSc	Lateral septal nucleus, caudal part
LSr	Lateral septal nucleus, rostral part
LSv	Lateral septal nucleus, ventral part
MB	Midbrain
MD	Mediodorsal nucleus of the thalamus
MDm	Mediodorsal nucleus of the thalamus, medial part
MEA	Medial amygdalar nucleus
MEAad	Medial amygdalar nucleus, anterodorsal part
MEAav	Medial amygdalar nucleus, anteroventral part
MEApd	Medial amygdalar nucleus, posterodorsal part
MEApv	Medial amygdalar nucleus, posteroventral part
MEPO	Median preoptic nucleus
MG	Medial geniculate complex
MGd	Medial geniculate complex, dorsal part
MGm	Medial geniculate complex, medial part
MGv	Medial geniculate complex, ventral part
MM	Medial mammillary nucleus
MOp	Primary motor cortical area
MOp m	Primary motor cortical area, mouth region
MOp ul	Primary motor cortical area, upper limb region
rMOp	Primary motor cortical area, rostral region
MOs	Secondary motor cortical area
MOs fef	Secondary motor cortical area, frontal eye field region
MOs ul	Secondary motor cortical area, upper limb region
rMOs	Secondary motor cortical area, rostral region
MPN	Medial preoptic nucleus
MPO	Medial preoptic area
MRN	Midbrain reticular nucleus
MS	Medial septal nuclei
*mtt*	Mammillothalamic tract
NDB	Nucleus of the diagonal band
NLOT	Nucleus of the lateral olfactory tract
ORB	Orbital cortical area
ORBl	Orbital cortical area, lateral part
ORBm	Orbital cortical area, medial part
ORBvl	Orbital cortical area, ventrolateral part
OT	Olfactory tubercle
PA	Posterior amygdalar nucleus
PAA	Piriform amygdalar area
PAR	Parasubiculum
PERI	Perirhinal cortical area
cPERI	Perirhinal cortical area, caudal region
rPERI	Perirhinal cortical area, rostral region
PF	Parafascicular nucleus of the thalamus
PH	Posterior hypothalamic nucleus
PIR	Piriform cortical area
PL	Prelimbic cortical area
PMv	Ventral premammillary nucleus
POST	Postsubiculum
PP	Peripeduncular nucleus
PRE	Presubiculum
PT	Parataenial nucleus of the thalamus
PTLp	Posterior parietal association area
PVp	Periventricular hypothalamic nucleus, posterior part
PVT	Paraventricular nucleus of the thalamus
cPVT	Paraventricular nucleus of the thalamus, caudal region
rPVT	Paraventricular nucleus of the thalamus, rostral region
RCH	Retrochiasmatic area
RE	Reuniens nucleus of the thalamus
RH	Rhomboid nucleus of the thalamus
RR	Retrorubral area
RSP	Retrosplenial cortical area
RSPagl	Retrosplenial cortical area, agranular part
RSPd	Retrosplenial cortical area, dorsal part
RSPv	Retrosplenial cortical area, ventral part
RT	Reticular nucleus of the thalamus
SC	Superior colliculus
SI	Substantia innominata
*sm*	Stria medullaris
SN	Substantia nigra
SNc	Substantia nigra, compact part
SNr	Substantia nigra reticular part
SPFp	Subfascicular nucleus, parvocellular part
SSp	Primary somatosensory cortical area
SSp m	Primary somatosensory cortical area, mouth region
SSs	Supplementary somatosensory cortical area
SUB	Subiculum
SUBd	Subiculum, dorsal part
SUBv	Subiculum, ventral part
SUBv_m	Subiculum, ventral part, molecular layer
SUBv_sp	Subiculum, ventral part, pyramidal layer
SUBv_sr	Subiculum, ventral part, stratum radiatum
TEa	Temporal association cortical area
cTEa	Temporal association cortical area, caudal region
rTEa	Temporal association cortical area, rostral region
TM	Tuberomammillary nucleus
TMd	Tuberomammillary nucleus, dorsal part
TMv	Tuberomammillary nucleus, ventral part
TR	Postpiriform transition area
TT	Taenia tecta
TTd	Taenia tecta, dorsal part
TTv	Taenia tecta, ventral part
TU	Tuberal nucleus
VIS	Visual cortical area
VISal	Anterolateral visual cortical area
VISam	Anteromedial visual cortical area
VISl	Lateral visual cortical area
VISp	Primary visual cortical area
VISC	Visceral cortical area
VLPO	Ventrolateral preoptic nucleus
VMH	Ventromedial hypothalamic nucleus
VMHd	Ventromedial hypothalamic nucleus, dorsal part
VP	Ventral pallidum
VPMpc	Ventral posteromedial nucleus of the thalamus, parvocellular part

Lower case, italicized acronyms denote fiber tracts.

Reported connections were validated using at least one of the following methods. Injections for all BLA nuclei were replicated and consistency across label patterns was manually assessed (Supplementary Fig. 4b). Retrograde tracers were injected into regions of anterograde terminal labeling to validate anterograde connections, but also to reveal the location of target-specific projection neurons (Fig. 2c). Anterograde tracers were injected into the location of back-labeled projection cells to validate retrograde BLA injection data (Supplementary Fig 4d, e). Quantitative comparisons of projection labels from BLAa domains were performed to supplement and validate the qualitative analysis (Supplementary Fig. 5b–m). Hierarchical clustering of anterograde projection data showed that repeated injections in BLAa domains produced highly consistent domain-specific label patterns, substantiating the validity and reproducibility of injection cases (Supplementary Fig. 6a). A total of 245 injections (44 in BLA) were used to generate nucleus-unique connectivity diagrams and a global wiring diagram of the entire BLA (https://mouseconnectomeproject.github.io/amygdalar/wiringdiagram) (Supplementary Fig. 6b).

### Parcellation of BLAa connectivity-defined cell types projecting to medial prefrontal cortex (MPF) and hippocampal formation (HPF)

BLAa shares extensive bidirectional connectivity with the ILA and PL in MPF^14,19,20^. These connections were validated and it was demonstrated that input to each MPF area originates from two regionally distinct BLAa neuron populations. An anterograde and retrograde tracer co-injection (BDA/FG) in PL labels overlapping fibers and projection cells in medial parts of BLAa (BLA.am), suggesting strong BLA.am→PL and PL→BLA.am connections (Fig. 3a). A co-injection (PHAL/CTb) in ILA shows axon terminals in BLA.am with retrogradely labeled projection neurons located mostly in lateral BLAa (BLA.al) (BLA.al→ILA) (Fig. 3a), suggesting a BLA.al→ILA→BLA.am circuit.

A PHAL/CTb co-injection in BLA.am confirms the presence of this circuit and reveals the detailed regional and laminar specificities of BLA.am-MPF connections. BLA.am neurons densely innervate PL layer II, but also layers V and VI [BLA.am→PL(II,V,VI)] (Fig. 3b; Supplementary Fig. 7a). ILA remains relatively void of inputs, corroborating sparse BLA.am→ILA connectivity. Further, BLA.am projecting PL neurons distribute primarily in layers II/III, and in layer III of ILA, confirming and refining the ILA(III)→BLA.am↔PL(II) pathway. Finally, PHAL and FG injections in BLA.al validate the unidirectional BLA.al→ILA connection and reveal laminar details [BLA.al→ILA(II-VI)] (Fig. 3c).

Neurons in the caudal BLAa (BLA.ac) share unique connections with MPF that differ from its BLAa counterparts. Strongest BLA.ac projections to MPF are to PL, primarily layer II (Fig. 3e), but also layers III-VI [BLA.ac→PL(II-VI)], which lightly project back to BLA.ac (Fig. 3d, e). Most PL projections to BLAa are to BLA.am (PL→BLA.am), while strongest ILA projections are to BLA.ac [ILA(II/III)→BLA.ac] (Fig. 3d). BLA.ac neurons also target ILA [BLA.ac→ILA(II-VI)] (Fig. 3e). Multiple injections of anterograde tracers in BLA.am, BLA.al, and BLA.ac made in a single animal highlight their distinct input to MPF structures (Fig. 3f). These unique BLAa-MPF connections are summarized in Fig. 3g, h.

BLA.ac neurons are the only BLAa cells to target HPF, and their connections with CA3 and parasubiculum (PAR) are primarily exclusive among all BLA nuclei. Retrograde tracer injections in CA3 and PAR selectively label BLA.ac projection neurons (BLA.ac→CA3; BLA.ac→PAR) (Fig. 4a, b). In addition, BLA.ac neurons target the pyramidal layers of CA1 (BLA.ac→CA1_sp) and ventral subiculum (BLA.ac→SUBv) (Fig. 4d, e). Besides CA3, all these HPF regions send inputs back to BLA.ac (CA1_sp/SUBv/PAR→BLA.ac) (Fig. 4e). See Fig. 4h for summarized connections of BLA and HPF and Fig. 4f, g for BLA-hippocampal connectivity matrices.

### Global connections of the BLA.am, BLA.al, and BLA.ac

An overview of BLA.am, BLA.al, and BLA.ac projection neuron targets demonstrates their discrete brain-wide connectivity patterns (Fig. 1e; Supplementary Fig. 5a; Supplementary Fig. 8).

**Fig. 5:**
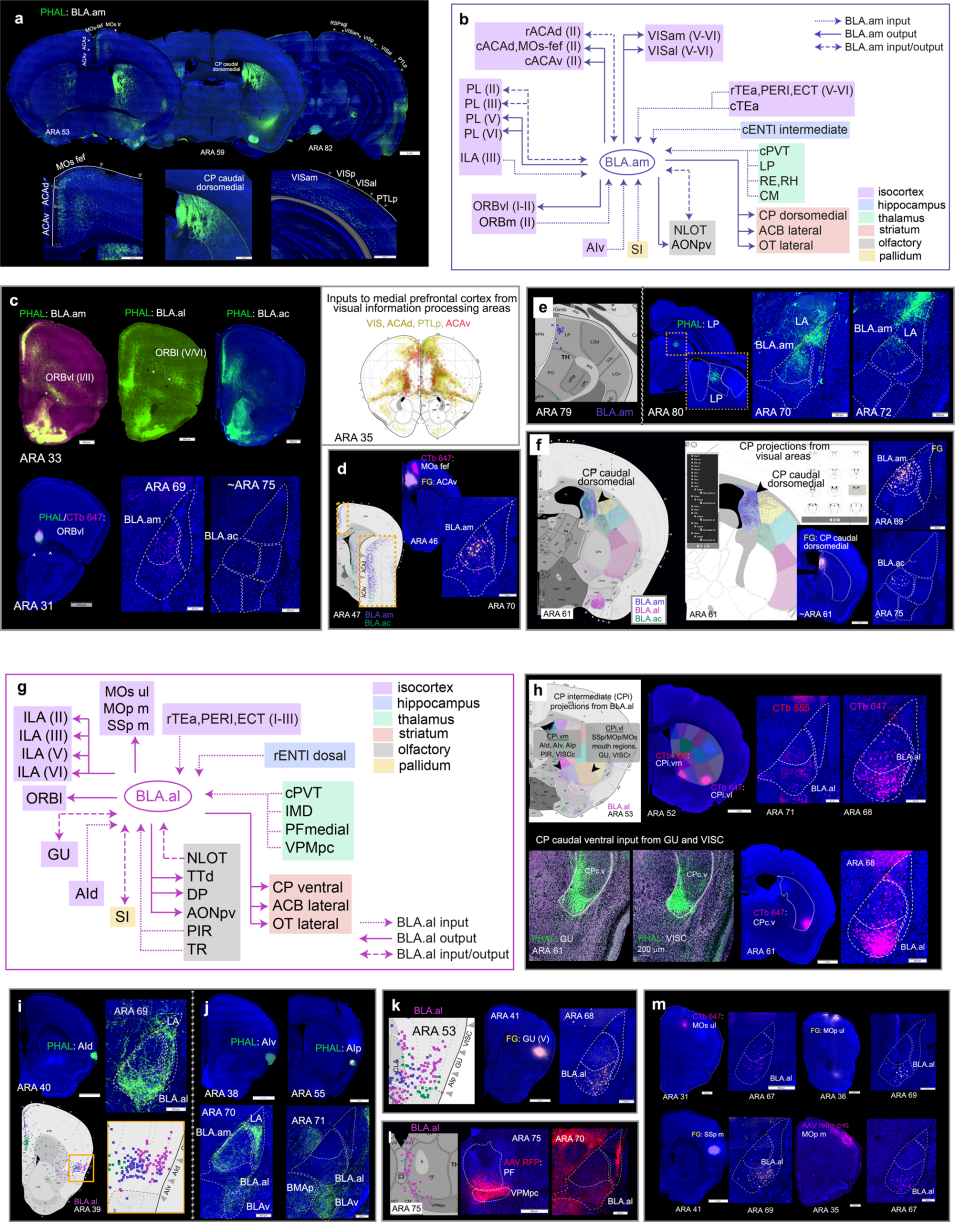
Unique connections of BLA.am and BLA.al neurons. **a** BLA.am projections to visual processing areas. Top panels: whole brain sections with labeled fibers in ACAd, ACAv, MOs-fef, CP caudal dorsomedial, and deep layers of VISam and VISal following a BLA.am PHAL injection. Bottom panels: magnified versions of PHAL labeled fibers in visual associated areas. **b** Summarized brain-wide connections of BLA.am projection neurons. For full list of abbreviations see Table 1. **c** BLAa connections with ORBvl. BLA.am neurons project to ORBvl(I/II), while BLA.al neurons project to ORBl(V/VI). Projections in ORBvl from BLA.ac case were not validated. A CTb ORBvl injection solely back-labels BLA.am neurons confirming a BLA.am→ORBvl connection. ORBvl neurons do not project back to BLA.am as shown by the PHAL ORBvl injection. Schematic adapted from^21^ demonstrates pattern of inputs to frontal cortex from visual information processing areas like VIS, ACAd, ACAv, and PTLp, which is similar to prefrontal input patterns from BLA.am. **d** Anterograde map shows BLA.am projections to ACAv and MOs-fef validated with retrograde injections of CTb and FG (BLA.am→ACAv/MOs-fef). **e** Left: retrograde map with back-labeled neurons in visual LP following a retrograde tracer injection into BLA.am. Right: PHAL injection in visual LP labels fibers in BLA.am, and also LA, confirming LP→BLA.am/LA projections. **f** Left: anterograde map of BLA.am and BLA.ac projection fibers to CP caudal dorsomedial (CPc.dm) at ARA 61 superimposed with CP caudal domains. Right: a screenshot of our cortico-striatal map at ARA 61 showing projections from visual areas like VISp, VISam, VISal, ACA, RSP, and PTLp to CP caudal dorsomedial (http://www.mouseconnectome.org/CorticalMap/page/map/5). FG injection in CPc.dm back-labels BLA.am neurons confirming the BLA.am→CPc.dm connection. **g** Summarized brain-wide connections of BLA.al neurons. For full list of abbreviations see Table 1. **h** BLA.al projections to gustatory/visceral CP. Top left shows anterograde map of BLA.al projection fibers at ARA 53 superimposed with domains of CP intermediate (CPi). BLA.al neurons target CPi.vl and CPi.vm, which also receive input from AI, PIR, VISC, GU, and somatosensory and somatomotor regions associated with mouth regions. Retrograde tracers CTb 555 and CTb 647 in these CP domains back-label projection neurons in BLA.al. Bottom panels: CP caudal ventral (CPc.v), which receives input from GU and VISC is also targeted by BLA.al. A CTb injection in CPc.v back-labels BLA.al neurons to confirm this (BLA.al→CPc.v). **i**. Top: AId neurons target BLA.al and LA as shown with an AId PHAL injection. Bottom: BLA.al retrograde injection back-labels AId neurons (AId→BLA.al). **j** AIv shows weak projections to BLA.am and stronger projections to LA and BLAv. AIp neurons target mostly BLAv and BMAp. **k**. Retrograde map shows neurons in GU that project to BLA.al (GU→BLA.al). A FG injection in GU(V) reveals BLA.al projection neurons target GU [BLA.al→GU(V)]. **l**. Retrograde map shows back-labeled neurons in PF/VPMpc from BLA.al retrograde injection suggesting a PF/VPMpc→BLA.al connection. An anterograde AAV-RFP injection in the thalamic region labels BLA.al, validating the projection. **m** BLA.al projection neurons target somatomotor and somatosensory regions presumably associated with orofacial information processing including the MOs/MOp ul (upper limb) and SSp/MOp m (mouth). Retrograde tracer injections in these regions clearly label BLA.al neurons. See Table 1 for full list of abbreviations.

BLAa connects with other MPF areas. Projection cells in BLA.al provide input to deeper layers of dorsal peduncular area (DP) [BLA.al→DP(II,III)], without receiving much reciprocated input (Supplementary Fig. 7d–f). BLA.am projection neurons strongly target the anterior cingulate area (ACA), and in more caudal sections, the adjacent secondary motor area (MOs) suggested to correspond to frontal eye field (MOs-fef)^21^ (Fig. 5a, d). These projections are directed toward both the dorsal (BLA.am→ACAd) and ventral (BLA.am→ACAv) ACA divisions across its rostral-caudal axis. Only rostral ACAd contains neurons that project back to BLA.am (Supplementary Fig. 7b).

BLA.am neurons specifically target superficial layers of ventrolateral orbital area (ORBvl), while those in BLA.al target deeper layers of the lateral ORB (ORBl) [BLA.am→ORBvl(I/II); BLA.al→ORBl(V/VI)] (Fig. 5c; Supplementary Fig. 7i). Input from medial ORB (ORBm) to BLA.am and BLA.ac are notable [ORBm(II/III)→BLA.am/BLA.ac] with some reciprocity [BLA.am/BLA.ac→ORBm (II-VI)] (Supplementary Fig. 7j).

Notable and topographic projections from agranular insular (AI) cortical areas to BLAa are evident. The BLA.al is the primary recipient of input from dorsal AI (AId) (AId→BLA.al) (Fig. 5i; Supplementary Fig. 7k), while the BLA.am is the primary recipient of inputs from AIv (AIv→BLA.am) (Fig. 5j). Further, BLA.al projection neurons send axons to the gustatory cortical area (GU; BLA.al→GU) and neurons in GU(II-III) project back to BLA.al [GU(II/III)→BLA.al] (Fig. 5k).

The BLAa is directly connected with only a few sensory cortical areas. BLA.am neurons target MOs-fef (Fig. 5a, d) and send sparse terminations to deep layers of secondary visual cortical areas like anteromedial (VISam) and anterolateral (VISal) areas [BLA.am→VISam/VISal(V/VI)] (Fig. 5a; Supplementary Fig. 7c). The BLA.al on the other hand, projects to more rostral parts of MOs, primary motor (MOp), and somatosensory (SSp) regions presumed to be associated with upper limb and orofacial information processing (Fig. 5m).

Projections from BLAa neurons to perirhinal (PERI), ectorhinal (ECT), and temporal association (TEa) areas are sparse. Observable projections from BLA.ac to caudal regions of ECT are noted, although strongest input to ECT arise from LA neurons (Supplementary Fig. 9a). Input from PERI, ECT, and TEa to BLAa are greater and topographically arranged. Neurons in more superficial layers of rostral TEa, PERI, and ECT project primarily to BLA.al [TEa/PERI/ECT(I-III)→BLA.al] (Supplementary Fig. 9b, c), while those in deeper layers target BLA.am [PERI/ECT(V-VI)→BLA.am] (Supplementary Fig. 9d). Strongest input to BLAa from TEa is provided by its caudal region to BLA.am (caudal TEa→BLA.am) (Supplementary Fig. 9e). See Fig. 3i, j for BLA-isocortical connectivity matrices.

Connections between claustrum (CLA) and BLAa are sparse. BLAa domains are the only nuclei with weak projections to the subcortical structure (Supplementary Fig. 9i).

BLAa domains uniquely connect with the entorhinal cortex. Projections from BLAa to lateral entorhinal cortex (ENTl) are weak (Supplementary Fig. 10a), but strong topographic projections from ENTl neurons terminate in BLAa. Cells in rostral (generally dorsal) ENTl regions target BLA.al (rostral/dorsal ENTl→BLA.al) (Supplementary Fig. 10b). More caudal intermediate ENTl cells target BLA.am (caudal/intermediate ENTl→BLA.am), while most ventrally located ENTl cells target BLA.ac (caudal/ventral ENTl→BLA.ac) (Supplementary Fig. 10c). Connections between BLAa and medial ENT (ENTm) are sparse and mostly through BLA.ac (BLA.ac→caudal ENTm; caudal ENTm→BLA.ac) (Supplementary Fig. 10f).

BLAa is connected with regions that process olfactory information. BLA.am and BLA.al cells project to anterior olfactory nucleus posteroventral part (AONpv) (BLA.am/BLA.al→AONpv) (Supplementary Fig. 11a) and more strongly to nucleus of the lateral olfactory tract (NLOT) (BLA.am/BLA.al→NLOT) (Supplementary Fig. 11d). Layer III NLOT cells project back to BLA.am and BLA.al [NLOT(III)→BLA.am/BLA.al] (Supplementary Fig. 11c). BLAa domains also have unique connections with dorsal taenia tecta, (TTd), piriform (PIR; Supplementary Fig. 11e, f), and postpiriform transition area (TR; Supplementary Fig. 12g, h).

**Fig. 6:**
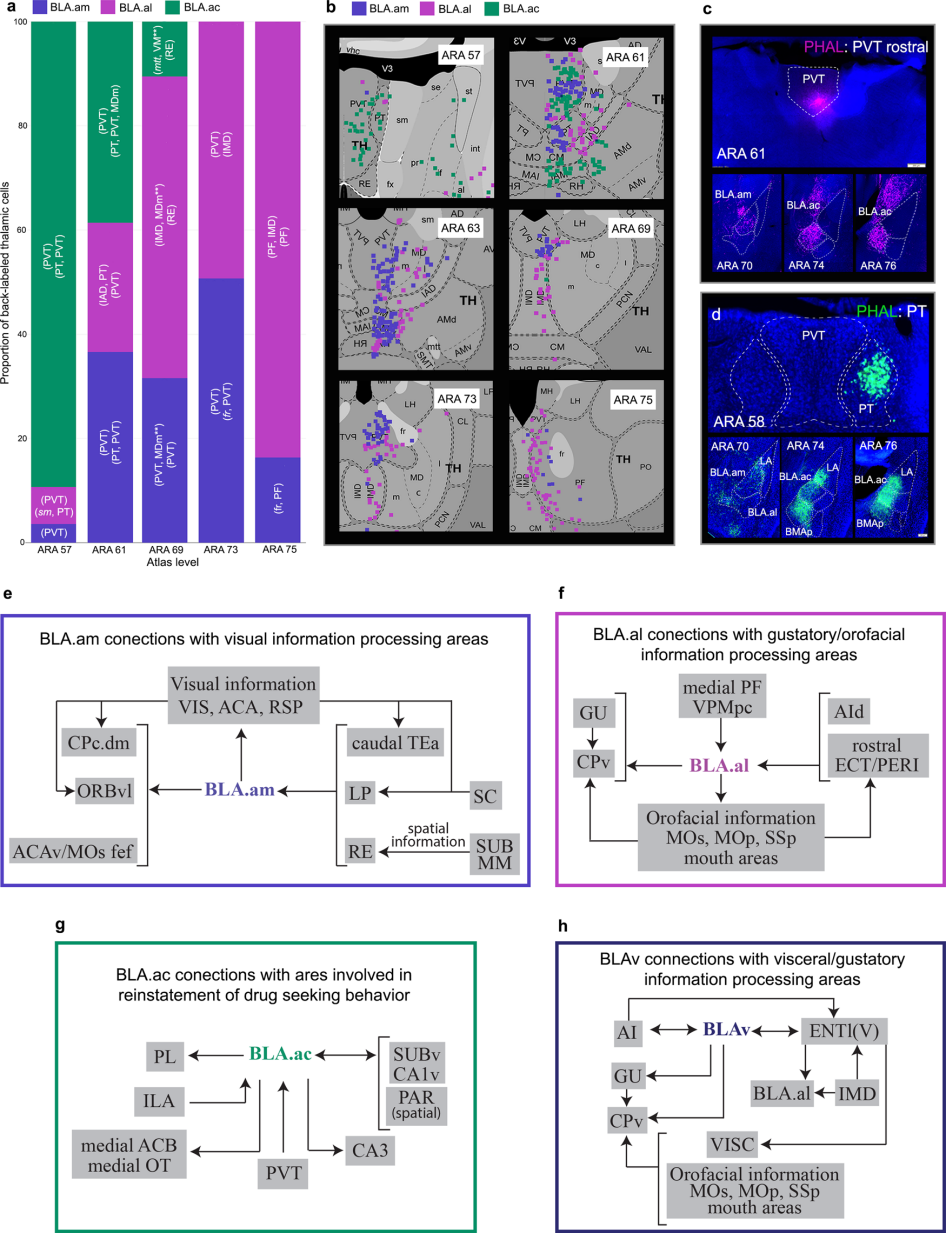
BLAa-thalamic connections and BLAa functional diagrams. **a** Bar chart showing proportion of back-labeled thalamic neurons from representative retrograde tracer injections in BLA.am, BLA.al, and BLA.ac (*n* = 1 each). ROIs for grids with strongest labels from each injection is included. A grid can include multiple ROIs [e.g., (PT, PVT)]. Note the greater proportion of thalamic PVT labeling from BLA.ac injection at rostral levels (ARA 57, 61) versus the larger proportion of thalamic PVT label from BLA.am and BLA.al injections in caudal levels (ARA 73), which substantiates the rostral and caudal PVT distinction. Also, greatest thalamic input to BLA.am is from caudal PVT (caudal PVT→BLA.am), greatest input to BLA.al is from caudal PVT, PF, and IMD (caudal PVT/PF/IMD→BLA.al), and greatest input to BLA.ac is from PT and rostral PVT (PT/rostral PVT→BLA.ac). ** denotes connections that were not validated with anterograde tracing. **b** Retrograde maps from ARA 57–75 showing back-labeled thalamic nuclei from injections in BLA.am, BLA.al, and BLA.ac that corroborate the bar chart in (**a**). **c** PHAL injection in rostral PVT validates the rostral PVT→BLA.ac connection. **d** PHAL injection in PT validates the PT→BLA.ac connection, but also shows strong PT projections to LA and BMAp (PT→BMAp/LA). **e**. BLA.am connections with areas associated with visual processing. **f** BLA.al connections with areas associated with gustatory and orofacial information processing. **g** BLA.ac connections with areas involved in reinstatement of drug seeking behavior. **h** BLAv connections with visceral/gustatory information processing areas. See Table 1 for full list of abbreviations.

Projections from BLAa neurons to thalamus are light; however, all three domains are innervated by the midline thalamic nuclei. Generally, projection neurons in rostral midline nuclei (ARA 57, 61) target BLA.ac, while mid-caudal midline thalamic nuclei (ARA 69, 73, 75) target BLA.am and BLA.al (Fig. 6a). This is most evident with parataenial (PT) and paraventricular (PVT) projections (Fig. 6a, b). The PVT has rostral (rPVT) and caudal (cPVT) subdivisions based on anatomical^22^ and behavioral^23,24^ distinctions. Neurons in rPVT preferentially BLA.ac (rPVT→BLA.ac) (Fig. 6b, c), while those in cPVT primarily project to BLA.am and BLA.al (cPVT→BLA.am/BLA.al) (Fig. 6b; Supplementary Fig. 13a). Additional discriminating thalamic inputs are observed with PT neurons that primarily target BLA.ac (PT→BLA.ac) (Fig. 6b, d) and with intermediodorsal (IMD), parafascicular [PF, medial part (mPF)], and VPMpc neurons that innervate BLA.al (IMD/mPF/VPMpc→BLA.al) (Fig. 5l; Fig. 6b; Supplementary Fig. 13a). BLA.am/BLA.ac also receive input from dorsal anteromedial (AMd), reuniens (RE), rhomboid (RH), and central medial (CM) thalamic nuclei (RE/RH/CM→BLA.am/BLA.ac) (Fig. 6b; Supplementary Fig. 13b). Projections from lateral posterior thalamic nucleus (LP) are unique to BLA.am (LP→BLA.am) (Fig. 5e).

BLAa connects with motor systems. Domain specific BLAa neurons target different regions of the caudoputamen (CP). BLA.al neurons target ventromedial (CPi.vm) and ventrolateral (CPi.vl) regions of intermediate CP (ARA 53), which are also targeted by AI, PIR, VISC, GU, and somatosensory and somatomotor regions related to mouth areas^25^ (BLA.al→CPi.vm/vl) (Fig. 5h). BLA.al neurons also target ventral parts of caudal CP (CPc.v), a region heavily innervated by GU and VISC^25^ (BLA.al→CPc.v) (Fig. 5h). In contrast, BLA.am neurons target dorsomedial regions of intermediate CP (CPi.dm; Fig. 5a; Supplementary Fig. 12b, c), where input from visual cortical areas, ACA, ENTm, and retrosplenial area (RSP) converge^25^ (BLA.am→CPi.dm) (Supplementary Fig. 12d, e). At caudal levels, BLA.am and BLA.ac neurons innervate dorsomedial domains (CPc.dm) (Fig. 5a, f), where visual information also converges^25^ (BLA.am/BLA.ac→CPc.dm) (Fig. 5f). Retrograde injections in dorsal and ventral CP validate these connections and illustrates the segregation of CP projecting BLA.am and BLA.al neuron populations (Fig. 1a; Supplementary Fig. 1c).

Within the nucleus accumbens (ACB) and olfactory tubercle (OT), clear topographic projections originate from BLA.am and BLA.ac neurons. BLA.ac neurons preferentially target medial aspects of ACB and OT (BLA.ac→ACB medial/OT medial), while BLA.am neurons target their lateral aspects (BLA.am→ACB lateral/OT lateral) (Fig. 1f, g). BLA.al neurons sparsely target OT lateral and, more strongly, ACB lateral (BLA.al→OT lateral/ACB lateral) (Fig. 1f, g).

**Fig. 7:**
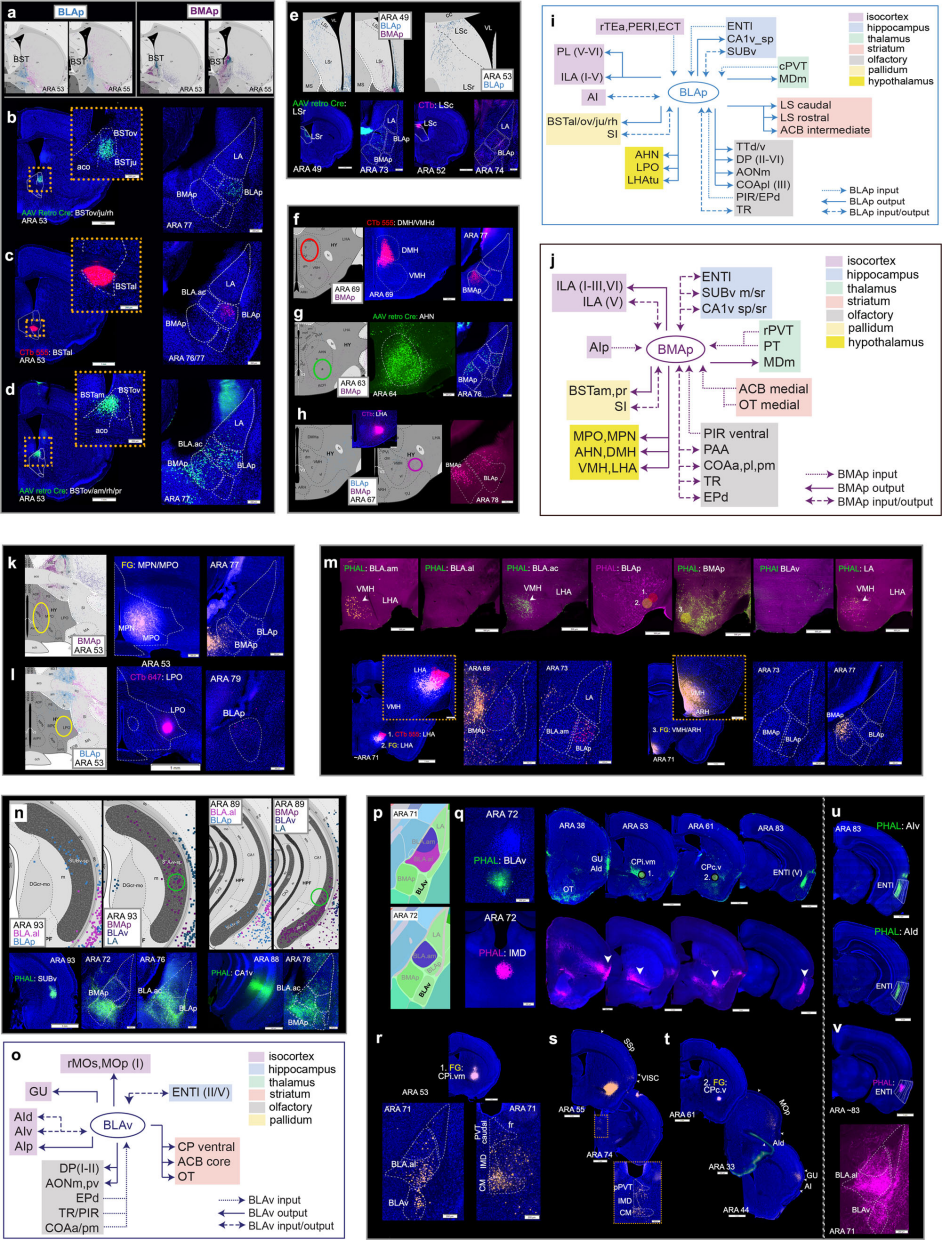
Connections of BLAp, BMAp, and BLAv. **a** Anterograde maps showing BLAp and BMAp neuron projections to BST. BLAp neurons target lateral parts of BST, while those in BMAp target medial BST. **b**–**c** Validation of BLAp projections to BST. Retrograde tracers in BSTov/ju/rh (**b**) and BSTal (**c**) back-label BLAp neurons validating the BLAp→BSTal/ov/ju/rh projections. Note the absence of labels in BMAp. Insets are magnification of injection site regions. **d** Validation of BMAp→BSTam/pr projection. Retrograde tracer injection placed more medially than those in (**b**) and (**c**) (BSTam included) now labels BMAp neurons and some neurons in BLA.ac and LA. Inset shows magnification of injection site region. BST bed nucleus of stria terminalis, BSTov BST oval nucleus, BSTju BST juxstacapsular nucleus, BSTrh BST rhomboid nucleus, BSTal BST anterolateral nucleus, BSTam BST anteromedial nucleus, BSTpr principal nucleus. **e** Top panels: BLAp neurons project to rostral (LSr) and caudal (LSc) lateral septal nuclei and BMAp projects to LSr. Bottom panels validate these connections (BLAp→LSr/c and BMAp→LSr). Retrograde injection in dorsomedial hypothalamic nucleus (DMH: **f**) and anterior hypothalamic nucleus (AHN: **g**) validates BMAp neuron projections to the hypothalamic regions (BMAp→DMH/AHN). **h** Validation of BMAp/BLAp to lateral hypothalamic area (LHA) via LHA retrograde injection. Ellipses on anterograde maps denote location of retrograde injection for validation. **i** Summarized brain-wide connections of BLAp neurons. **j** Summarized brain-wide connections of BMAp neurons. For full list of abbreviations see Table 1. **k** FG injection in medial preoptic nucleus (MPN)/medial preoptic area (MPO) validates BMAp neuron projections to those hypothalamic nuclei (BMAp→MPN/MPO). **l** CTb injection in lateral preoptic area (LPO) validates BLAp neuron projections to the hypothalamic nucleus (BLAp→LPO). Ellipses on anterograde maps to the left denote location of retrograde tracer injections. **m** Top panels: projections from BLA.am, BLA.al, BLA.ac, BLAp, BMAp, BLAv, and LA to the tuberal region of LHA and to ventromedial hypothalamic area (VMH). Strongest projections to VMH are from BMAp neurons, and strongest input to LHA are from BLAp and BMAp neurons. Bottom panels validate these connections. Retrograde tracer injections 1 (CTb) and 2 (FG) in LHA back-label both BLAp and BMAp projection neurons (BMAp/BLA→LHA), while FG injection 3 in VMH back-labels selectively BMAp projection neurons (BMAp→VMH). Insets show magnification of injection site regions. **n** Top panels: retrograde maps showing SUBv back-labeled cells from retrograde tracer injections in BLAp and BMAp (left) and CA1v labeled cells from BMAp retrograde tracer injection (right). Ellipses denote locations of PHAL injections in bottom panels. Bottom panels: PHAL injection in SUBv labels BLA.ac, BLAp, and BMAp validating SUBv→BLA.ac/BLAp/BMAp connections. PHAL injection in CA1v labels fibers in BLA.ac and BMAp, but not BLAp validating the CA1v→BLA.ac/BMAp connections. **o** Summarized brain-wide connections of BLAv neurons. For full list of abbreviations see Table 1. **p** Shows location of BLAv. **q** Top panels show anterograde projections from BLAv, while bottom ones show projections from intermediodorsal thalamic nucleus (IMD). Note the similarity in labeling from the two injection cases in GU (gustatory cortical area), AId (agranular insular area, dorsal part), CPi.vm (CP intermediate, ventromedial), CPc.v (CP caudal, ventral), and ENTl (entorhinal cortical area, lateral part) particularly layer V. **r** FG injection in CPi.vm/vl back-labels BLA.al and BLAv projection neurons (BLA.al/BLAv→CPi.vm/vl), but also neurons in thalamic nuclei CM (central medial) and IMD. **s–t** FG injection in CP caudal ventromedial, where BLAv and IMD project, back-labels neurons in regions proposed to be involved in gustatory/visceral processing like IMD, CM, VISC (visceral cortical area), MOp mouth regions, GU (gustatory cortical area), and AI (agranular insular cortical area). **u** Like the BLAv and IMD, the AI also projects to ENTl(V). **v** A PHAL injection in ENTl(V) shows that it projects back to BLAv, but also to BLA.al [ENTl(V)→BLAv/BLA.al].

Neurons in BLA.al target additional areas like the substantia innominata (SI) (BLA.al→SI) (Supplementary Fig. 12i, k), which provide weak input to BLAa (SI→BLA.am/BLA.al) (Supplementary Fig. 12j, k). Only BLA.ac neurons target bed nuclei of stria terminalis (BST) specifically to its oval (BSTov) and rhomboid (BSTrh) nuclei (BLA.ac→BSTov/rh) (Fig. 7b).

**Fig. 8:**
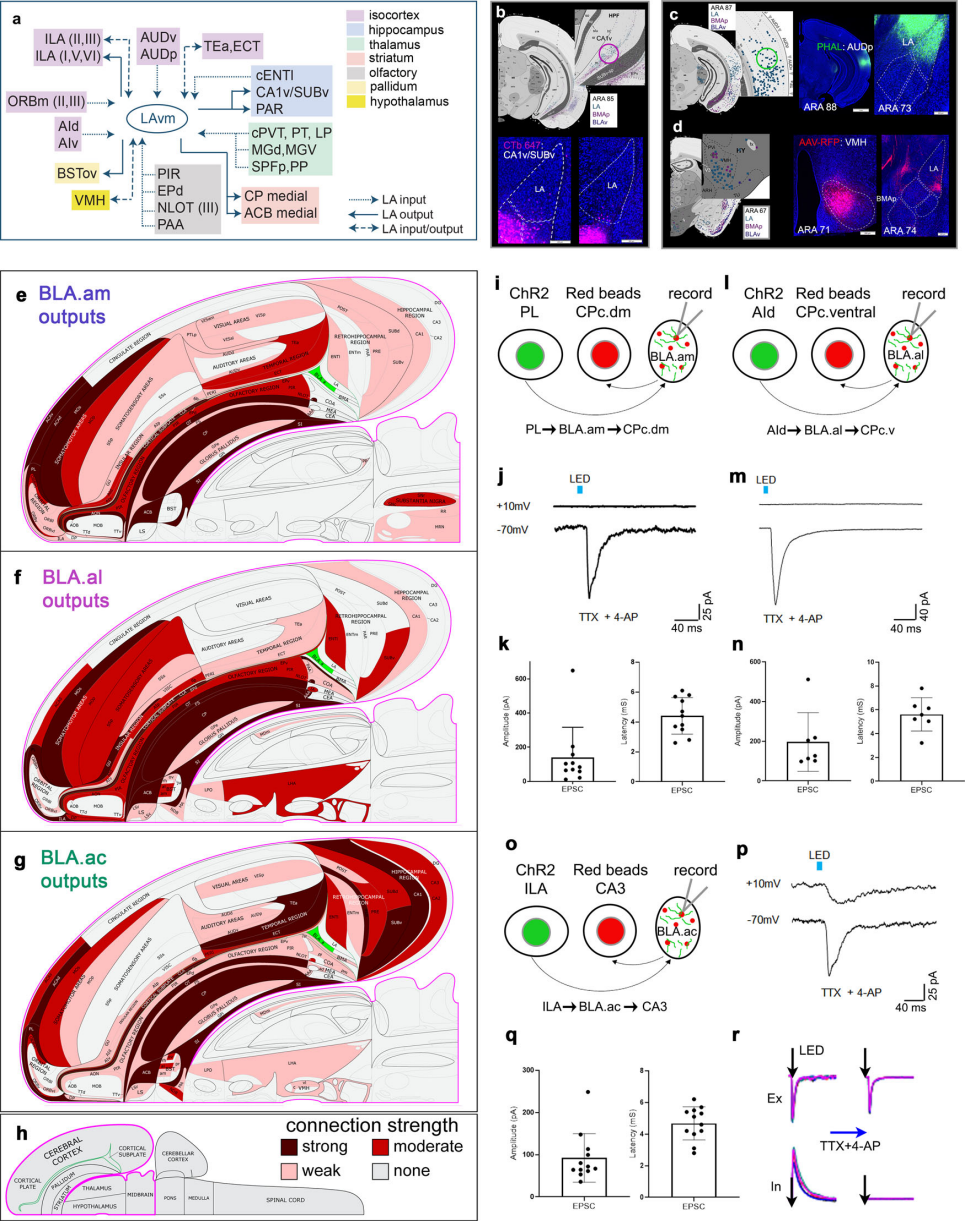
LA connections, flatmaps of BLAa anterograde tracing, and BLAa functional recordings. **a** Summarized brain-wide connections of LA (ventromedial) neurons. For full list of abbreviations see Table 1. **b** Anterograde maps in top panels show weak projections from LA neurons to hippocampal regions SUBv and CA1v. Ellipse denotes location of CTb injection, which validates these sparse connections. **c** Retrograde map shows labeled cells in auditory cortical areas (AUD) following an LA retrograde tracer injection. Ellipse denotes location of PHAL injection in the primary auditory cortical area (AUDp), which shows strong anterograde label in LA validating AUD→LA connections. **d** LA receives strong input from ventromedial hypothalamic nucleus (VMH), as shown by the retrograde map. An AAV-RFP injection in VMH validates the VMH→LA connection showing strong label in LA, but also in BMAp (VMH→BMAp). The outputs of BLA.am (**e**), BLA.al (**f**), and BLA.ac (**g**) domains are represented at the macroscale level (gray matter region resolution) on a partial mouse brain flatmap^75^. The strength values of detected connections were binned into tertiles, and these are represented qualitatively as strong (maroon), moderate (red), weak (pink), and none (gray). **h** Longitudinal half of the entire CNS flatmap showing orientation and major brain divisions. The key to the color codes for connection strength is also shown. **i**–**q** Functional characterization of synaptic innervation of projection-defined BLAa neurons. **i**, **l**, **o** Schematic drawings of experimental design and proposed circuitry. AAV-hSyn-ChR2-YFP (ChR2) is injected into PL (**i**), AId (**l**), or ILA (**o**) to label projection axons in BLA.am, BLA.al, and BLA.ac, respectively. Red retrobeads are injected into CP caudal dorsomedial (CPc.dm), CP caudal ventral (CPc.v), or CA3 to back-label projection neurons in BLA.am, BLA.al, or BLA.ac, respectively. Recordings are made from BLAa neurons labeled with red retrobeads while ChR2 labeled axons in BLA are stimulated. **j**, **m**, **p** LED pulse (5 ms)-evoked averaged response traces of an example neuron recorded at −70mV and +10 mV. Recordings were made in the presence of TTX and 4-AP to block polysynaptic inputs so that only monosynaptic excitatory responses are elicited. Average (± standard deviation) peak amplitude and latency of light pulse evoked EPSCs in red retrobead labeled BLA.am (**k**; 11/12 recorded neurons), BLA.al (**n**; 7/8 recorded neurons), and BLA.ac (**q**; 12/13 recorded neurons) neuron populations. Source data are provided as a Source Data file. **r** Left: excitatory and inhibitory responses (superimposed traces) evoked by blue light stimulation (5 ms). Right, responses from the same recorded cell in the presence of TTX and 4AP to show blockade of IPSPs.

Summarized brain-wide connections of projection neurons in BLA.am (Fig. 5b; Fig. 8e; Supplementary Fig. 14f), BLA.al (Fig. 5g; Fig. 8f; Supplementary Fig. 14g), and BLA.ac (Fig. 4c; Fig. 8g; Supplementary Fig. 14h) are provided. Although tracing experiments were conducted in male mice, BLAa domain-specific output was examined also in female mice, which displayed similar brain-wide patterns to those reported for males (Supplementary Fig. 15).

### Functional pathways of projection-defined BLAa neurons

Optogenetically-assisted circuit mapping ex vivo studies were performed to demonstrate functional pathways through connectionally-defined BLAa subtypes. The BLA affects motor behavior through its outputs to CEA^26^. However, the CP is one of the main projection targets of BLAa neurons, which suggests that BLAa potentially integrates cortical information and affects motor output through CP. Our data suggests PL→BLA.am→CPc.dm and AId→BLA.al→CPc.v connections, which were examined. An ILA→BLA.ac→CA3 pathway also was investigated.

An AAV-hSyn-ChR2-YFP (ChR2) injection was made into PL, while retrograde red microbeads (RR) were injected into CPc.dm to deliver ChR2 to PL axonal terminals in BLA.am and retrogradely label CP projecting BLA.am neurons (Fig. 8i). In slice preparations, whole-cell voltage-clamp recordings were made from CP projecting BLA.am neurons, while PL axons in BLA.am were optically stimulated. A 5-ms pulse of blue light elicited an excitatory current in the recorded neuron clamped at −70 mV in the presence of TTX (1 µM) and 4-AP (1 mM), which eliminate polysynaptic responses^27,28^ (Fig. 8r). Only evoked EPSPs were observed, suggesting excitatory PL to BLA.am projections, which monosynaptically innervate CP projecting BLA.am neurons (Fig. 8k).

To demonstrate an AId→BLA.al→CPc.v connection, ChR2 was injected into AId to label projection terminals in BLA.al, while an RR injection in CPc.v retrogradely labeled BLA.al neurons (Fig. 8l). Recordings were made from CP projecting BLA.al neurons while AId axons in BLA.al were optically stimulated (Fig. 8m, n). Data showed that CP projecting BLA.al neurons are monosynaptically innervated by AId axonal inputs. Similarly, recordings made from CA3 projecting BLA.ac neurons during optical stimulation of ChR2 labeled ILA axons in BLA.ac demonstrated a bi-synaptic ILA→BLA.ac→CA3 connection (Fig. 8o–q).

These studies demonstrate unique functional connections of different BLAa subpopulations defined by their projection targets, which likely contribute to different brain functions (see discussion). They also demonstrate that multi-synaptic pathways can be inferred from traditional tracing studies and subsequently validated through additional experimentation.

### BLAa neuron morphology

**Fig. 9:**
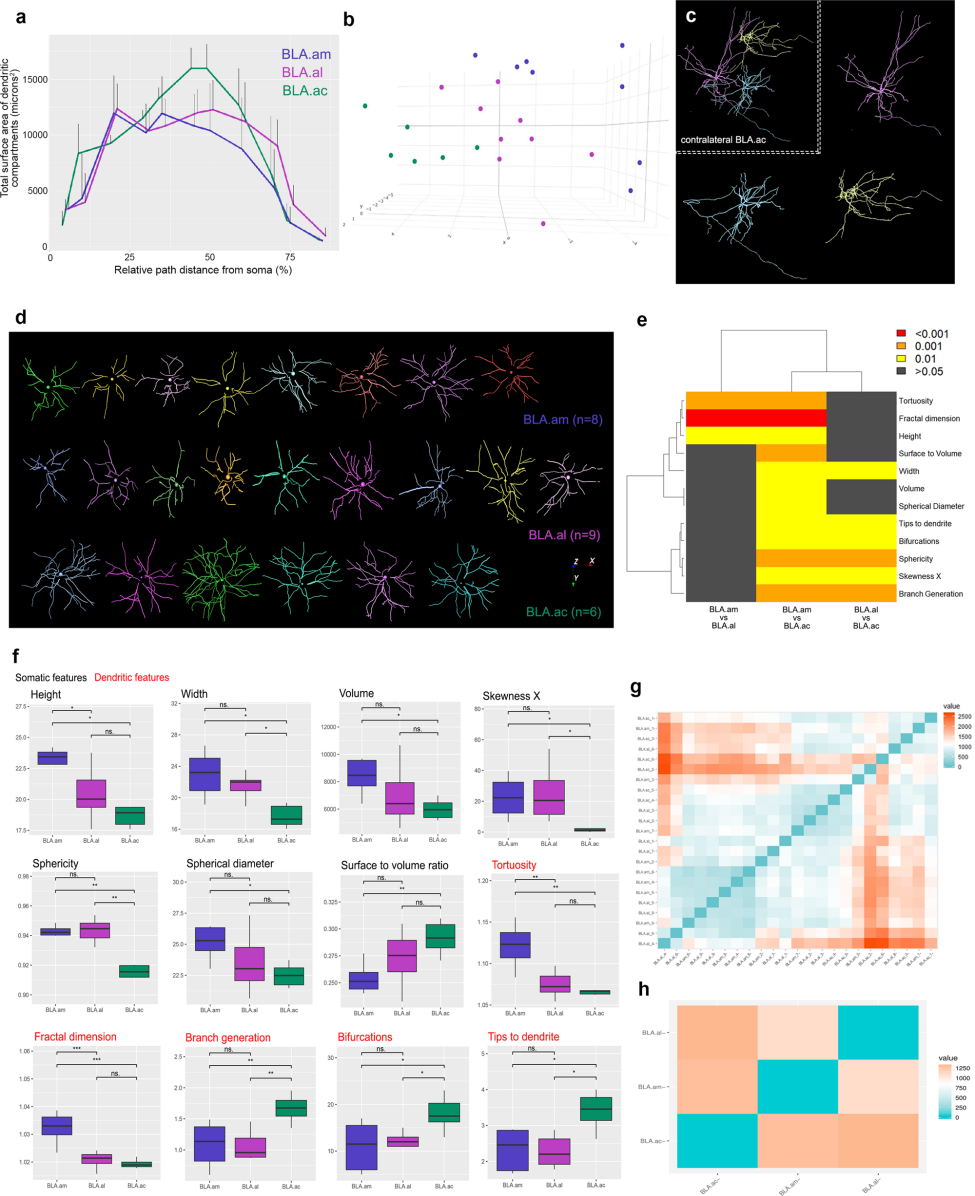
BLAa neuron morphology. **a** Result of Sholl-like analysis to show overall view of BLAa projection neuron dendritic morphology. Graph shows that dendrites of neurons within the BLA.ac have a larger surface area of dendritic compartments at ~45% distance from the cell body compared to dendritic compartments of BLA.am and BLA.al neurons. This larger dendritic surface area suggests the potential for a greater number of synaptic contacts for BLA.ac neurons. **b** 3D scatterplot of principal component analysis (PCA) shows segregation of BLAa domain-specific neurons based on measured morphological features. **c** Contralateral medial accumbens (ACB) projecting BLA.ac neurons. Neurons were labeled via a rabies virus injection in the ACB medial and neurons in contralateral BLA.ac were manually reconstructed. **d** All reconstructed dorsal striatum projecting BLA.am (*n* = 8) and BLA.al (*n* = 9) neurons and ventral striatum projecting BLA.ac neurons (*n* = 6). Reconstructions were used to assess differences in morphological features across the domain-specific projection neurons. **e**–**f** Two-sided pairwise Wilcoxon rank sum tests were run on morphometric data and the parameters that survived the false discovery rate (FDR) correction for multiple testing are reported. Significant group differences are presented with whisker plots in panel (**f**) and the degree of their significance is visualized in a matrix in (**e**). Somatic features are presented in black font and dendritic features are in red. The center line represents the median, the box limits the upper and lower quartiles, and the whiskers the 1.5x interquartile range. * denotes *p* < 0.05, ** *p* < 0.005, *** *p* < 0.0005, and ns = not significant. See Statistical analysis of morphometrics in “Methods” for full statistical reporting. Source data are provided as a Source Data file. The dendrogram on top of the matrix shows the hierarchical clustering of groups based on feature similarity. It suggests that BLA.am and BLA.al neurons differ more from those in BLA.ac than they do from each other. **g** Persistence-based neuronal feature vectorization framework was also applied to summarize pairwise differences between BLA.am, BLA.al, and BLA.ac projection neurons. The strength of the differences is presented as a gradient with blue showing no difference and orange the greatest differences. The individual neuron differences are aggregated in (**h**), which shows once again that neurons within BLA.am, BLA.al, and BLA.ac all differ from one another, but that the greatest difference lies between BLA.am/BLA.al neurons versus BLA.ac neurons.

To assess whether connectionally-distinct BLA.am, BLA.al, and BLA.ac projection neurons are morphologically different, representative cells in each domain were labeled via a G-deleted rabies (RVΔG)^29,30^ injection in CPc.dm (Supplementary Movie 1), CPc.v (Supplementary Movie 2), and in ACB medial (Supplementary Movie 3), respectively (Fig. 1d; NeuroMorpho.org)^31^. Standard measurements for cell body and dendritic morphology were obtained following manual reconstructions (Fig. 9d). Pairwise Wilcoxon rank sum tests were run and parameters that survived false discovery rate (FDR) correction for multiple testing are reported (Fig. 9e, f). A persistence-based neuronal feature vectorization framework was applied to summarize pairwise differences, showing that neurons in BLAa domains each differ from one another, with greatest differences observed between BLA.ac neurons and those in BLA.am and BLA.al (Fig. 9g, h).

Morphological differences between BLA.ac and BLA.am neurons are particularly evident in both dendritic and somatic domains. The branches of BLA.am cells display greater meandering (tortuosity) or space-filling (fractal dimension), whereas dendrites of BLA.ac neurons have more complex arbors, as measured by the tip to dendrites, number of bifurcations, and branch generations (Fig. 9f). Thus, while overall dendritic extent and arbor reach in these subdivisions might be similar, BLA.am neurons appear to be optimized to integrate a larger number of signals within branches, while BLA.ac neurons are better structured to segregate the incoming synaptic inputs among branches. These distinct architectures may be suited to carry out different pattern recognition tasks^32^. Further, BLA.am cell bodies are 20% taller, 30% wider, and more spherical than BLA.ac neurons. While these differences are unlikely to affect computational functions, they could be useful features for future automated high-throughput cell identification^33^.

Spatial distribution and topological analysis further corroborated morphological differentiation among BLAa neurons (Supplementary Fig. 1i). Principal component analysis (PCA) illustrated the segregation of BLAa domain specific neurons based on measured features (Fig. 9b). A three-dimensional Sholl-like analysis showed that in BLA.ac neurons, the distribution of dendritic surface area features greater maxima toward the median of the relative path distance from the soma as opposed to the more uniformly distributed dendritic surface of BLA.am and BLA.al neurons (Fig. 9a).

### Global connections of BLAp

Next, we systematically examined the input-output organization of all other BLA nuclei. Given their close proximity, BLAp and BLA.al tracing data was aggregated and compared to show their unique connections (Supplementary Fig. 16a–c).

Cortical targets of BLAp neurons are located primarily in MPF. BLAp heavily innervates PL and ILA with laminar specificity [BLAp→ILA(I-V)/PL(V-VI)] (Supplementary Fig. 1e; Supplementary Fig. 7d, h). Input from these structures back to BLAp are sparse (Supplementary Fig. 1f). BLAp neurons also strongly target DP [BLAp→DP(II-VI)] without receiving reciprocated input (Supplementary Fig. 7d, f). Sparse connections are observed between AI and BLAp neurons [BLAp→AI(V/VI); AI→BLAp] (Supplementary Fig. 7l). Additional cortical input to BLAp is provided by rostral PERI, ECT, TEa (rostral PERI/ECT/TEa→BLAp) (Supplementary Fig. 9h).

Strongest projections to hippocampal structures are to CA1v_sp, but mostly to SUBv (BLAp→CA1v/SUBv) (Fig. 4d). Input from these neurons back to BLAp are observable (CA1v/SUBv→BLAp) (Fig. 7n). Although projections to ENTl from BLAp neurons are sparse, input from ENTl to BLAp is evident (ENTl→BLAp) (Supplementary Fig. 10g).

Regarding olfactory areas, BLAp neurons target TTd, to a lesser extent its ventral counterpart, TTv (BLAp→TTd/TTv) (Supplementary Fig. 11g), and the medial AON (BLAp→AONm) (Supplementary Fig. 11b). Input from olfactory areas to BLAp is provided by PIR (PIR→BLAp) (Supplementary Fig. 11e, f) and dorsal endopiriform nucleus (EPd→BLAp) (Supplementary Fig. 11h). BLAp neurons project strongly to TR (BLAp→TR), which project back to BLAp (TR→BLAp) (Supplementary Fig. 12g, h).

Unlike BLAa, BLAp does not receive thalamic input. Some cPVT neurons provide sparse input to BLAp (cPVT→BLAp) (Supplementary Fig. 13a). Also unlike BLAa, BLAp neurons target medial subdivisions of the mediodorsal thalamic nucleus (MDm) (BLAp→MDm) (Supplementary Fig. 4e; Supplementary Fig. 13c). Most BLA→MD projections^20^ arise from BLAp and BMAp and not from BLAa^13^.

BLAp connects with motor systems. Similar to BLAa, BLAp cells provide input to ACB, specifically the intermediate part (BLAp→intermediate ACB) (Supplementary Fig. 12a). Unlike BLAa neurons, cells in BLAp do not strongly target CP (Supplementary Fig. 12b) or OT (Fig. 1f). Instead, BLAp neurons target lateral septum (LS) (Fig. 7e), bed nucleus of the stria terminalis (BST), and hypothalamus. Specifically, BLAp targets lateral BST including the anterolateral (BSTal), oval (BSTov), juxstacapsular (BSTju), and rhomboid (BSTrh) nuclei (BLAp→BSTal/ov/ju/rh) (Fig. 7b, c), providing less input to anteromedial BST (BSTam) (Fig. 7a). Hypothalamic structures targeted by BLAp include lateral preoptic area (LPO) (Fig. 7l), anterior hypothalamic nucleus (AHN) (Fig. 7g), and lateral hypothalamic area (LHA) (BLAp→LPO/AHN/LHA) (Fig. 7m). Like BLA.am and BLA.al, BLAp neurons provide input to ventral pallidal structures like SI, which in turn, provide input back to BLAp (BLAp→SI; SI→BLAp) (Supplementary Fig. 12i–k).

Summarized brain-wide connections of projection neurons in BLAp are provided in a wiring diagram (Fig. 7i).

### Global connections of BLAv

BLAv (Fig. 7p) displays a unique connectivity architecture that is strikingly similar to the thalamic IMD nucleus. Strongest cortical targets of BLAv neurons include rostral AId, AIv, and GU (BLAv→rostral AId/AIv/GU) (Fig. 7q). In turn, AId, AIv, and AIp neurons all target BLAv (AId/AIv/AIp→BLAv) (Fig. 5j; Supplementary Fig. 7l). In rostral cortical areas, BLAv neurons target layer I of MOp and MOs motor cortices (Supplementary Fig. 4f). BLAv connections to MPF are through DP [BLAv→DP(I/II)] (Supplementary Fig. 7d, g).

Another common BLAv and IMD target is a selective region within rostral ENTl layer V [BLAv→ENTl(V)] (Fig. 7q), which also receives input from AI, GU, and VISC (Fig. 7u: Supplementary Fig. 10d, e). Whereas BLAa domains sparsely target ENTl, input from BLAv to ENTl(V) is dense, with reciprocated input [ENTl(V)→BLAv] (Fig. 7v).

BLAv and IMD neurons target similar motor regions. Within intermediate CP (ARA 53), BLAv neurons target the ventromedial (CPi.vm) region, which is also innervated by BLA.al (BLAv→CPi.vm) (Fig. 7q–s). In caudal CP (ARA 61), BLAv neurons target CPc.v (Fig. 7q, t). BLAv and IMD neurons target ACB core (BLAv→ACBcore) (Supplementary Fig. 12a) and some projections from BLAv neurons to OT are present (BLAv→OT) (Fig. 1f; Fig. 7q).

Most prominent olfactory connections for BLAv is through PIR (PIR→BLAv) (Supplementary Fig. 11e, f). Neurons in EPd (EPd→BLAv) (Supplementary Fig. 11h), TR (TR→BLAv) (Supplementary Fig. 12h), anterior (COAa) and posterior medial (COApm) cortical amygdala areas (COAa/COApm→BLAv) (COAa/pm→BLAv) (Supplementary Fig. 14b, d) provide input to BLAv. In turn, BLAv neurons target AONm and AONpv (BLAv→AONm/pv) (Supplementary Fig. 11b). Summarized brain-wide connections of BLAv projection neurons are provided (Fig. 7o).

### Global connections of LA

LA projection neurons provide input to ILA [LA→ILA(I-VI)] and neurons in ILA project back to LA [ILA(II/III)→LA] (Supplementary Fig. 1e, f). LA also provides input to ORBm (LA→ORBm), which is reciprocated [ORBm(II/III)→LA); LA→ORBm] (Supplementary Fig. 7j). Inputs to AI are sparse, although AId and AIv neurons target LA (AId/AIv→LA) (Fig. 5i, j). Strongest cortical projections from LA neurons are to ECT and TEa (LA→ECT/TEa) (Supplementary Fig. 9f), which in turn target LA (ECT/TEa→LA) (Supplementary Fig. 9g). LA also receives strong input from primary (AUDp) and ventral (AUDv) auditory areas (AUDp/AUDv→LA) (Fig. 8c).

Weak hippocampal connections were detected for LA neurons (ENTl→LA; Supplementary Fig. 10i) (LA→CA1v/SUBv; Fig. 8b) (LA→PAR; Fig. 4a).

LA does not project strongly to olfactory regions although neurons in olfactory areas like PIR, EPd, and NLOT(III) provide input to LA [PIR/EPd/NLOT(III)→LA] (Supplementary Fig. 11c, e, h). The cortical olfactory area, piriform-amygdala area (PAA), also targets LA (PAA→LA), while posterior lateral COA (COApl) receives some input (LA→COApl) (Supplementary Fig. 14c).

Thalamic structures that provide input to LA include cPVT, PT, and LP (caudal PVT/PT/LP→LA) (Fig. 6d; Supplementary Fig. 13d–f). Strongest inputs originate from dorsal (MGd) and ventral (MGv) medial geniculate neurons (MG) and SPFp (subfascicular nucleus, parvocellular part) and PP (peripeduncular nucleus) nuclei (MGd/MGv/SPFp/PP→LA) (Supplementary Fig. 13g, h).

Connections from LA to motor systems include a specific region in ACB medial (LA→ACB medial) (Supplementary Fig. 12a), BSTov (LA→BSTov) (Fig. 7d), and CP (LA→CP medial) (Supplementary Fig. 12b). Finally, although LA neurons provide weak input to hypothalamic VMH (LA→VMH) (Fig. 7m), VMH neurons send strong projections back to LA (VMH→LA) (Fig. 8d). Summarized brain-wide connections of LA projection neurons are provided in Fig. 8a.

### Global networks of BMAp

BMAp neurons target ILA [BMAp→ILA(II-VI)] and in turn, receive input from the MPF area [ILA(V)→BMAp] (Supplementary Fig. 1e, f). BMAp neurons also provide input to ORBm (BMAp→ORBm) (Supplementary Fig. 7j) and receive input from AIp cells (AIp→BMAp) (Fig. 5j).

Besides BLAv, BMAp neurons are the only BLA projection neurons to provide strong input to ENTl [BMAp→ENTl(V/VI)] (Supplementary Fig. 10h). Like all other BLA nuclei, BMAp receives input from ENTl (ENTl→BMAp) (Supplementary Fig. 10h). Similar to BLA.ac and BLAp, BMAp neurons project to stratum radiatum of ventral CA1 (CA1v_sr) and to SUBv, except BMAp neurons target specifically the molecular (SUBv_m) and stratum radiatum (SUBv_sr) layers of SUBv, a distinguishable feature of BMAp injections (BMAp→CA1v_sr/SUBv_m/sr) (Fig. 4d). Neurons in CA1v_sp and especially SUBv project back to BMAp (CA1v_sp/SUBv→BMAp) (Fig. 7n).

For olfactory processing, the BMAp receives strong input from ventral PIR (PIRv→BMAp) (Supplementary Fig. 11e). Of all nuclei, the BMAp is most strongly connected with olfactory cortical areas and receives input from the PAA (PAA→BMAp) (Supplementary Fig. 14c), TR (TR→BMAp) (Supplementary Fig. 12h), and the anterior (COAa) (Supplementary Fig. 14b), posterior lateral (COApl) (Supplementary Fig. 14c), and posterior medial (COApm) (Supplementary Fig. 14d) cortical amygdala areas (COAa/COApl/COApm→BMAp). BMAp neurons project back to each of these areas (BMAp→COAa/COApl/COApm/PAA/TR) (Supplementary Fig. 14a, c, e; Supplementary Fig. 12g). Strong connections between EPd and BMAp are evident (BMAp→EPd; EPd→BMAp) (Supplementary Fig. 11h).

BMAp-thalamic connections were observed. BMAp neurons send weak projections to MDm (BMAp→MDm) (Supplementary Fig. 13c) and, like the BLA.ac, receive input from rPVT (rPVT→BMAp), and PT (PT→BMAp) (Fig. 6d; Supplementary Fig. 13e).

Within the ventral striatum, BMAp neurons target the ACB medial, where inputs from BLA.ac, BLAp, and LA also terminate (BMAp→ACB medial) (Supplementary Fig. 12a). BMAp neurons sparsely project to OT medial (BMAp→OT medial) (Fig. 1f). In addition, BMAp neurons share connections with SI (BMAp→SI; SI→BMAp) (Supplementary Fig. 12i–k) and BST, particularly with the anteromedial (BSTam) and principal (BSTpr) nuclei (BMAp→BSTam/BSTpr) (Fig. 7a, d). As such, while BLAp preferentially targets lateral anterior BST, BMAp neurons target medial anterior BST (Fig. 7a–d).

Finally, BMAp neurons send appreciable projections to the medial hypothalamic column, such as the medial preoptic area (MPO), medial preoptic nucleus (MPN) (Fig. 7k) (BMAp→MPO/MPN), dorsomedial hypothalamus (DMH) (Fig. 7f), AHN (Fig. 7g), LHA (Fig. 7h), and VMH (Fig. 7m) (BMAp→DMH/AHN/LHA/VMH).

Summarized brain-wide connections of BMAp neurons are provided (Fig. 7j).

## Discussion

In this work, we provided a comprehensive, systematically collected, dataset on mouse BLA connectivity. Discrete BLAa projection neuron types were identified based on their connectivity characteristics. These distinguishable connections provide insight into the fundamental organizational principles of amygdalar circuits that regulate different behavioral output. Some potential hypotheses are presented below.

The BLA.am is in a network of structures associated with visual information processing and eye movement (Fig. 6e). Visual information can reach the BLA.am from caudal TEa, which receives abundant visual and auditory inputs^21^, from the visual LP thalamic nucleus, and from thalamic RE, which contains head direction cells^34^. In turn, BLA.am projections to motor areas like CPc.dm can regulate behavioral output. The CPc.dm is implicated in visual information processing given that it integrates inputs from areas like VIS, ACA, and RSP^25^. The BLA.am also sends input to deep layers of secondary visual areas (VISam, VISal), the ACAv, MOs-fef, and ORBvl. The ORBvl is in a cortical network that processes visual and spatial information. BLA connections to primary visual cortex are reported in primates^35^ although, to our knowledge, not in mice. The relevance of these connections are apparent in the context of primate studies showing BLA involvement and facial expressions^36^. They may also be relevant in rodents. Although BLA (BLAa, BLAp) unimodal neurons responsive only to visual cues are sparse, neurons responsive to a variety of sensory modalities, including visual, auditory, somatosensory, and gustatory, are dispersed throughout^37^. Further, significantly more multimodal neurons that respond to stimuli previously paired in a conditioning task are located in BLA (BLAa, BLAp). Our data suggests multimodal neurons involving visual information may be in BLA.am. Since re-evaluation of stimuli for outcome prediction is BLA dependent^38^, it is a reasonable assumption that BLA neurons would be equipped with the ability to survey and communicate about stimuli of various modalities, including those that are visual in nature.

The BLA.al has connections that suggest its role in gustatory information processing (Fig. 6f). Distinct areas within the insular cortex (AId, AIv, AIp, and GU) are shown to code palatable and unpalatable tastants, with neurons responding to sweet tastants located more rostrally than those that respond to bitter^39^. Unique amygdalar projections from the “sweet” cortical area to BLA process the hedonic valence of the tastant^40^. Our data showed that the strongest projections from rostral AId (location of sweet neurons) are to BLA.al. BLA cells targeting AId and GU also are primarily located in BLA.al. In addition, BLA.al receives input from rostral ECT/PERI, which share extensive connections with somatic sensorimotor mouth areas^21^. BLA.al neurons, in turn, specifically target CP domains that receive convergent inputs from the AI, GU, and somatic and somatomotor mouth regions^25^, suggesting a role for BLA.al in feedback mechanisms potentially involving these regions. In fact, we showed that AId fibers directly innervate BLA.al projection neurons that target CPc.v, providing a functional circuit for gustatory information processing and behavioral motor output. Thalamic input to BLA.al comes from the caudal PVT, the IMD, medial part of PF, and VPMpc. The medial part of the thalamic PF nucleus was recently shown to project to AI^41^, the caudal PVT receives input from the AI^42^, and the VPMpc receives gustatory information from the medial parabrachial nucleus (PBN) within the gustatory taste pathway^43^. Taste neurons have been identified in BLA^44^ and following odor-taste associations, the number of BLA neurons that respond to both stimuli dramatically increases^45^. BLA.al could be a candidate region in which these conditioned responsive cells involving taste stimuli are located. BLAp and BLAv are additional possibilities.

Unlike BLA.am and BLA.al, BLA.ac neurons are connected with hippocampal regions like SUBv, CA1v, but most particularly with CA3 and PAR. Connections with these latter two regions are exclusive to BLA.ac neurons. Compared to BLA.am and BLA.al, which provide input to ACB lateral and OT lateral^46^, BLA.ac neurons provide input predominantly to ACB medial shell and OT medial^47^. Unique thalamic input to BLA.ac is provided by PT and rostral PVT^22^. These BLA.ac neuronal connections potentially suggest a role in context-induced reinstatement of drug seeking behavior (Fig. 6g). Reexposure to the environment in which drugs are self-administered facilitates relapse following abstinence. This contextually promoted vulnerability is modeled in laboratory animals using context-induced reinstatement. The BLA^5^, medial shell ACB^48^, SUBv^49^, CA1v^50^, CA3^51^, PVT^52^, and PL/ILA^5,53^ are contributors to the circuitry that meditates contextual reinstatement. Specifically, interactions between BLA and ACB medial shell^54^, PL/ILA^55,56^, and PVT^52^ are implicated. Here we showed that ILA fibers innervate BLA.ac projection neurons that target CA3, providing a functional circuit among these regions. Importantly, similar to the ACB medial shell, OT medial, and not OT lateral, is important for reinforcing effects of psychostimulants^57^. The potential contributions of a ACB medial + OT medial complex in context-induced reinstatement has been suggested^48^. BLA.ac-PAR connections are in accord with this hypothesis given that PAR contains rich amounts of grid cells, head direction cells, and border cells^58^ for spatial encoding. Additional hypotheses regarding BLA.ac are suggested by its connectivity profile. Optical stimulation of projections from BLA to ventral hippocampus are shown to be anxiogenic^8^ and to reduce social interaction^59^. The BLA.ac is a likely candidate for exploring these particular circuits given its connections with ventral hippocampus. Other candidates for this hypothesis would include the BLAp and BMAp.

The BLAv is in a network associated with gustatory and visceral information processing including AI, GU, IMD, ENTl, and CP (Fig. 6h). There is a striking similarity between BLAv projection targets and those of the thalamic IMD, which is grouped into the dorsal midline thalamic nuclei speculated to be involved in viscero-limbic functions^60^. These shared targets include the AI, ventral medial parts of intermediate CP, ventral parts of caudal CP, and a distinct ENTl(V) region. The ventral intermediate CP is a region where inputs from AI, GU, VISC, and somatomotor mouth regions converge^25^ (Fig. 7s, t). The ventral caudal CP receives densest input from VISC^25^, but also from GU, and from thalamic CM, a region known for its dense connections with GU and VISC^60^ (Fig. 7s, t). The ENTl(V), like BLAv, also receives strong input from AI.

BLA is most recognized for its involvement in emotional fear conditioning. This emotional conditioning and its resistance to extinction mimic the persistent pathological fears that manifest in post-traumatic stress disorder (PTSD). The BLA, ILA, PL, and hippocampus (HPF) are all implicated in fear conditioning^1,2^ and distinct basal amygdalar cell populations connected with either the HPF or medial prefrontal areas are activated under either conditions of fear or extinction, respectively^61^. Therefore, intricate connections among BLA, MPF, and HPF can guide hypotheses regarding roles of subregional neuron connections in fear acquisition/extinction circuits.

The ILA is predominantly involved in fear extinction, while PL is implicated in persistent fear expression^62,63^. PL projecting BLA neurons are activated during fear expression, while ILA projecting BLA neurons show increased activity during extinction expression^6^. Our data showed that BLA→ILA neurons are primarily located in BLA.al, BLA.ac, BLAp, BMAp, and LA and target ILA with laminar specificity. PL projecting neurons are located primarily in BLA.am, BLA.ac, and BLAp. Input from PL to BLA are strongest to BLA.am and we showed a functional PL→BLA.am→CPc.dm that could potentially mediate motor output of PL-BLA.am behavioral mediation. Strong ILA→LA connections are reported in the rat, but our data showed denser ILA input to BLA.ac and BMAp. HPF projections to BLA are shown to mediate contextually dependent fear extinction^64^. Our data suggests BLA.ac, BLAp, and BMAp as likely candidates given their connections with CA1v and SUBv. Aside from CA1, CA3v, and SUBv, BLA.ac neurons also strongly connect with PAR, a primary site for spatial encoding^58^ relevant for contextual information processing.

## Methods

### Subjects

Data from 8-week old male (*n* = 183) and female (*n* = 8) C57BL/6J mice (Jackson Laboratories) were used to trace the 245 pathways reported in this work. Animals were housed in pairs in a temperature (21–22 °C), humidity (51%), and light controlled (12-hr light:12-hr dark cycle) room. The mice were allowed at least 1 week to adapt to their living conditions prior to stereotaxic surgeries for the delivery of tracers. Subjects had ad libitum access to tap water and mouse chow throughout the experiments. All experiments were conducted according to the regulatory standards set by the National Institutes of Health Guide for the Care and Use of Laboratory Animals and by the institutional guidelines set by the Institutional Animal Care and Use Committee at USC.

### Surgical methods

Anterograde and retrograde tracers were delivered to anatomically delineated regions across the brain to assess their connectivity patterns^25,65^. Stereotaxic surgeries for tracer infusions were performed under isoflurane anesthesia (Hospira, Inc.). Mice were initially anesthetized in an induction chamber primed with isoflurane and were subsequently mounted to the stereotaxic apparatus where they were maintained under anesthetic state via a vaporizer (Datex-Ohmeda). The isoflurane was vaporized and mixed with oxygen (0.5 L/min) and nitrogen (1 L/min). The percent of isoflurane in the gas mixture was maintained between 2 and 2.5. Tracers were delivered iontophoretically using glass micropipettes whose outside tip diameters measured ~10–30 µm. A positive 5 µAmp, 7-second alternating injection current was delivered for 10 min (Stoelting Co.).

### Tracing strategies

Anterograde tracers included *Phaseolus vulgaris* leucoagglutinin (PHAL; 2.5%; Vector Laboratories) and adeno-associated viruses encoding enhanced green fluorescent protein (AAV GFP; AAV1-hSyn-EGFP-WPRE; 2.3 ×10^13 GC/mL; Addgene #105539) or tdTomato (AAV RFP; AAV1-CAG-tdTomato-WPRE; 2.0 × 10^13^ GC/mL; Addgene #105554). Retrograde tracers included cholera toxin subunit b conjugates 647, 555, and 488 (CTb; AlexaFluor conjugates, 0.25%; Invitrogen), Fluorogold (FG; 1%; Fluorochrome, LLC), and AAVretro-hSyn-Cre-WPRE (AAV retro Cre; 1.6 × 10^13^ GC/ml; Addgene #105553). Anterograde and retrograde tracers were injected either in combination (e.g., co-injection of PHAL with CTb 647) or individually in a triple anterograde (PHAL, AAV-GFP, AAV-RFP) or a quadruple retrograde (CTb 647, CTb 555, CTb 488, or FG, AAV retro Cre) injection design.

For morphological information from BLAa domain specific projection neurons, G-deleted rabies virus injections (RVΔG-4tdTomato and RVΔG-4eGFP) were made into downstream targets of the domains. Cloning of pRV∆G-4tdTomato^29^ (Addgene #52500) and pRV∆G-4GFP^30^ (Addgene #52487) has been described. Production of B19G-enveloped rabies virus was done as described previously^29,30,66^ but using helper plasmids pCAG-B19N (Addgene #59924), pCAG-B19P (Addgene #59925), pCAG-B19G (Addgene #59921), pCAG-B19L (Addgene #59922), and pCAG-T7pol (Addgene #59926) for the rescue step^29^. The final titers were 2.14e10 infectious units/ml for RV∆G-4tdTomato(B19G) and 1.51e11 infectious units/ml for RV∆G-4GFP(B19G), as determined by infection of HEK 293 T cells^30^.

### Tissue processing and imaging in 2D

Either one (PHAL, CTb, FG, RVΔG) or three (AAVs) weeks was allowed for tracer transport after which animals were perfused and their brains were extracted. For all but the morphology studies involving RVΔG injections, a 2D tissue processing workflow was followed. After an overdose injection of sodium pentobarbital, each animal was transcardially perfused with 50 ml of 0.9% NaCl followed by 50 ml of 4% paraformaldehyde solution (PFA; pH 9.5). The brains were post-fixed in 4% PFA for 24–48 h at 4 °C after which they were embedded in 3% Type I-B agarose (Sigma-Aldrich) prior to sectioning. Four series of coronal sections were sliced at 50-µm thickness with a compresstome (VF-700, Precisionary Instruments, Greenville, NC) and prepared for immunofluorescence staining.

One series of a 1-in-4 series of sections was immunostained for the antigen of interest (PHAL or AAV retro Cre) using the free-floating method. Briefly, sections were transferred to a blocking solution containing normal donkey serum (Vector Laboratories) and Triton X-100 (VWR) for 1 h. Following three 5-minute rinses, sections were incubated in a KPBS solution comprised of donkey serum, Triton, and the appropriate antibody [1:1000 rabbit anti-PHAL antibody (Vector Laboratories, #AS-2300) or 1:4000 mouse anti-Cre recombinase antibody (EMD Millipore, #MAB3120)] for 48–72 h at 4 °C. Sections were rinsed 3 times in KPBS and then soaked for 3 h in the secondary antibody solution, which contained donkey serum, Triton, and a 1:500 concentration of anti-rabbit IgG conjugated with Alexa Fluor® 488 or 647 (Invitrogen, 488: #A-21206; 647: #A-31573) for PHAL staining. For Cre recombinase staining, the secondary solution contained donkey serum, Triton, and a 1:500 concentration of anti-mouse IgG conjugated with Alexa Fluor® 488 or 647 (Life Technology, 488: #A-21202; 647: #A-31571). Following 3 KBS rinses, the sections were counterstained with a fluorescent Nissl stain, NeuroTrace® 435/455 (NT; 1:500; Invitrogen, #N21479). The sections were then mounted and coverslipped using 65% glycerol.

Sections were scanned at ×10 magnification as high-resolution virtual slide image (VSI) files using an Olympus VS120 with identical exposure parameters across all cases.

### Post image acquisition processing for 2D data

Sections from each case were assigned and registered to a standard set of 32 corresponding Allen Reference Atlas levels ranging from 25–103. Variations in tissue cut angle can lead to challenges in atlas level assignments for individual sections. For example, for sections that contain both labeling and an injection site, the fittest corresponding atlas level for the location of tracer labels may differ from the level that best fits the injection site. As such, these sections were registered twice; once to the atlas level most ideal for the labeling and a second time to the level most fit for the injection site. In addition, atlas level assignments were made to optimize accurate annotation. Consequently, attention was particularly focused on regions that contained labeling. For example, sometimes due to cut angle discrepancies, the dorsal and ventral parts of a tissue section would ideally get matched and registered to different ARA levels, which technical limitations preclude. For such cases, the atlas levels most ideal to the location of the labels were selected. As such, the annotation would accurately reflect the anatomic location of the labeled cells or fibers.

Threshold parameters were individually adjusted for each case and tracer. Conspicuous artifacts in the threshold output were filtered. For a small proportion of cases, accurate alignment of sections to the atlas template was precluded due to limitations of our registration or due to the cut angle of the section. Consequently, label would locate outside of correct ROI. For these cases, position of the label was adjusted to the correct ROI location. Further, oversaturation at the injection site prevents the detection of labeled fibers and boutons at the injection sites (BLAa) and their surrounding areas (LA, CEA). Therefore, with this injection strategy, it is difficult to assess intra-amygdalar connections especially within the BLA complex and the CEA. Due to their proximity to the injection sites, these areas appear as dense label in the threshold output subsequently affecting annotation and analysis. To obviate this, these areas of potentially false dense label at or surrounding the injection sites (specifically the BLA complex and CEA) were filtered out at the thresholding stage for all BLAa cases.

Intra-amygdalar connections were excluded from the analysis given the challenge to accurately distinguish labels from background in nuclei located close to injection sites.

Generally, only connections that were validated are reported. For certain computer-generated visualizations (e.g., bar charts, matrices), non-validated connections were reported. Such cases are clearly indicated on the visualizations. In addition, sometimes tracers produce very weak labeling in ROIs, which render interpretation of the data difficult. For example, tracers will label only a few (~2–3) axon terminals or cells within an ROI. Such connections are not reported.

### Reproducibility of data

For most figures, labels from representative cases are presented. However, injections in all BLA nuclei were repeated. The following are sample sizes for anterograde tracer injections: BLA.am (*n* = 8), BLA.al (*n* = 6), BLA.ac (*n* = 7), BLAv (*n* = 2), BLAp (*n* = 2), BMAp (*n* = 2), and LA (*n* = 2). For retrograde tracer injections, repeated sets of injections were made for each BLA nucleus. The consistency of labeling across repeated cases is clear from manual analysis of the data (Supplementary Fig. 4b). The label similarity also is evidenced in the output of the 2D hierarchical clustering analysis (described below), which groups injection sites based on the commonality of their projection targets. As can be seen in Supplementary Fig. 6a, BLA.am injections grouped together, while BLA.al and BLA.ac injections formed their individual groups. This was the case despite the different anterograde tracers used across the experiments (PHAL or AAV). As additional confirmation of reproducibility, BLA.am, BLA.al, and BLA.ac anterograde injections were repeated in female mice. Supplementary Fig. 15a–c shows the similarity in brain-wide label patterns from the same BLAa domain in males and females.

### Tracer label validation

Importantly, injections reported for the BLAa domains are not entirely confined. For instance, one BLA.al AAV-RFP injection encroached into the BLA.am and BLAp (Supplementary Fig. 4a). Consequently, although the BLA.al AAV-RFP injection largely represents BLA.al projections, it also shows output more specific to BLA.am and BLAp. Similarly, the BLA.al FG injection spread into the LA and BLA.am showing labeling patterns of mostly BLA.al, but also of BLA.am and LA. Tracer spread is expected given the small size of the domains and their close proximity to one another. In fact, infusion spread across ROIs is a valid and pervasive concern for all neuroanatomical studies. Several measures were taken to mitigate this. First, injection sites that were mostly confined to a single domain were selected for analysis so that the label would primarily represent connections of the target domain both in terms of presence and intensity of label. This strategy, combined with the grouping of the three domains for community detection analysis, which assigned grids to injection sites with the greatest pixel intensity value, helped to visualize labeling originating most likely from the target domain. Second, each of the injections was repeated in at least two cases for verification purposes. Third, only tracing data that was validated is reported. Anterograde labels were validated with retrograde tracers, while retrograde labels were validated with anterograde tracers. In the aforementioned BLA.al FG injection, back-labeled cells were observed in rostral ACAd. However, an AAV injection in the ACAd primarily labeled the BLA.am, suggesting that the ACAd labels were from tracer spread into the BLA.am (Supplementary Fig. 4d). In the case of the BLA.al AAV-RFP injection, the MDm and PL were labeled. However, retrograde injections placed in these two regions showed that the MDm label most likely originated from BLAp, while the PL label most likely resulted from tracer spread into the BLA.am (Supplementary Fig. 4e). Further, the PL label from the BLA.al was most evident in the analysis in which this domain was paired with the BLAp. However, once the BLA.al was grouped with the BLA.am and BLA.ac for community detection analysis, projections to the PL got assigned to injection sites in the BLA.am or BLA.ac since injections in these two domains resulted in far denser labels in the PL than the BLA.al injection (Supplementary Fig. 4e).

### BLA injection site analysis

BLA injection sites were rescanned under lower exposure parameters to acquire a more accurate assessment of their size and location. Sections containing the injections for each case were re-registered to ARA levels that best corresponded to the target BLA complex structures. For injections that spanned more than a single section, each section was registered and analyzed. An injection site annotation algorithm (detailed below) was run on the sections to identify injection location. The annotation was run atop a custom ARA atlas that contained the manually delineated boundaries of the BLAa domains (Supplementary Fig. 4a, c).

### Injection site annotation

Correct annotation of the spatial location of injection sites is critical in interpreting neuroanatomical data. In both anterograde and retrograde tracing experiments, the injection sites are typically surrounded by irregularly shaped, very high intensity background pixels. These high intensity background regions yield little useful connectivity information and tend to skew the overall connectivity quantification results as well as interfering with injection site annotation. To quantify the injection sites robustly and consistently, we employ a combination of multi-scale wavelet decomposition, non-linear adaptive intensity adjustment and maximally stable external region (MSER) detection.

Wavelet decomposition encodes both frequency and spatial information of the input data by successive application of high and low pass filters. Given a 2D image f(x, y), its discrete wavelet decomposition of level *L* is as following:

1 $${\rm{f}}(x,\,y)=	\mathop{\sum} _{i}\mathop{\sum} _{j}{C}_{l,\,ij}^{LOW}\cdot {\varPhi }_{{\rm{L}},{\rm{ij}}}(x,\,y)+\mathop{\sum} _{l}\mathop{\sum} _{i}\mathop{\sum} _{j}{C}_{l,\,ij}^{H}\cdot {\varPsi }_{{\rm{l}},{\rm{ij}}}^{{\rm{H}}}(x,\,y)\\ 	+\mathop{\sum} _{l}\mathop{\sum} _{i}\mathop{\sum} _{j}{C}_{l,\,ij}^{V}\cdot {\varPsi }_{{\rm{l}},{\rm{ij}}}^{{\rm{V}}}(x,\,y)+\mathop{\sum} _{l}\mathop{\sum} _{i}\mathop{\sum} _{j}{C}_{l,\,ij}^{D}\cdot {\varPsi }_{{\rm{l}},{\rm{ij}}}^{{\rm{D}}}(x,\,y)$$

Where ${{\bf{C}}}_{{\bf{l}}}^{{\bf{LOW}}}$ is the level $l$ coefficients for the low pass band, and ${{\bf{C}}}_{{\bf{l}}}^{{\bf{H}}},{{\bf{C}}}_{{\bf{l}}}^{{\bf{V}}},{{\bf{C}}}_{{\bf{l}}}^{{\bf{D}}}$ are the level $l$ coefficients for horizontal, vertical and diagonal detail bands (Φ_l_ and Ψ_l_ are the scaling and wavelet functions). For a brain section image f_inj_(*x*, *y*) containing an injection site, we decompose the image into 5 levels and remove the details from levels 1, 2 and 3. At levels 4 and 5, diagonal detail coefficients with large magnitudes are amplified, while horizontal and vertical coefficients with small magnitudes are dampened. Level 5 low pass band coefficients are also dampened and further smoothened with a gaussian kernel. The reverse wavelet transformation of the modified coefficients $\widetilde{{{\bf{C}}}_{{\bf{L}}}^{{\bf{LOW}}}},$ $\widetilde{{{\bf{C}}}_{{\bf{l}}}^{{\bf{H}}}},\widetilde{{{\bf{C}}}_{{\bf{l}}}^{{\bf{V}}}},\widetilde{{{\bf{C}}}_{{\bf{l}}}^{{\bf{D}}}}$ yields a reconstruction of the image $\widetilde{{\text{f}}_{\text{inj}}(x,y)}$ with much of the background intensity in the injection site removed:

2 $$\widetilde{{{\text{f}}}_{{\rm{inj}}}(x,\,y)}=	\mathop{\sum} _{i}\mathop{\sum} _{j}\widetilde{{C}_{L,\,ij}^{LOW}}\cdot {\varPhi }_{{\rm{L}},{\rm{ij}}}(x,\,y)+\mathop{\sum} _{l}\mathop{\sum} _{i}\mathop{\sum} _{j}\widetilde{{C}_{l,\,ij}^{H}}\cdot {\varPsi }_{{\rm{l}},{\rm{ij}}}^{{\rm{H}}}(x,\,y)\\ 	+\mathop{\sum} _{l}\mathop{\sum} _{i}\mathop{\sum} _{j}\widetilde{{C}_{l,\,ij}^{V}}\cdot {\varPsi }_{{\rm{l}},{\rm{ij}}}^{{\rm{V}}}(x,\,y)+\mathop{\sum} _{l}\mathop{\sum} _{i}\mathop{\sum} _{j}\widetilde{{C}_{l,\,ij}^{D}}\cdot {\varPsi }_{{\rm{l}},{\rm{ij}}}^{{\rm{D}}}(x,\,y)$$

The contrast of the reconstructed image $\widetilde{{\text{f}}_{\text{inj}}(x,y)}$ is enhanced using the local adaptive mapping described in^67^. For a normalized image f(*x*, *y*) ∈ [0,1], an initial intensity mapping *T* is defined as

3 $${\rm{T}}({\rm{f}}(x,\,y),\,p)={\sin }^{2}(\frac{\pi }{2}{\rm{f}}{(x,y)}^{p}),\,p \,> \, 0$$

Using the first order Taylor expansion approximation of ${\sin }(u)$, the mapping is rewritten as

4 $${\rm{T}}({\rm{f}}(x,\,y),\,c)=\frac{{\pi }^{2}}{4}{\rm{f}}{(x,y)}^{c},\,c=2p$$

The mapping argument $c$ is defined as $c={c}_{1}\cdot \frac{{\text{f}}_{\text{g}}\left(x,y\right)+\epsilon }{\left(1-{\text{f}}_{\text{g}}\left(x,y\right)\right)+\epsilon }+{c}_{2}$, where ${\text{f}}_{\text{g}}\left(x,y\right)=\text{f}\left(x,y\right)* \text{g}(x,y)$ denotes the convolution between $\text{f}(x,y)$ and a gaussian kernel $\text{g}(x,y)$, while *c*_*1*_, *c*_*2*_ are user specified values. Locally adaptive contrast enhancement is achieved as following:

5 $$\text{E}\left(x,y\right)=\frac{\text{f}(x,\text{y})}{{\text{f}}_{\text{g}}(x,y)}\text{T}\left(\text{f}\left(x,y\right),c\right)+\frac{\text{f}(x,y)}{{\text{f}}_{\text{g}}(x,y)}\frac{\partial \text{T}\left(\text{f}\left(x,y\right),c\right)}{\partial c}\cdot (\text{f}\left(x,y\right)-{\text{f}}_{\text{g}}(x,y))$$

The contrast enhancement further suppresses background pixels at the injection site. Finally, the injection site is extracted as a blob by MSER from $\text{E}(\widetilde{{\text{f}}_{\text{inj}}(x,y)})$, the result of applying wavelet filtering and adaptive contrast enhancement to the injection site image.

### BLAa boundary demarcation via machine learning

Despite sharing many common input and output pathways, the medial (BLA.am), lateral (BLA.al), and caudal (BLA.ac) parts of BLAa each has discerning connections with different regions of the brain. These domain-specific connections were used to compute the boundaries between BLAa domains.

We first examined a collection of coronal sections containing BLAa to identify a subset of sections *S*, where division specific connections can be observed. Each section *s* ∈ S was then associated with a division label ${y}_{s}\in \text{Y}=\left\{\text{medial},\text{lateral},\text{caudal}\right\}$, and registered to the Allen Reference Atlas (ARA). Due to the large z dimension sampling gap of 50 µm between adjacent ARA levels, the BLAa division boundary is computed individually for each ARA level. For any given level $l$, let S^l^ be the subset of sections in $\text{S}$ that best matches with ARA at level $l$. Tracer signal foreground pixels were segmented for each section in S^l^ to obtain a set of coordinates ${{\rm{D}}}_{{\rm{s}}}^{{\rm{l}}}=\{({x}_{s11,}{x}_{s12}),({x}_{s21,}{x}_{s22}),\ldots ({x}_{sn1,}{x}_{sn2})\}$. All $({x}_{{si}1,}{x}_{{si}2})$ pairs inherit the section division label ${y}_{s}$. We used ${{\rm{D}}}^{{\rm{l}}}={\cup }_{{\rm{i}}=1}^{{\rm{card}}({{\rm{S}}}^{{\rm{l}}})}\,{{\rm{D}}}_{{\rm{s}}}^{{\rm{l}}}$ and its corresponding division labels y^l^ to train an ensemble of RBF kernel support vector machines (SVM). The ensemble then determines BLAa subdivisions by assigning a division label to all spatial locations within BLAa.

A SVM classifier solves the following optimization problem:

6 $$\mathop{\min }\limits_{{\bf{w}},b,\zeta }\frac{1}{2}{{\bf{w}}}^{{\rm{T}}}{\bf{w}}+C\mathop{\sum }\limits_{i=1}^{n}{\zeta }_{i}$$

$${\rm{s}}.{\rm{t}}.\;{y}_{i}\left({{\bf{w}}}^{{\rm{T}}}{\varphi }_{\gamma }({{\bf{x}}}_{{\rm{i}}})+b\right)\ge 1-{\zeta }_{i}$$

$${\zeta }_{i}\ge 0$$

The classifier has two parameters: the soft margin parameter $C$ and the kernel (radial basis function φ) parameter *γ*. A total of 64 classifiers were trained with *C* and *γ* take values on a grid of powers of 2. For a classifier SVM_i_, a model accuracy ${a}_{i}$ is computed as the average accuracy from 10 threefold stratified cross-validation training and testing. Given any point **x** = (*x*_1_, *x*_2_) within BLAa at level *l*, denote the division prediction by ${\text{SVM}}_{\text{i}}$ as ${\text{y}}_{\text{i}}({\bf{x}})$. The BLAa division of ${\bf{x}}$ at level $l$ is determined by a weighted averaging ensemble:

7 $${\text{E}}^{l}({\bf{x}})=\mathop{\text{argmax}}\limits_{y\in \text{Y}}\mathop{\sum} _{i}{\text{a}}_{\text{i}}\cdot \text{I}({\text{y}}_{\text{i}}\left({\bf{x}}\right)=y)$$

Dense classification across BLAa with *E*^*l*^ produces the optimal BLAa divisions based on division specific anatomical pathways.

### Statistical analysis of BLAa projections

Quantitative comparisons of projection labels from BLAa domains were performed to supplement and validate the qualitative analysis. Repeated anterograde tracer injections were made in each BLAa domain (BLA.am *n* = 8; BLA.al *n* = 6; BLA.ac *n* = 7). Analysis was limited to 12 ARA levels (versus 32 levels) and to select ROIs (ACA, PL, ILA, DP, TTd, MOs, CA1, CA3, PAR, SUBd, SUBv). All the data was processed through Connection Lens (Fig. 2a) and the annotated data was subjected to statistical analyses.

Two-sided pairwise Wilcoxon rank sum tests were performed, and the parameters that survived FDR correction for multiple testing with *p* values <0.05 are reported and visualized in whisker plots (Supplementary Fig. 5b–m). The quantitative results agreed with the qualitative analyses. Significant differences in projection densities were found in PL2 (BLA.am vs BLA.al, W = 47, *p* = 0.001, [0.006, 0.003]; BLA.am vs BLA.ac, W = 4, *p* = 0.003, [−0.006, −0.001]; BLA.al vs BLA.ac, W = 0, *p* = 0.001, [−0.008, −0.002]), ILA 37/39 (BLA.am vs. BLA.al, W = 7, *p* = 0.02, [−0.003, −0.00005]; BLA.am vs BLA.ac, W = 4, *p* = 0.003, [−0.002, −0.0005]), ILA 41/45 (BLA.am vs BLA.ac, W = 0, *p* = 0.0003, [−0.0006, −0.00015]; BLA.al vs BLA.ac, W = 2, *p* = 0.004, [−0.0006, −0.0005]), CA3 (BLA.am vs BLA.al, W = 1, *p* = 0.0025, [−0.00002, −0.00001]; BLA.am vs BLA.ac, W = 0, *p* = 0.001, [−0.001, −0.0004]; BLA.al vs BLA.ac, W = 0, *p* = 0.001, [−0.001, −0.0003]), ACAd (BLA.am vs BLA.al, W = 46, *p* = 0.002, [0.0003, 0.002]; BLA.am vs BLA.ac, W = 47, *p* = 0.02, [0.0001, 0.002]), MOs (BLA.am vs BLA.al, W = 44, *p* = 0.007, [0.0006, 0.003]; BLA.am vs BLA.ac, W = 55, *p* = 0.0006, [0.001, 0.004]; BLA.al vs BLA.ac, W = 37, *p* = 0.02, [0.0003, 0.001]), DP (BLA.am vs BLA.al, W = 0, *p* = 0.0006, [−0.001, −0.0003]; BLA.al vs BLA.ac, W = 42, *p* = 0.001, [0.0004, 0.001]), TTd (BLA.am vs BLA.al, W = 5, *p* = 0.012, [−0.0006, −0.0001]; BLA.al vs BLA.ac, W = 42, *p* = 0.001, [0.0001, 0.0009]), CA1 (BLA.am vs BLA.al, W = 8, *p* = 0.04, [−0.0004, −4.41e^−06^]; BLA.am vs BLA.ac, W = 0, *p* = 0.0003, [−0.008, −0.002]; BLA.al vs BLA.ac, W = 0, *p* = 0.001, [−0.007, −0.002]), PAR (BLA.am vs BLA.ac, W = 0, *p* = 0.003, [−0.006, −0.002]; BLA.al vs BLA.ac, W = 0, *p* = 0.001, [−0.006, −0.002]), SUBv (BLA.am vs BLA.ac, W = 0, *p* = 0.0003, [−0.007, −0.003]; BLA.al vs BLA.ac, W = 0, *p* = 0.001, [−0.007, −0.002]), and SUBd (BLA.am vs BLA.ac, W = 4, *p* = 0.003, [−0.0003, −4.7e^−05^]; BLA.al vs BLA.ac, W = 6, *p* = 0.03, [−4.2e^−04^, −5.6e^−05^]).

### Generation of 2D hierarchical clustering of anterograde projection data

To assess injection site reproducibility, repeated anterograde tracer injections in BLA.am (*n* = 8), BLA.al (*n* = 6), and BLA.ac (*n* = 7) were used. For each case, the number of segmented anterograde projection pixels to each target region were compiled for each section. The pixel counts for regions spanning multiple ARA levels were summed for each case to form a projection vector with dimension equal to number of regions delineated in ARA. We used the L1 normalized projection vectors, which represent the fraction of projection to each region as input data for hierarchical clustering. The cosine distance metric was used during linkage. Row labels show the injection site and tracer used for each case. Column labels show name of the target region. Only regions receiving rich projections are shown in the plot for legibility. In our own analysis, we included all target regions and obtained an identical row clustering outcome. We used scikit-learn for SVM implementation, and scipy and seaborn to generate and visualize 2D hierarchical clustering results.

### Community detection

We performed community detection (modularity maximization) on both grid and ROI annotated data. As a first step, the overlap annotation per each group was aggregated into a single matrix. We set a minimum threshold value of 0.0045 for labeled pixels (anterograde) and 8 for labeled cells (retrograde), and removed entries that did not meet the threshold. These minimum values were set to exclude potential false positives, but also to exclude extremely light connections that would be challenging to interpret (e.g., 2 labeled cells or a few labeled fibers in an ROI). Once the aggregated matrix was constructed, we further normalized so that the total labeling across each injection site (typically close in the first place) was adjusted to equal with the injection site featuring maximum total labeling. On this normalized matrix we applied the Louvain^68^ algorithm at a single scale (gamma 1.0). As the result of this greedy algorithm is non-deterministic, we performed 100 separate executions, and subsequently calculated a consensus community structure^69^ to characterize the 100 executions as a single result.

### Connectivity matrices, community color coding and data visualization

For matrix visualization, we applied the grid communities algorithm (bctpy; https://github.com/aestrivex/bctpy, https://sites.google.com/site/bctnet/) to modularize ROIs based on community assignment, as well as prioritize connections along the diagonal. Such a visualization provides a high level overview of the connectivity (Fig. 3h, i; Fig. 4f, g).

Community coloring provides more detail (i.e., drill down from the matrix overview) by visually encoding segmentation by community, and ultimately, injection site (Fig. 2a, anterograde and retrograde maps). To carry out this process, we developed software to programmatically march through each segmented pixel per each image. Using either the grid location or ROI name, the algorithm looked up the corresponding community assigned during the consensus community step. Using a table containing a color assigned (by the authors) to each injection site, the algorithm retrieved the injection site associated with the community, and colored the pixel with the corresponding injection site color value. A subsequent step took advantage of the fact that each pixel was assigned to only a single community by aggregating all colorized images corresponding to a given atlas level into a single representative image.

### Proportional stacked bar charts

It is challenging to represent grid-based overlap connectivity in matrix form. A naive visualization labels row and columns by grid location, which is hardly informative. One solution is to use color coding to identify community labels^25^, but comprehending that visualization requires comparisons with at least one other image to qualify the anatomical region that the labeling occurs in. Such a matrix also does not identify where the labeling occurs along the rostral to caudal axis, for example.

To overcome these limitations, we visualized the connectivity data using a proportional, stacked bar chart approach. The chart categorizes data by atlas level. The community coloring is retained, but region labels identify the two ROIs that the labeling occurs most within. A focused version of this visualization isolates the analysis to a specific set of ROIs (e.g., olfactory tubercle, nucleus accumbens). Another view splits the ROIs into quadrants (e.g., intermediate CP, CPi) (and other combinations of medial-lateral, dorsal-ventral) to resolve termination at higher resolution.

### 3D tissue processing

To assess whether projection neurons located in BLA.am, BLA.al, and BLA.ac were morphologically distinct, representative neurons in each of the domains were labeled via a RVΔG injection in the caudal dorsomedial caudoputamen (for BLA.am), caudal ventral caudoputamen (for BLA.al), and in the medial accumbens (for BLA.ac) (Fig. 1d). One week was allowed for tracer transport following injections, after which the animals were perfused. A 3D tissue processing workflow was followed for implementation of the SHIELD clearing protocol^70^.

Mice were transcardially perfused with ice-cold saline and SHIELD perfusion solution. The brains were extracted and incubated in the SHIELD perfusion solution at 4 °C for 48 h. The SHIELD perfusion solution was replaced with the SHIELD OFF solution and tissues were incubated at 4 °C for 24 h. The SHIELD OFF solution was replaced with the SHIELD ON solution and the tissues were incubated at 37 °C for 24 h. The whole brain was cut into 250- (for BLA.am and BLA.al) or 400 (for BLA.ac) µm sections and were cleared in the SDS buffer at 37 °C for 72 h. The sections were then washed three times with KPBS and incubated in KPBS at 4 °C for 24 h.

### 3D imaging protocol

Sections were mounted and coverslipped onto 25 × 75 × 1mm glass slides with an index matching solution 100% (EasyIndex, LifeCanvas Technologies, #EI-Z1001). Sections were imaged with a high speed spinning disk confocal microscope (Andor Dragonfly 202 Imaging System, Andor an Oxford Instruments Company, CR-DFLY-202-2540). 10x magnification (NA 0.40, Olympus, UPLXAPO10X) was used to acquire an overview after which 30x magnification (NA 1.05, Olympus, UPLSAPO30xSIR) was used to image through the BLA ipsilateral to the injection site at 1 µm z steps. The BLA contralateral to the injection was also imaged for the BLA.ac case.

### 3D reconstructions, visualization, and analysis of neuronal morphology

Manual reconstruction of the neurons was performed using Aivia (version.8.5, DRVision) (Fig. 9d), and geometric processing of neuron models was performed using the Quantitative Imaging Toolkit (QIT)^71^ (also available at http://cabeen.io/qitwiki). Although CPc.dm projecting BLAa neurons primarily are located in BLA.am, some are also located in BLA.al and BLA.ac. As such, rabies injections in the CPc.dm label mostly BLA.am, but also some BLA.al and BLA.ac neurons. To ensure the traced neurons in fact solely originated from their respective domains, for all cases, labeled cells visually grouped in the center of each domain (X-Y axes) were traced. Neurons located within the BLA.am were identified for inclusion in the analysis, but those close to the BLA.al or BLA.ac border were excluded. Similarly, neurons within BLA.al and BLA.ac were identified for inclusion, but those close to the border of the BLAp were omitted. In addition, domain-specific neurons in deeper parts of the tissue (Z axis) farthest away from the edges of the sectioned tissue were selected for reconstruction. To assist in the accurate selection of neurons, maximum intensity projections of 25 µm were created that aided in the identification of the BLAa domain boundaries. In total, 8 neurons were traced for the BLA.am, 9 for the BLA.al, and 6 for the BLA.ac.

Reconstructions of individual neurons posed a challenge due to dense labeling in the BLA.am and BLA.al regions (Fig. 1d). All labeled neurons in the BLA.ac contralateral to the injection site were also traced to show the details of dendritic morphology that could be captured and reconstructed with RVΔG injections (*n* = 3; Fig. 9c) (Supplementary Movies 4–5). To mitigate the issue of dense labeling, but also to account for differences in slice thickness (250 µm for BLA.am and BLA.al and 400 µm for BLA.ac), we restricted our morphological analysis to neurites that were sufficiently close to the soma. We accomplished this by applying the NeuronTransform module in QIT to trim the contiguous portion of neurites that measured farther than 300 nm away from the center of the soma using the Euclidean distance (Supplementary Fig. 1g). With this distance, we were confident in our ability to accurately trace dendrites, regardless of neuron density. Due to the anisotropic dimensions of the voxels and spatial undersampling relative to the curvature of the dendrite, we applied a local regression filter to address the aliasing artifact and to regularize dendritic tortuosity. Specifically, the NeuronFilter module in QIT was used to apply a locally weighted scatter-plot smoother (LOESS), which is a low bias approach that makes minimal assumptions (Supplementary Fig. 1h)^72^. LOESS was implemented using a locally quadratic function with the five nearest neighbor neuron vertices before and after each point of estimation endpoints and bifurcation points were excluded from filtering.

Importantly, CP-projecting BLA.am/BLA.al neurons and ACB-projecting BLA.ac neurons were selected as representative domain specific BLA→striatum neurons and are not representative of the entire population of neurons within the 3 domains. It is possible that BLA→striatum neurons display morphological features that differ from BLAa→MPF or BLAa→AI neurons.

### Statistical analysis of morphometrics

To obtain an overall view of the dendritic morphology of BLA projection neurons located in the BLA.am, BLA.al, and BLA.ac, we applied the classic and modified Sholl analysis using Fiji ImageJ and L-Measure, respectively. The resulting classic Sholl scatter plot shows dissimilarity between groups, especially within 100–200 nm radius from the cell body (Supplementary Fig. 1i). A three-dimensional Sholl-like analysis showed that in BLA.ac neurons, the distribution of dendritic surface area was more peaked toward the median of the relative path distance from the soma as opposed to the more uniformly distributed dendritic surface of BLA.am and BLA.al neurons (Fig. 9a). A visual comparison shows that the BLA.ac neurons are qualitatively more distinct from those in the BLA.am and BLA.al.

Quantitative morphological parameters depicting the somas and dendrites were obtained from Aivia and statistical analyses were performed using the R computing environment (RStudio Version 1.1.463). Standard measurements representing cell body morphology included volume, sphericity, spherical diameter, X, Y, Z, and Euclidean skewness, cell body height, width, depth, and surface to volume ratio. Measurements of dendritic morphology included number of primary dendrites, branches, bifurcations, terminal tips, nodes, fractal dimension, tortuosity, local bifurcation angle, remote bifurcation angle, partition asymmetry, tips to dendrite ratio, branch generation, and Rall’s ratio.

Overall, the results suggest that neurons located in each BLAa domain are morphologically distinct. Using all measured morphological parameters, principal component analysis (PCA) was run to reduce the dimensionality and create a 3D scatterplot. The PCA shows the segregation of BLAa domain specific neurons based on the measured features (Fig. 9b).

Two-sided pairwise Wilcoxon rank sum tests were performed, and the parameters that survived FDR correction for multiple testing with *p* values <0.05 are reported. The significant group differences are presented with whisker plots (Fig. 9f), and the degree of their significance is visualized in a matrix plot (Fig. 9e). Although significant differences in several morphological features were detected across all pairwise comparisons, generally, neurons in the BLA.am and BLA.al significantly differed from those in the BLA.ac on a greater number of morphological features. This is also evidenced by the dendrogram on top of the matrix that shows the hierarchical clustering based on group similarity (Fig. 9e). Specifically, BLA.am neurons differed from BLA.al neurons in somatic features like cell body height (W = 59.5, *p* = 0.04, [0.22, 4.18]) and in dendritic morphology like fractal dimension (W = 69, *p* = 0.001, [0.006, 0.017]) and tortuosity (W = 67, *p* = 0.0023, [0.02, 0.07]).

Neurons in BLA.am and BLA.al significantly differed from those in BLA.ac on cell body features like cell width (BLA.am vs BLA.ac, W = 43, *p* = 0.038, [1.76, 8.36]; BLA.al vs. BLA.ac, W = 46. *p* = 0.043. [0.44, 5.94]), skewness along the X dimension (BLA.am vs BLA.ac, W = 44, p = 0.012, [6.5, 32.0]; BLA.al vs. BLA.ac, W = 49, *p* = 0.012, [6.5,33.5]), and sphericity (BLA.am vs BLA.ac, W = 47, *p* = 0.004, [0.01, 0.036]; BLA.al vs BLA.ac, W = 50, *p* = 0.0072, [0.012, 0.038]). They also differed in a number of dendritic features like the number of bifurcations (BLA.am vs. BLA.ac, W = 6.5, *p* = 0.041, [−12.0 −0.99]; BLA.al vs. BLA.ac, W = 4.5, *p* = 0.027, [−10.0, −1.99]), number of branch generations (BLA.am vs BLA.ac, W = 2, *p* = 0.004, [−0.95, −0.19]; BLA.al vs BLA.ac, W = 1, *p* = 0.004, [−0.1, −0.35]), and tips to dendrite ratio (BLA.am vs. BLA.ac, W = 4, *p* = 0.018, [−1.86, −0.4]; BLA.al vs BLA.ac, W = 2.5, *p* = 0.014, [−1.8, −0.47]).

BLA.am neurons differed from those in BLA.ac on additional features including cell height (W = 46, *p* = 0.016, [1.98, 5.72]), fractal dimension (W = 48, *p* = 0.001, [0.009, 0.02]), tortuosity (W = 48, *p* = 0.002, [0.02, 0.08]), soma volume (W = 45, *p* = 0.014, [1136.7, 3795.5]), surface to volume ratio (W = 1, *p* = 0.004, [−0.06, −0.001]), and spherical diameter (W = 45, *p* = 0.014, [1.37, 4.1]).

Domain-specific BLAa neurons did not significantly differ from one another in the number of primary dendrites, number of nodes, number of branches, terminal tips, local bifurcation angle, remote bifurcation angle, partition asymmetry, Rall’s ratio, depth, Y, Z, or Euclidean skewness.

In a final analysis, we characterized our data using an alternative tool that uses topological data analysis that retains potentially more morphological information. Specifically, we used the persistence-based neuronal feature vectorization framework^73^ to summarize pairwise distances between neurons^74^. Our experiments used code to first compute persistence diagrams using NeuronTools (https://github.com/Nevermore520/NeuronTools) and then computed inter-neuron distances using the Wasserstein metric (https://bitbucket.org/grey_narn/geom_matching/src). The results were visualized in a matrix plot created using R (Fig. 9g, h). Similar to the PCA analysis, the data showed that groups of projections neurons in BLA.am, BLA.al, and BLA.ac all differed from one another, but the greatest differences were between BLA.am/BLA.al neurons compared to those in BLA.ac (Fig. 9h).

### Slice recordings

Three weeks following the viral injections of AAV-hSyn-ChR_2_-YFP (UPenn Vector Core) and red retrograde microbeads (Lumafluor Inc.), animals were decapitated following isoflurane anesthesia and the brain was rapidly removed and immersed in an ice-cold dissection buffer (composition: 60 mM NaCl, 3 mM KCl, 1.25 mM NaH_2_PO_4_, 25 mM NaHCO_3_, 115 mM sucrose, 10 mM glucose, 7 mM MgCl_2_, 0.5 mM CaCl_2_; saturated with 95% O2 and 5% CO_2_; pH = 7.4). Coronal slices at 350 μm thickness were sectioned by a vibrating microtome (Leica VT1000s), and recovered for 30 min in a submersion chamber filled with warmed (35 °C) ACSF (composition:119 mM NaCl, 26.2 mM NaHCO_3_, 11 mM glucose, 2.5 mM KCl, 2 mM CaCl_2_, 2 mM MgCl_2_, and 1.2 NaH_2_PO_4_, 2 mM Sodium Pyruvate, 0.5 mM VC). BLA neurons (labeled with fluorescent microbeads) surrounded by EYFP+ fibers were visualized under a fluorescence microscope (Olympus BX51 WI). Patch pipettes (~4–5 MΩ resistance) filled with a cesium-based internal solution (composition: 125 mM cesium gluconate, 5 mM TEA-Cl, 2 mM NaCl, 2 mM CsCl, 10 mM HEPES, 10 mM EGTA, 4 mM ATP, 0.3 mM GTP, and 10 mM phosphocreatine; pH = 7.25; 290 mOsm) were used for whole-cell recordings. Signals were recorded with an Axopatch 700B amplifier (Molecular Devices) under voltage clamp mode at a holding voltage of –70 mV for excitatory currents, filtered at 2 kHz and sampled at 10 kHz. Tetrodotoxin (TTX, 1 μM) and 4-aminopyridine (4-AP, 1 mM) were added to the external solution for recording monosynaptic responses to blue light stimulation (5 ms pulse, 3 mW power, 10–30 trials).

### Reporting summary

Further information on research design is available in the Nature Research Reporting Summary linked to this article.

## Supplementary information

Supplementary information

Description of Additional Supplementary Files

Supplementary Movie 1

Supplementary Movie 2

Supplementary Movie 3

Supplementary Movie 4

Supplementary Movie 5

Reporting Summary

## Data Availability

Raw data for amygdala cases used in the paper can be accessed through our Mouse Connectome Project website (https://mouseconnectomeproject.github.io/amygdalar/iconnectome). The color-coded visualized output of the community assignments for all injections are available (https://mouseconnectomeproject.github.io/amygdalar/), as well as the BLA global wiring diagram (https://mouseconnectomeproject.github.io/amygdalar/wiringdiagram). Reconstructions of BLAa neurons are cataloged in NeuroMorpho.org. SWG files and associated metadata for neuron reconstructions can be downloaded here: neuromorpho.org/dableFiles/dong/Supplementary/Dong2020BLA.zip. Source data are provided with this paper.

## Source data

Source data

## Code availability

An in-house software was used to process all image data presented in the manuscript. Connection Lens was specifically applied to register (warp), threshold (segment), and annotate the labels in all image data. This software has not been publicly released yet. However, custom code developed for data analysis is available at https://github.com/ibowman/BLA. For the Louvain algorithm implementation, the Brain Connectivity Toolbox (BCT) was employed, which is freely available at https://sites.google.com/site/bctnet/. The grid communities algorithm for matrix visualizations are available here at https://github.com/aestrivex/bctpy, https://sites.google.com/site/bctnet/. Geometric processing of neuron models was performed using the Quantitative Imaging Toolkit (QIT), available at http://cabeen.io/qitwiki. We also computed persistence diagrams using NeuronTools (https://github.com/Nevermore520/NeuronTools) and then computed inter-neuron distances using the Wasserstein metric (https://bitbucket.org/grey_narn/geom_matching/src). We used scikit-learn for SVM implementation, and scipy and seaborn to generate and visualize 2D hierarchical clustering results. Finally, a dynamic image carousel web application which features brain-wide pathway reconstructions of tracer-labeled connections from the amygdala was developed. Users can navigate colorful projections of both anterograde and retrograde labels mapped atop the standard ARA atlas. This application is currently hosted at GitHub web portal (https://mouseconnectomeproject.github.io/amygdalar/) and the code repository can be found at https://github.com/mouseconnectomeproject/amygdalar.
