# Supplementary material for: Connectivity characterization of the mouse basolateral amygdalar complex
Source: Nat Commun. 2021 May 17;12:2859. doi: 10.1038/s41467-021-22915-5 (PMC8129205; doi:10.1038/s41467-021-22915-5)
Supplement: Supplementary file 1 — Supplementary information [file 41467_2021_22915_MOESM1_ESM.pdf]

Supplementary information for

Connectivity characterization of the mouse basolateral amygdalar complex

Houri Hintiryan, Ian Bowman, David L. Johnson, Laura Korobkova, Muye Zhu, Neda Khanjani, Lin Gou, Lei Gao, Seita Yamashita, Michael S. Bienkowski, Luis Garcia, Nicholas N. Foster, Nora L. Benavidez, Monica Y. Song, Darrick Lo, Kaelan R. Cotter, Marlene Becerra, Sarvia Aquino, Chunru Cao, Ryan P. Cabeen, Jim Stanis, Marina Fayzullina, Sarah A. Ustrell, Tyler Boesen, Amanda J. Tugangui, Zheng-Gang Zhang, Bo Peng, Michael S. Fanselow, Peyman Golshani, Joel D. Hahn, Ian R. Wickersham, Giorgio A. Ascoli, Li I. Zhang, Hong-Wei Dong

Corresponding authors:

Hong-Wei Dong: HongweiD@mednet.ucla.edu

Houri Hintiryan: HHintiryan@mednet.ucla.edu

Supplementary Figures 1-16

Supplementary Fig. 1

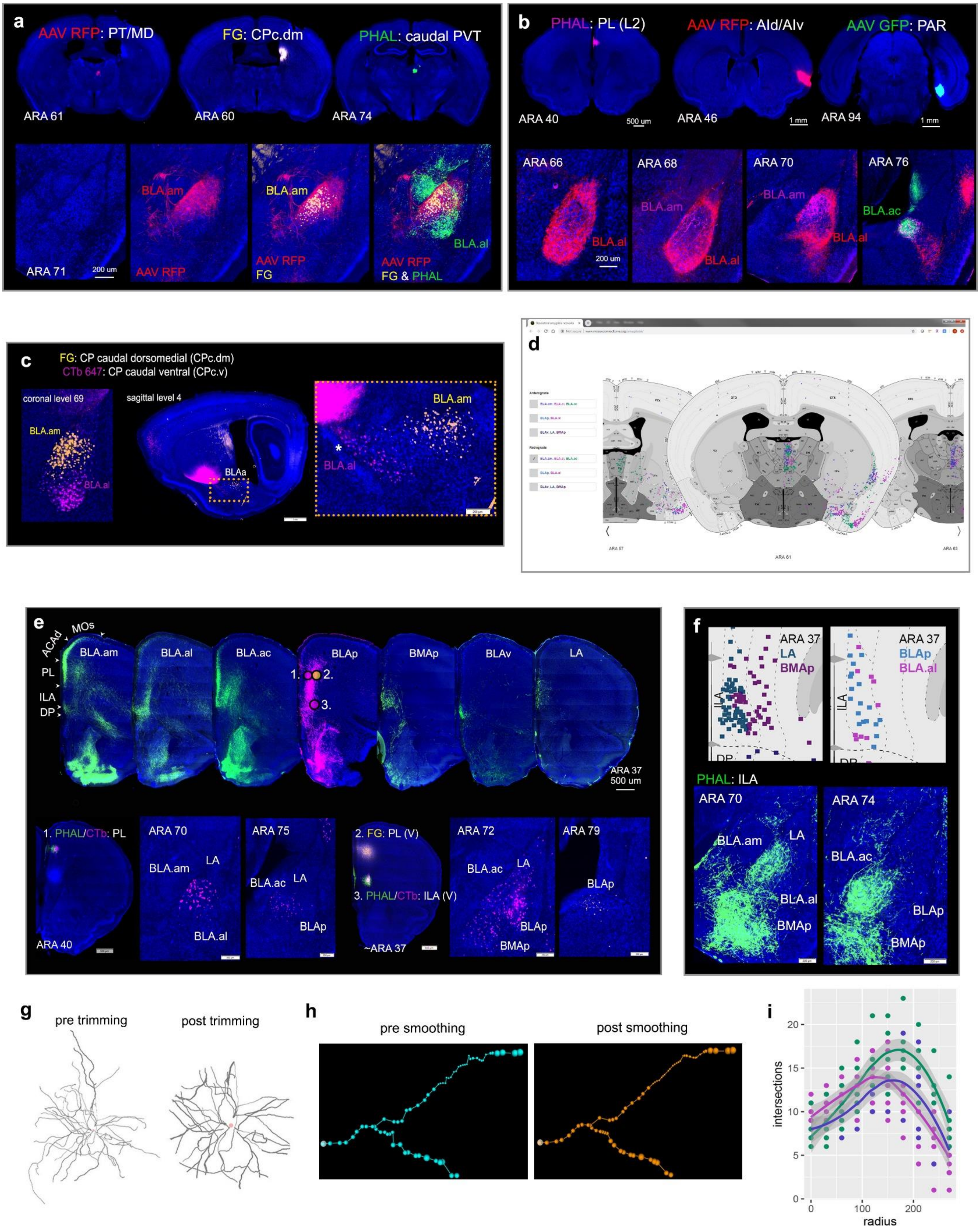

Supplementary Fig. 1: Distinct connections and morphology of BLAa neurons

a. Validation of distinct BLA.am and BLA.al connections. Anterograde tracers AAV RFP and PHAL in thalamic PT/MD and caudal PVT, label either BLA.am or BLA.al, respectively. In the same animal, a FG injection in CP caudal dorsomedial (CPc.dm) labels BLA.am projection neurons, which overlap with AAV RFP labeled fibers from PT. **b.** Triple anterograde tracer injections across cortical regions label distinct BLAa domains. PHAL in PL (pink) labels fibers in BLA.am, while AAV RFP in AId/AIv (red) labels BLA.al, and AAV GFP in PAR (green) labels fibers in BLA.ac. **c.** Retrograde tracers FG (yellow) and CTb (pink) injected in either the dorsomedial (CPc.dm) or ventral (CPc.v) part of caudal caudoputamen distinctly label BLA.am and BLA.al projection neurons, respectively, in both coronal (left) and sagittal (right) views. Inset shows magnified version of boxed region on the sagittal section. **d.** Snapshot of the application created to view anterograde and retrograde maps, all freely available online (https://mouseconnectomeproject.github.io/amygdalar/). **e.** Top row shows projections to medial prefrontal cortex from neurons in BLA.am, BLA.al, BLA.ac, BLAp, BMAp, BLAv, and LA. Connections from BLAp to deeper layers of ILA and PL were validated with retrograde tracer injections placed in 1. superficial layers of PL(II/III) (CTb: pink), 2. deeper layers of PL(V/VI) (FG: yellow), and 3. deeper layers of ILA(V/VI) (CTb: pink). Bottom row: CTb injection in PL(II/III) validates BLA.am/BLA.ac→PL(II/III) connection. This injection weakly labels BLAp neurons. FG in deeper PL(V/VI) and CTb in ILA(V/VI) back-label mostly BLAp neurons confirming BLAp→PL/ILA (deeper layers). LA→ILA was also confirmed. **f.** Validation of strong ILA→LA and ILA(V)→BMAp connections and weak ILA→BLAp and ILA→BLA.al connections. Top row: retrograde maps showing back-labeled ILA neurons following retrograde tracers in LA, BMAp, and BLAp. Bottom row: validation of those connections with an ILA PHAL injection (injection 3 in panel e). PHAL label evident in LA, BMAp, BLA.am, and BLA.ac, but far less in BLA.al and BLAp. **g.** Due to differences in slice thickness for the reconstruction of BLA.am (250 µm), BLA.al (250 µm), and BLA.ac (400 µm) projection neurons, morphological analysis of neurites was restricted by trimming the portions of the dendritic branches that measured farther than 300 nm from the soma. Left shows a reconstructed striatum projecting BLA.ac neuron in its entirety, while the right shows the same neuron with applied trimming. **h.** Due to anisotropic voxels and spatial undersampling relative to the real curvature of the dendrite, a locally weighted scatter-plot smoother (LOESS) was applied to the data. Left shows curvature prior to smoothing, while the left shows post smoothing curvature. **i.** Result of traditional Sholl analysis showing overall view of BLAa projection neuron dendritic morphology. Scatter plot shows that BLA.am and BLA.al neuron dendrites mostly differed from those in BLA.ac within 100-200 nm radius from the cell body, with BLA.ac dendrites showing a greater number of intersections within that range. *Abbreviations: AId: agranular insular area, dorsal part; AIv: agranular insular area, ventral part; ILA: infralimbic cortical area; MD: mediodorsal thalamic nucleus; PAR: parasubiculum; PL: prelimbic cortical area; PT: parataenial thalamic nucleus; PVT: paraventricular thalamic nucleus.*

Supplementary Fig. 2

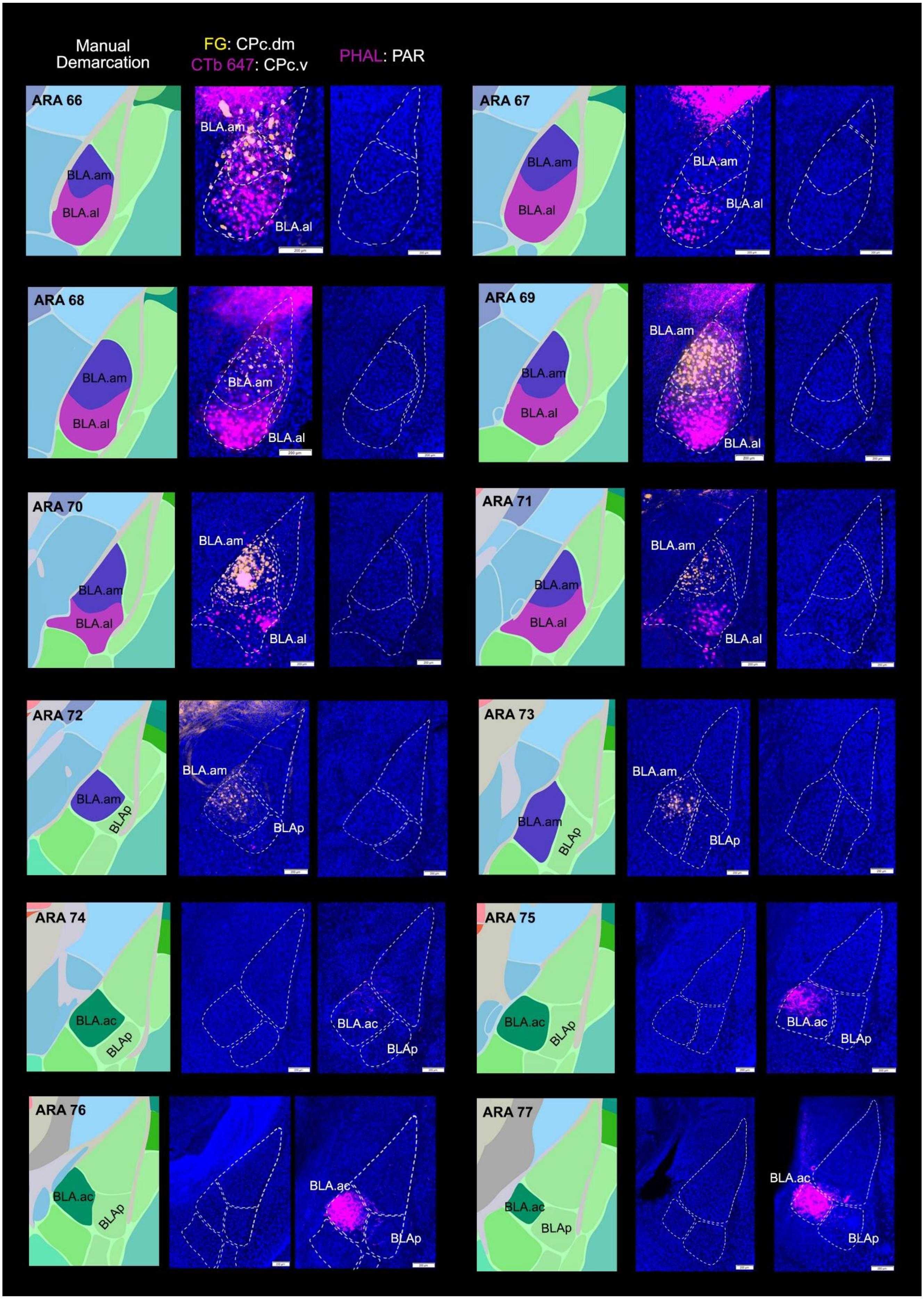

Supplementary Fig. 2: Connectivity-defined delineation of BLA.am, BLA.al, and BLA.ac

For demarcation of BLA.am, BLA.al, and BLA.ac borders, cases with distinct labels within each domain were selected and contiguous sections through the BLAa from ARA level 66 to ARA77 were collected (n=7). Two representative cases are shown: one in which FG and CTb were injected in CP caudal dorsomedial (CPc.dm) and CP caudal ventral (CPc.v), respectively and another in which PHAL was injected in PAR. Note the clear FG-labeled BLA.am projection neurons from ARA 67-73, which disappear at level 74, where PHAL label from the PAR injection begins to appear. CTb labeled BLA.al projection neurons are observed from ARA 66-71, after which they disappear at ARA 72, where BLAp appears. Initially, borders were drawn manually guided by mapped labels, but also by Nissl stains for each case (Supplementary Fig. 3b). The same data was used as a training set for a machine learning algorithm, which produced similar BLAa border delineations (92% agreement) (Supplementary Fig. 3a).

Supplementary Fig. 3

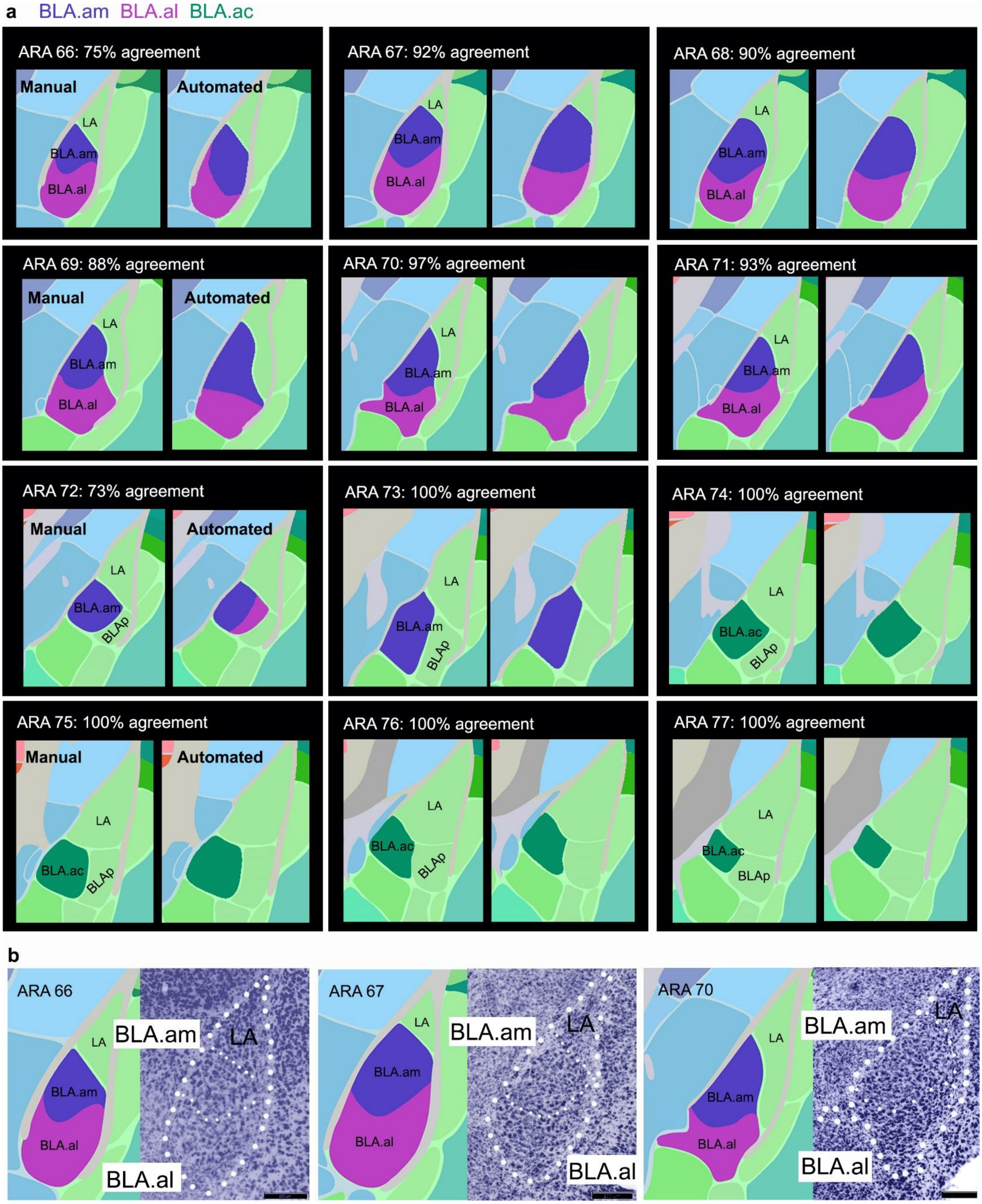

Supplementary Fig. 3: Automated and manual demarcations of BLA.am, BLA.al, and BLA.ac boundaries

a. Output of manual versus automated boundary demarcation for BLA.am, BLA.al, and BLA.ac across ARA levels 66-77. Initially, borders were drawn manually guided by mapped labels and Nissl stains. The same data was used as a training set for a machine learning algorithm for automated BLAa boundary demarcation. On average, across all sections there was a 92% agreement between the manually drawn versus the automatically detected boundaries. The lowest agreements were at ARA 66 and 72 with 75% and 73% agreements, respectively. These percentages are reasonable for both levels given that at more rostral levels of BLAa like ARA 66, segregation of labels was far less clear, hence the exclusion of ARA levels 63-65 despite the presence of BLAa at those levels. ARA 72 is the first section where the BLA.al disappears so it is understandable for the automated method to identify it as a transitioning section in which both BLA.am and some BLA.al are present. **b.** Importantly, the boundary between the BLA.am and BLA.al was assessed by examination of BLA cytoarchitecture. A representative Nissl case showing the BLA.am BLA.al distinction is presented here with delineated boundaries for ARA levels 66-70.

Supplementary Fig. 4

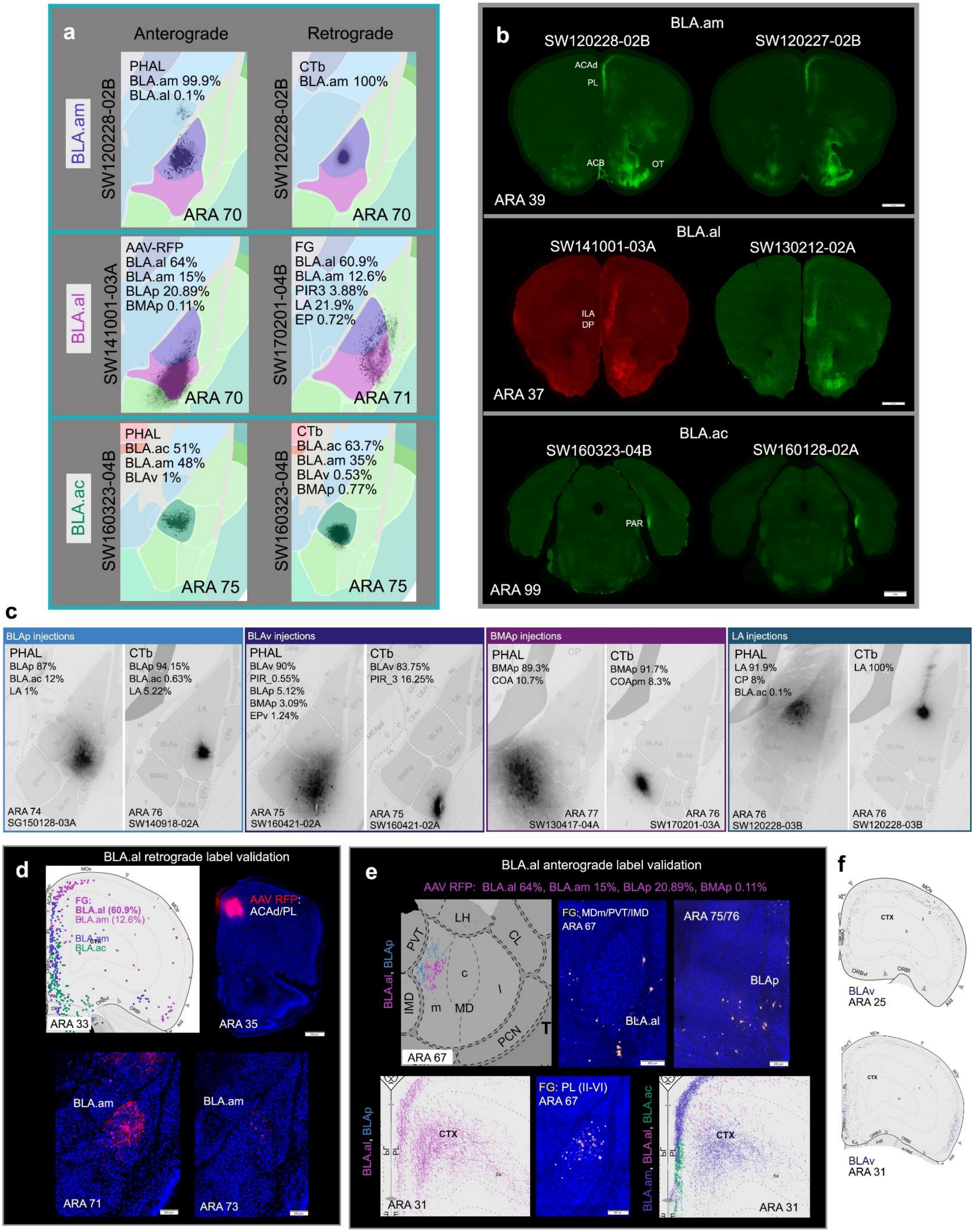

Supplementary Fig. 4: BLA injection sites and label validation

a. Representative images of tracer injections in BLA.am, BLA.al, and BLA.ac. Percentages represent amount of tracer calculated in each ROI. Although a single image is presented, percentages were calculated based on all sections that contained the tracer injection. **b.** Anterograde labeling of main injection cases and validation cases for BLA.am, BLA.al, and BLA.ac showing consistent patterns of labeling. **c.** Representative images and percentages of anterograde and retrograde tracer injections in BLAp, BLAv, BMAp, and LA. **d.** Retrograde label validation using BLA.al as an example. A FG injection primarily in BLA.al, that encroached into BLA.am, shows back-labeled neurons in ACAd (pink cells on retrograde map) suggesting an ACAd→BLA.al connection. An anterograde injection in ACAd shows label only in BLA.am (ACAd→BLA.am), suggesting the back-label in ACAd from the BLA.al FG injection was most likely from leakage into BLA.am (purple cells). *Abbreviations: ACAd: anterior cingulate cortex, dorsal part.* **e.** Additional examples illustrating the importance of cross validation of connections. Top panels: an AAV RFP injection mostly in BLA.al, that encroached into BLAp, shows labeled fibers in MDm (pink fibers on anterograde map) indicating a BLA.al→MDm connection. A FG injection in MDm back-labels only BLAp projection neurons suggesting the FG label from the BLA.al injection originates from BLAp (blue fibers) and not BLA.al (BLAp→MDm). Bottom panels: the same AAV-RFP BLA.al injection also leaked into BLA.am and labeled different layers of PL (pink fibers in anterograde map to the left). A FG injection in PL back-labels mostly BLA.am neurons suggesting the labeled PL fibers from the BLA.al injection was from leakage into BLA.am. Note that the anterograde map on the left is from grouping of BLA.al and BLAp and the one to the right is from grouping BLA.al with BLA.am and BLA.ac. In the latter, PL get assigned to BLA.am/BLA.ac injections rather than to BLA.al due to the stronger BLA.am→PL connections at ARA level 31, validating our method of analysis. *Abbreviations: MDm: mediodorsal thalamic nucleus, medial part; PL: prelimbic cortical area.* **f.** Anterograde maps showing BLAv→MOs (secondary motor cortex) projections.

Supplementary Fig. 5

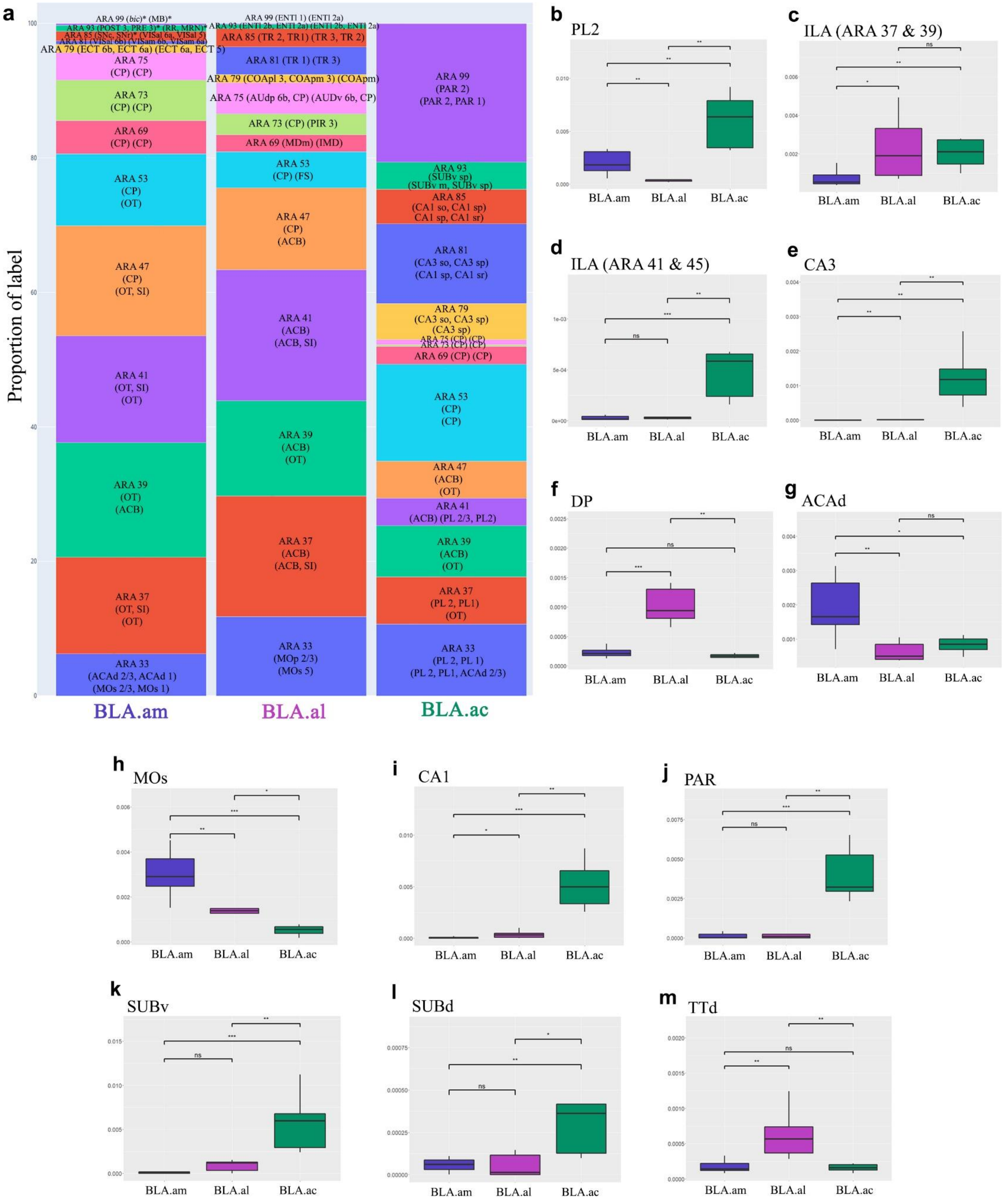

Supplementary Fig. 5: BLAa-specific connections and quantified BLAa anterograde labels

a. Bar chart showing proportion of label from representative BLA.am, BLA.al, and BLA.ac injections (n=1 each) to different ARA levels. ROIs for grids with strongest projections at each ARA level are displayed in parentheses. Each grid unit (square) can include multiples ROI (e.g., ACAd2/3, ACAd 1 for BLA.am ARA 33). ** denotes anterograde projections that were not validated with retrograde tracers. See Table 1 for full list of abbreviations. **b-m.** Two-sided pairwise Wilcoxon rank sum tests were performed on anterograde label density from BLA.am (n=8), BLA.al (n=6), and BLA.ac (n=7). Parameters that survived FDR correction for multiple testing with p-values less than 0.05 are reported and visualized with whisker plots for PL2 (b), ILA (c-d), CA3 (e), DP (f), ACAd (g), MOs (h), CA1 (i), PAR (j), SUBv (k), SUBd (l), and TTd (m). The center line represents the median, the box limits the upper and lower quartiles, and the whiskers the 1.5x interquartile range. * denotes $p<0.05$, ** $p<0.005$, *** $p<0.0005$, and ns=not significant. See *Statistical analysis of BLAa projections* in Methods for full statistical reporting. Source data are provided as a Source Data file. *Abbreviations: PL2: prelimbic cortical area layer 2; ILA: infralimbic cortical area; DP: dorsal peduncular area; ACAd: anterior cingulate cortical area, dorsal part; MOs: secondary motor cortex; PAR: parasubiculum; SUBv: subiculum, ventral part; SUBd: subiculum, dorsal part; TTd: taenia tecta, dorsal part.*

Supplementary Fig. 6

a

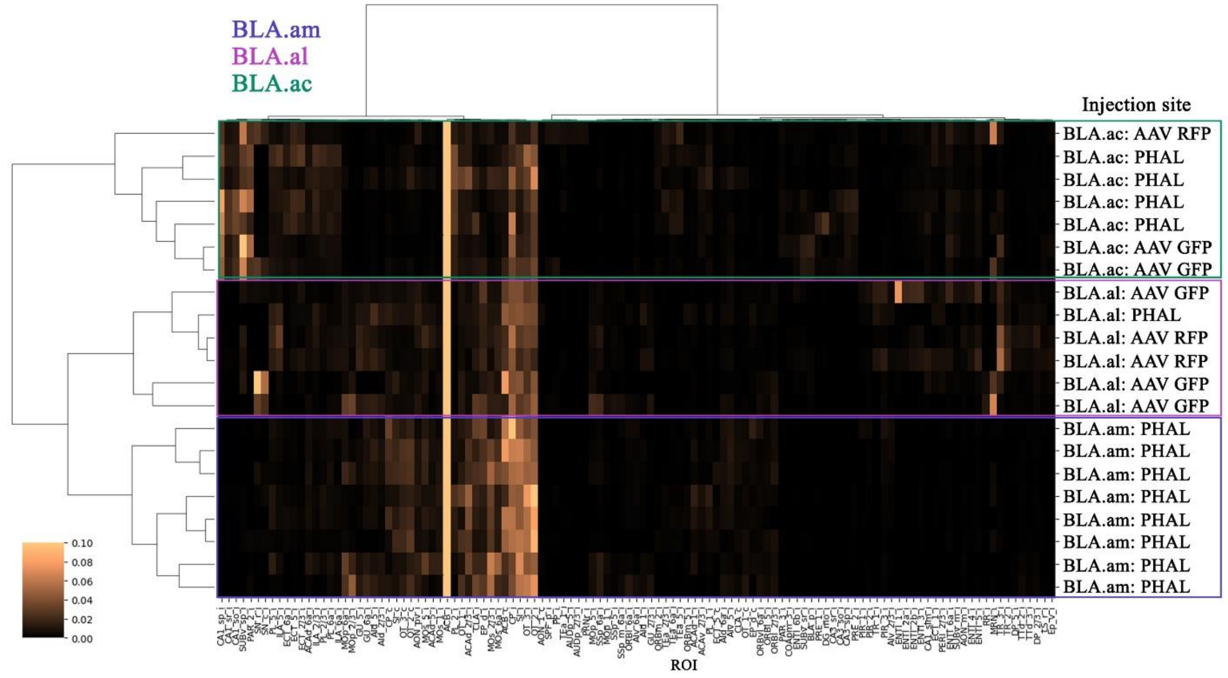

b

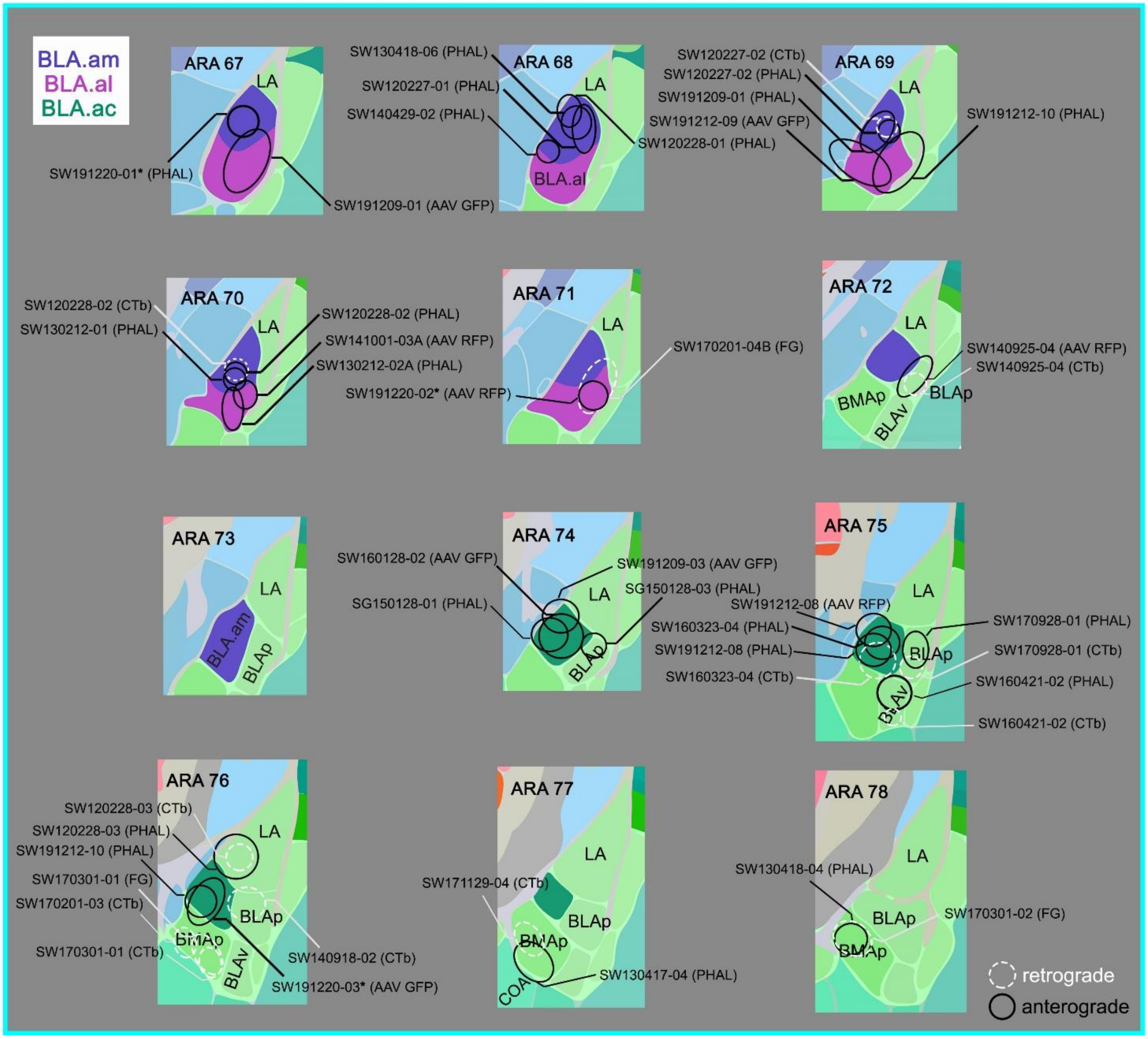

Supplementary Fig. 6: BLA injections

a. Anterograde tracer injections were repeated in each BLAa domain (BLA.am n=8; BLA.al n=9; BLA.ac n=7). A 2D hierarchical clustering algorithm that groups injection sites based on their projection density to brain-wide ROIs was used to assess the validity of injections. The matrix that visualizes the clustering shows clear segregation of injections made in BLA.am from those made in the BLA.al and BLA.ac based on anterograde labels. **b.** Ellipses show approximate locations of BLA anterograde (solid black) and retrograde (dashed white) tracer injection sites in male mice. * denotes injections made in female mice. Note that these denote only centers of the injection sites. Some tracer spread was observed across ARA levels, which are not represented here. In each case however, much of the tracer was confined within the nucleus denoted here. In total, 44 BLA injection cases were used.

Supplementary Fig. 7

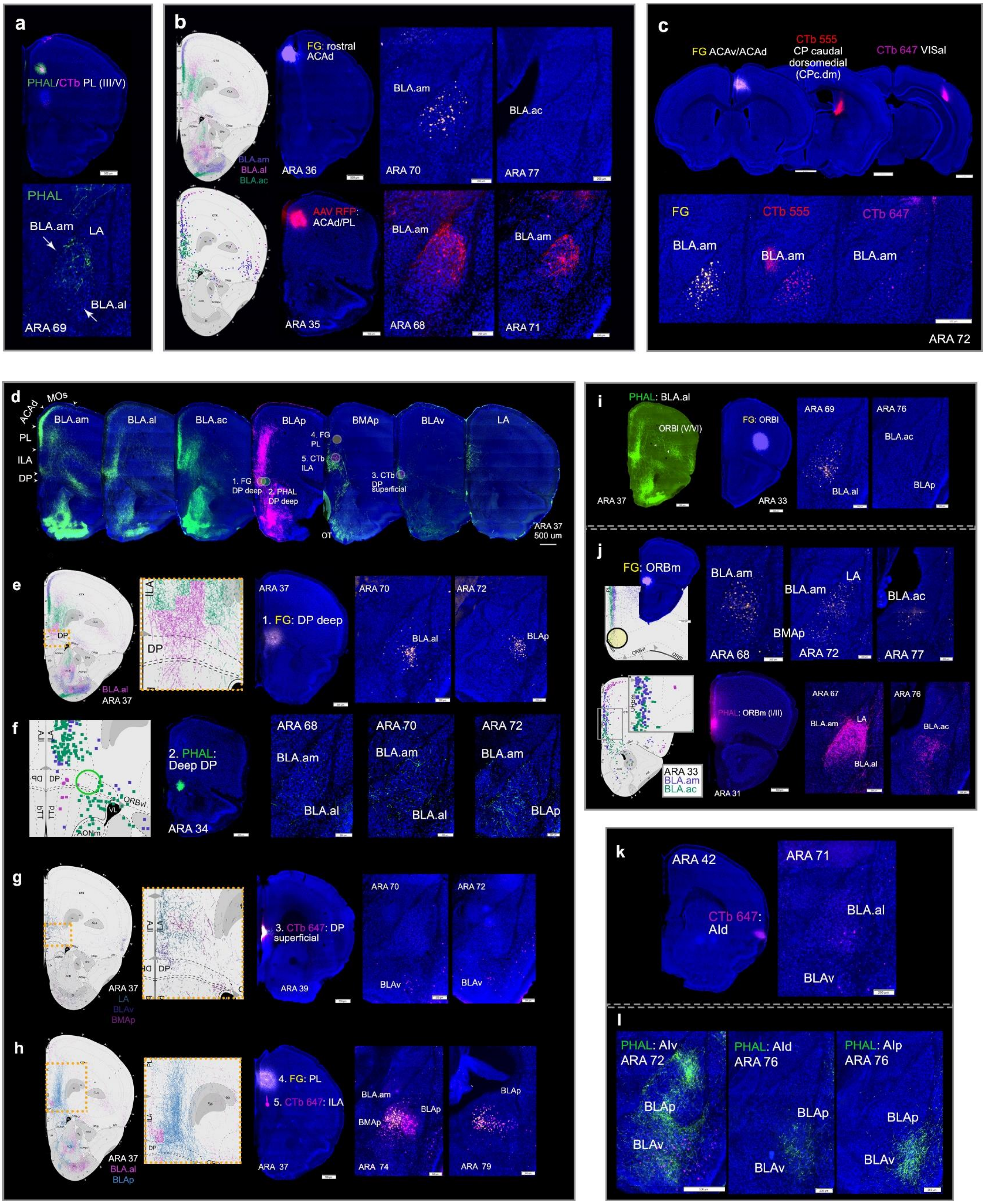

Supplementary Fig. 7: BLA and cortical connections

a. PHAL/CTb injection in deeper layers of PL show sparse label in BLA.am, confirming the sparser PL(V)→BLA.am connection. **b.** Top panels: anterograde map shows strong BLA.am→rostral ACAd projections. A FG injection in rostral ACAd labels projection cells in BLA.am confirming the connection. Bottom panels: retrograde map shows back-labeled neurons in rostral ACAd from BLA.am injection. An AAV RFP injection in rostral ACAd confirms this rostral ACAd→BLA.am connection. **c.** Three retrograde tracers injected in ACA (FG), caudal CP dorsomedial (CTb 555), and in deep layers of VISal (CTb 647) back-label BLA.am projection neurons at ARA 72 validating BLA.am projection to visual processing areas (BLA.am→ACA/CP/VISal). **d.** Projections to MPF areas from injections in BLA.am, BLA.al, BLA.ac, BLAp, BMAp, BLAv, and LA. Numbers denote injections made in different MPF regions in e-h to validate connections. **e.** Anterograde maps show BLA.al neuron projections to deep layers of DP. Insets are magnifications of boxed regions on the maps. FG injection 1 in deep DP back-labels projections neurons in BLA.al and BLAp validating BLA.al/BLAp→DP deep layer connections. **f.** Projections from DP to BLA are weak as shown by the retrograde map. Injection 2, a PHAL injection in deep layers of DP (denoted by ellipse on map), confirms these weak DP deep→BLA connections. **g.** Anterograde maps show BLAv projections to superficial layers of DP. Injection 3, a CTb injection in superficial layers of DP, back-labels BLAv projection neurons validating the connection (BLAv→DP superficial). **h.** Anterograde map showing BLAp projections to deep layers of PL and ILA. Injections 4 (FG in PL) and 5 (CTb in ILA) show FG back-labeled BLAp and BLA.am projection cells, confirming those connections (BLA.am/BLAp→PL), and CTb back-labeled BLAp and BMAp neurons validating BLAp/BMAp→ILA (deeper layer) connections. **i.** Left panel shows BLA.al projections to ORBl (orbital area, lateral part), a connection validated by a FG injection in ORBl that back-labels BLA.al projection neurons (BLA.al→ORBl). **j.** Top panels: anterograde map shows BLA.am and BLA.ac projections to ORBm. A FG injection in ORB, denoted by the ellipse on the map, back-labels projection neurons in BLA.am, BLA.ac, and also BMAp validating BLA.am/BLA.ac/BMAp→ORBm connections. Bottom panels: retrograde map shows ORBm labeled neurons from injections in BLA.am and BLA.ac. PHAL injected into ORBm labels BLA.am, BLA.ac, and LA validating BLA.am/BLA.ac/LA→ORBm projections. **k.** Validation of BLA.al/BLAv→AId connection with CTb injection in AId. **l.** AI projections to BLAp and BLAv. PHAL injections in AIv, AId, and AIp show relatively weak projections to BLAp. *Abbreviations: ACAd: anterior cingulate cortex, dorsal part; ACAv: anterior cingulate cortex, ventral part; AId: agranular insular cortical area, dorsal part; AIp: agranular insular cortical area, posterior part; AIv: agranular insular cortical area, ventral part; CP: caudoputamen; DP: dorsal peduncular area; ORBm: orbital cortical area, medial part; PL: prelimbic cortical area; ILA: infralimbic cortical area; VISal: visual cortical area, anterolateral part.*

Supplementary Fig. 8

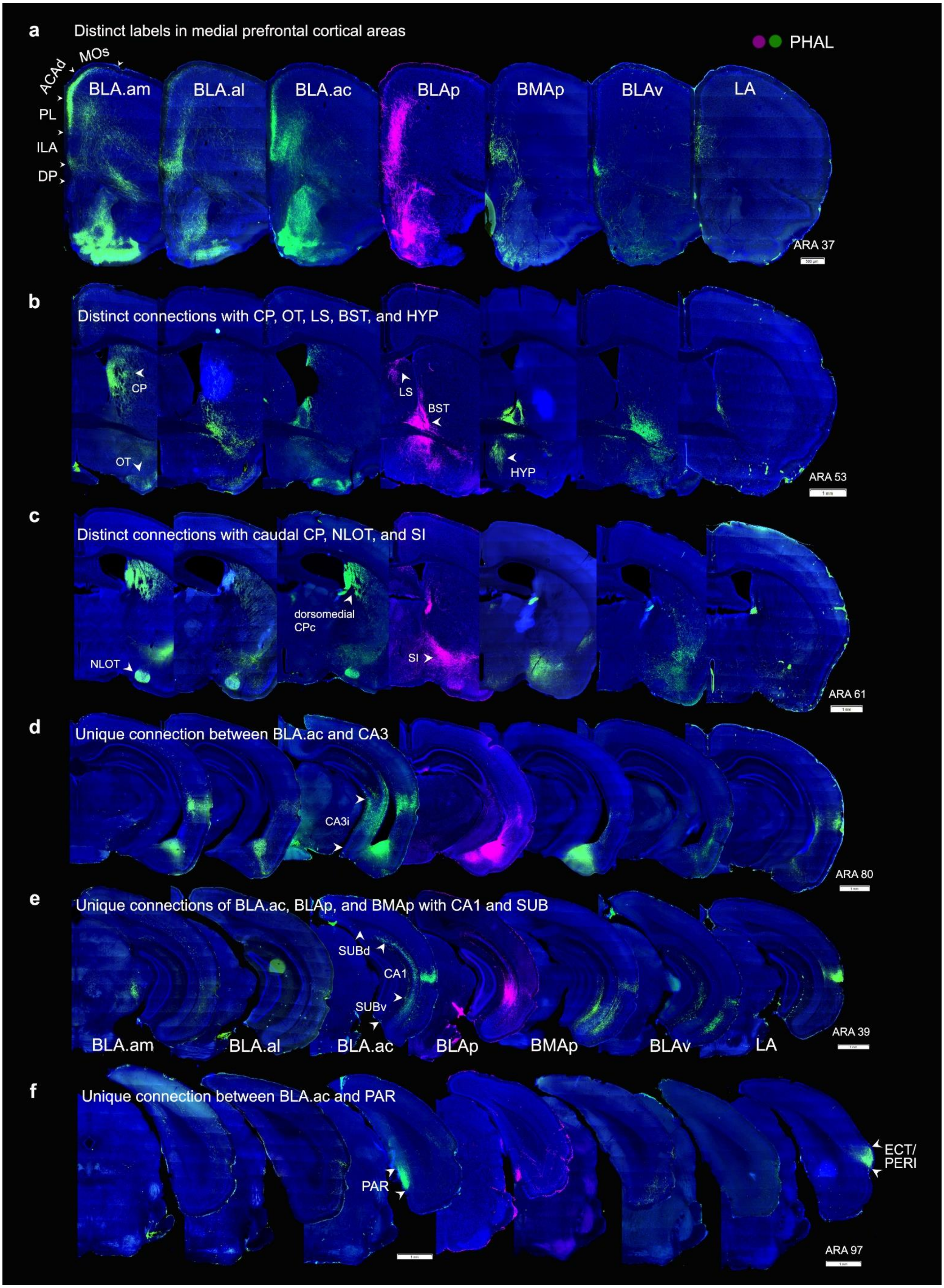

Supplementary Fig. 8: BLA whole brain projections

a. Anterograde injections made in BLA.am, BLA.al, BLA.ac, BLAp, BMAp, BLAv, and LA show distinct labels in medial prefrontal cortical areas (MPF) like anterior cingulate cortex (ACA), prelimbic cortex (PL), infralimbic cortex (ILA), and dorsal peduncular area (DP). BLA.am neurons project to more dorsal MPF like ACA and dorsal PL, while BLA.al neurons target more ventral MPF like ILA and DP. BLA.ac neurons project mostly to ventral PL. BLAp neurons target deeper layers of all MPF structures, while those in BMAp, BLAv, and LA target ILA. **b.** BLA.am, BLA.al, and BLA.ac neurons project to different regions of caudoputamen (CP). BLA.am and BLA.ac project to dorsomedial CP, while BLA.al targets its ventral regions. Note also strong input to ventral CP at ARA 53 from BLAv neurons. BMAp and BLAp also project strongly to bed nucleus of stria terminalis (BST) and hypothalamic (HYP) regions. BLA.ac neurons also show some projections to BST. **c.** BLA.am, BLA.al, and BLA.ac neurons project to NLOT. BLA.am and BLA.ac strongly target dorsomedial CP at more caudal levels. **d.** Shows the unique projection from BLA.ac to CA3. **e.** BLA.ac, BLAp, and BMAp neurons project to hippocampal CA1 and subiculum (SUB), while BLA.am, BLA.al, and BLAv do not. **f.** Shows the distinct projection from BLA.ac neurons to parasubiculum (PAR) and from LA neurons to perirhinal (PERI) and ectorhinal (ECT) cortical areas.

Supplementary Fig. 9

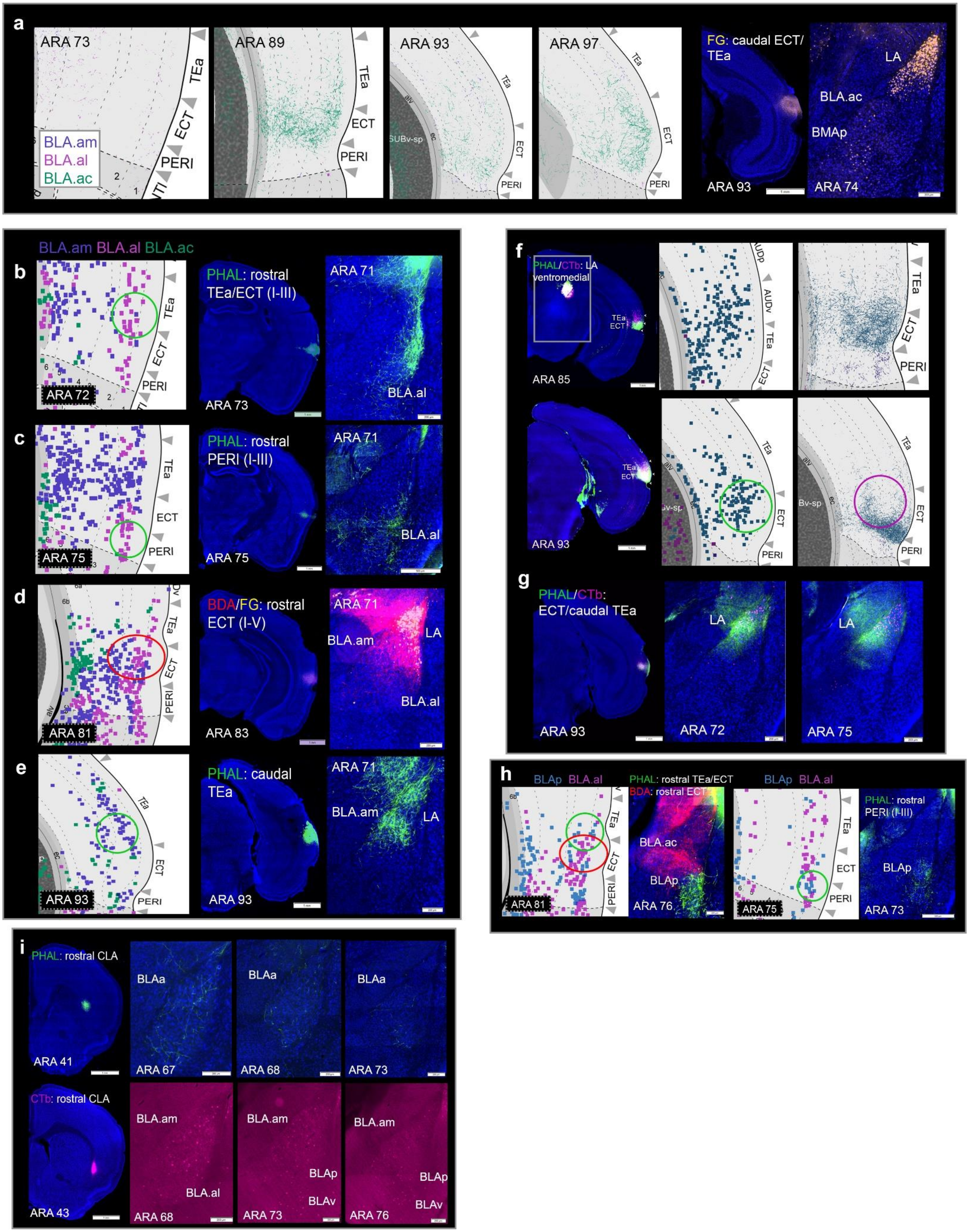

Supplementary Fig. 9: BLA connections with perirhinal cortical structures and claustrum

a. Anterograde maps showing weak projections from BLA.am, BLA.al, and BLA.ac to TEa, ECT, and PERI. Some BLA.ac projections are evident in caudal ECT. A FG injection in caudal ECT validates this weak BLA.ac→ECT caudal projection, but also shows (1) weak projections from BMAp to ECT caudal (BMAp→ECT caudal), and (2) that strongest projections to ECT caudal are from LA neurons. **b-d.** Left: retrograde maps show rostral TEa/ECT (b), rostral PERI (c), and rostral ECT (d) labeled cells following retrograde tracer injections in BLA.al, but also in BLA.am. **e.** Caudal TEa cells are labeled following retrograde injection in BLA.am. Ellipses denote location of anterograde PHAL (green) or AAV-RFP (red) injections to validate rostral TEa/ECT/PERI→BLA.al and caudal TEa→BLA.am connections to the right. d also shows strong reciprocal connections between rostral ECT and LA, while e demonstrates caudal TEa→LA projections. **f.** Raw data and maps showing LA projection neurons heavily innervating ECT and TEa at ARA levels 85 (top) and 93 (bottom). Panels also show ECT and TEa cells projecting back to LA at both ARA levels. **g.** Strong PHAL and CTb labeling is present in TEa and ECT following a co-injection into LA (ventromedial part), validating LA→TEa/ECT and TEa/ECT→LA connections. Ellipses in f denote locations of anterograde PHAL (green) and CTb (pink) injections. **h.** Retrograde maps show labeled cells in rostral TEa, ECT, and PERI following a retrograde tracer injection into BLAp. Ellipses denote locations of anterograde tracer injections PHAL (green) and BDA (red) to validate rostral TEa/ECT/PERI→BLAp projections. **i.** Top panels: PHAL in rostral claustrum (CLA) shows weak CLA→BLAa connections. Bottom panels: CTb retrograde tracer in rostral CLA shows weak BLAa→CLA connections. *Abbreviations: ECT: ectorhinal cortical area; PERI: perirhinal cortical area; TEa: temporal association area*.

Supplementary Fig. 10

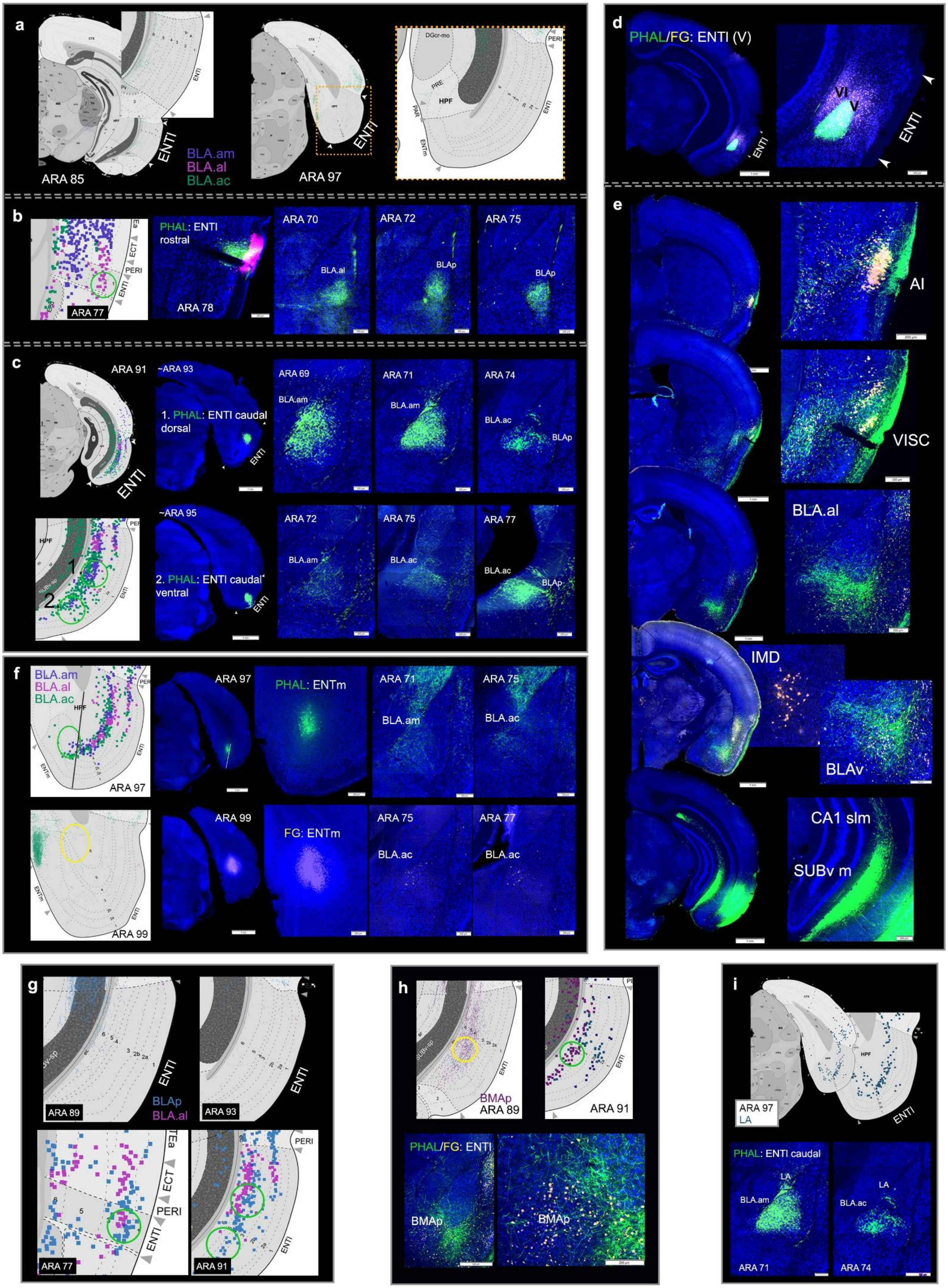

Supplementary Fig. 10: BLA connections with entorhinal cortical areas

a. Anterograde maps of tracer injections in BLA.am, BLA.al, and BLA.ac showing weak projections to ENTl. Inset is magnification of boxed region on the map. **b.** ENTl shows topographic projections to different BLAa domains. Retrograde map on left shows back-labeled rostral ENTl projection neurons from a BLA.al retrograde injection. Ellipse denotes location of PHAL (green) tracer injection that validates the rostral ENTl→BLA.al connection, and shows rostral ENTl→BLAp projections. **c.** Left panels show retrograde map with labeled caudal dorsal and ventral ENTl cells from retrograde injections in BLA.am and BLA.ac. Ellipses denote PHAL injection locations in the dorsal (injection 1) and ventral (injection 2) caudal ENTl that validate caudal dorsal ENTl→BLA.am/BLA.ac and caudal ventral ENTl→BLA.ac connections. In injection 1, note the sparser label in BLA.ac compared to BLA.am. In injection 2, note the strong label in BLA.ac. **d.** Connectivity of a specialized region in ENTl(V) was examined with a PHAL and FG injection. **e.** FG labels in AI and BLAv projection neurons confirm BLAv/AI→ENTl(V) connections. Further, FG injection shows ENTl(V) receives input from VISC and IMD, two regions also involved in gustatory/visceral processing. The PHAL injection shows output of ENTl(V) to AI, VISC, BLA.al, and SUBv. **f.** Top panels show weak projections from ENTlm to BLA.am and BLA.ac. Bottom panels show weak, but observable projections from BLA.ac to ENTm (BLA.ac→ENTm). **g.** Weak projections from BLAp neurons to ENTl shown (top). Bottom panels: retrograde maps with back-labeled rostral (ARA 77) and caudal dorsal and ventral ENTl (ARA 91) neurons from a retrograde BLAp injection. Ellipses denote PHAL (green) injection locations that correspond to injections in b-c and validate rostral ENTl/caudal dorsal ENTl/caudal ventral ENTl→BLAp connections. **h.** Top panels: anterograde and retrograde maps showing BMAp→ENTl(IV-VI) and ENTl(IV-VI)→BMAp connections. Ellipses denote locations of FG and PHAL injections that validate those connections (bottom panels). Note PHAL labeled fibers and FG labeled cells in BMAp following injections. **i.** Caudal ENTl→LA connections validated via a PHAL injection. *Abbreviations: AI: agranular insular cortical area; IMD: intermediodorsal thalamic nucleus; ENTl: entorhinal cortex, lateral part; ENTm: entorhinal cortex, medial part; SUBv: subiculum, ventral part; VISC: visceral cortical area.*

Supplementary Fig. 11

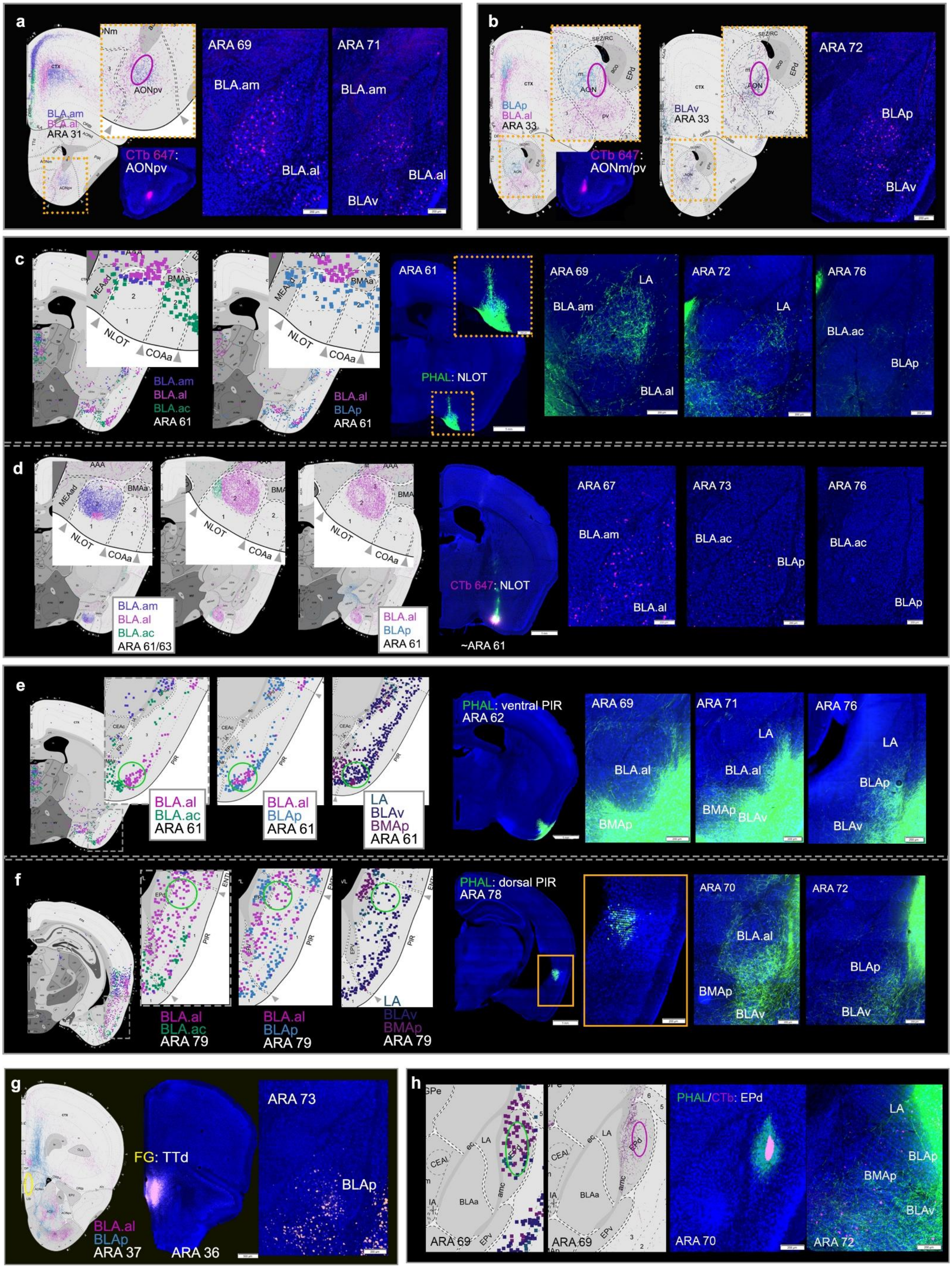

Supplementary Fig. 11: BLA connections with olfactory structures

a. Anterograde maps show BLA.am and BLA.al neuron projections to AONpv (anterior olfactory nucleus, posteroventral part). Inset is magnification of boxed region on map. Ellipse denotes location of CTb 647 injection that validates BLA.am/BLA.al→AONpv connection, but also back-labels BLAv projection neurons (BLAv→AONpv). **b.** Anterograde maps show BLAp and BLAv projections to AONm (anterior olfactory nucleus, medial part). Insets are magnifications of boxed regions on maps. A retrograde CTb injection in AONm validates BLAp/BLAv→AONm connection. **c.** Retrograde maps show labeled NLOT(I/II) (nucleus of lateral olfactory tract) neurons following retrograde tracer BLA.am, BLA.al, and BLAp injections. A PHAL NLOT injection labels BLA.am, BLA.al, and LA validating NLOT→BLA.am/BLA.al/LA connections, but not the NLOT→BLAp connection. Note the absence of labeled fibers in BLAp. Labeled NLOT projection cells may be due to ~5% leakage into LA. Inset is magnification of boxed injection site region. **d.** Anterograde maps show BLA.am and BLA.al projection neurons targeting NLOT. Note the absence of BLAp fibers in NLOT in BLA.al versus BLAp comparison. A CTb injection in NLOT validates the BLA.am/BLA.al→NLOT connections. Note absence of labeled cells in BLAp and BLA.ac. **e.** Retrograde maps show labeled ventral PIR (piriform cortical area) cells following retrograde tracer injections in BLA.al, BLAp, BLAv, BMAp, and LA. Ellipse denotes location of PHAL injection to validate connections. A PHAL injection in ventral PIR confirms the ventral PIR→BLA.al/BLAp/BLAv/BMAp/LA connections. **f.** Retrograde maps show labeled dorsal PIR neurons following retrograde tracer injections in BLA.al and BLAv. Ellipse denotes location of PHAL injection for validation. The dorsal PIR PHAL injection labels fibers primarily in BLA.al, BLAv, but also in BLAp confirming BLA.al/BLAv/BLAp→dorsal PIR projections. Inset shows magnified injection site location. **g.** Anterograde map showing BLAp neuron projections to TTd/TTv (TTd: taenia tecta, dorsal part; TTv: taenia tecta, ventral part). Ellipse denotes location of FG injection that validates the BLAp→TTd connection. **h.** Retrograde and anterograde maps showing EPd→BMAp and BMAp→EPd (dorsal endopiriform nucleus) connections. Ellipses denote PHAL and CTb injection locations to validate those connections. PHAL EPd injection shows labeled fibers in BMAp, but also in BLAp and BLAv (EPd→BMAp/BLAp/BLAv), while CTb labels cells only in BMAp (BMAp→EPd).

Supplementary Fig. 12

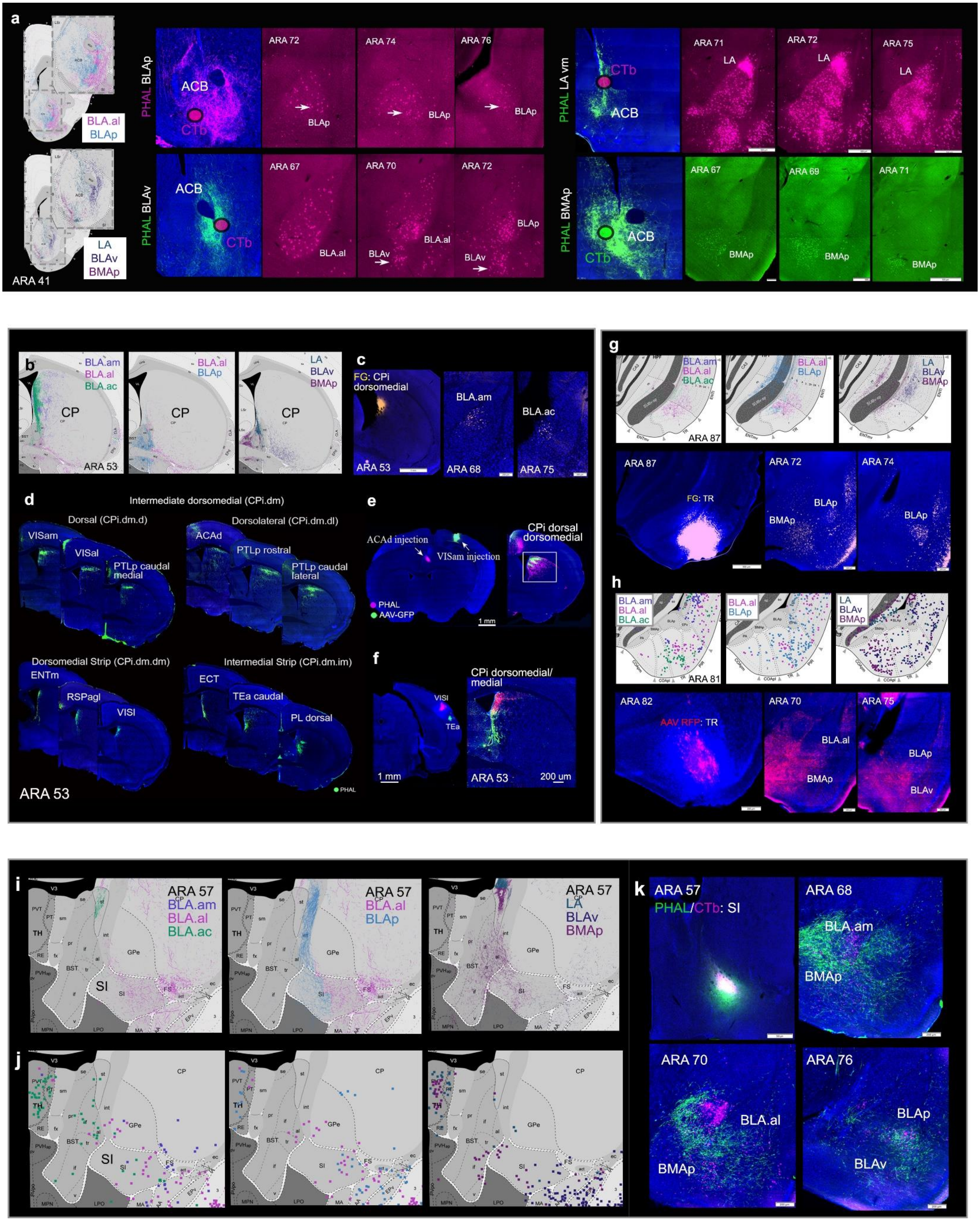

Supplementary Fig. 12: BLA connections with striatum, postpiriform transition area, and substantia innominata

a. Anterograde maps show BLAp and BMAp projections to medial ACB (nucleus accumbens), BLAv to ACB core, and LA to intermediate ACB. Raw images of projections from anterograde PHAL BLA injections to these ACB regions are shown. Ellipses denote location of retrograde tracer injections to validate each BLA→ACB projection. A CTb 647 (pink) injection in medial ACB back-labels BLAp projection neurons (BLAp→medial ACB), CTb 647 (pink) in ACB core labels BLAv neurons (BLAv→ACB core), CTb 647 (pink) in medial ACB back-labels projection neurons in LA (LA→ACB medial), and CTb 488 (green) in medial ACB back-labels BMAp neurons (BMAp→ACB medial). Note: for each case, retrograde validation injections were placed to overlap with labeled ACB fibers from anterograde tracer injections in BLAp, BLAv, LA, and BMAp. **b.** Anterograde maps showing BLA.am and BLA.ac projections to dorsomedial part of CP intermediate (CPi) and BLA.al and BLAv projections to ventral CPi. **c.** A FG retrograde injection in dorsomedial CPi back-labels projection neurons in BLA.am and BLA.ac validating those connections (BLA.am/BLA.ac→CPi dorsomedial). **d.** Shows cortical projections to distinct CPi domains. For example, BLA.am projections to CPi overlap with fibers from the VISam, VISal, PTLp, and ACAd in dorsomedial regions, while projections from BLA.ac overlap with fibers from ENTm, RSPagl, and VISl in CPi dorsal and dorsomedial domains. LA fibers overlap with projections from ECT, TEa caudal, and PL in CPi medial domains [1]. **e.** ACAd and VISam projections to CPi dorsal and dorsomedial regions, where BLA.am fibers also terminate [1]. **f.** Fibers from VISl and TEa terminate in dorsomedial and medial parts of CPi, precisely where BLA.ac and LA fibers terminate [1]. **g.** Anterograde maps showing BLA.al, BLAp, and BMAp projections to TR (postpiriform transition area). Bottom panels show a TR FG injection that validates the BLA.al/BLAp/BMAp→TR connections. **h.** Retrograde maps showing TR back-labeled cells from retrograde injections in BLA.al, BLAp, BLAv, and BMAp. Bottom panels show AAV RFP anterograde injection that validates TR→BLA.al/BLAp/BLAv/BMAp connections. **i.** Top panels: anterograde maps showing BLA.al, BLAp and BMAp neurons targeting SI (substantia innominata). Bottom panels: retrograde maps showing SI labeled cells from retrograde injections in BLA.al, BLAv, BMAp. **k**. An SI CTb injection validates the BLA.al/BLAp/BMAp→SI connections, while the SI PHAL injection validates the SI→BLA.al/BLAv/BMAp connections. *Abbreviations: ACAd: anterior cingulate cortex, dorsal part; ECT: ectorhinal cortical area; ENTm: entorhinal cortical area, medial part; PL: prelimbic cortical area; PTLp: posterior temporal association area; RSPagl: retrosplenial cortical area, agranular part; TEa: temporal association area; VISal: visual cortical area, anterolateral part; VISam: visual cortical area, anteromedial part; VISl: visual cortical area, lateral part.*

Supplementary Fig. 13

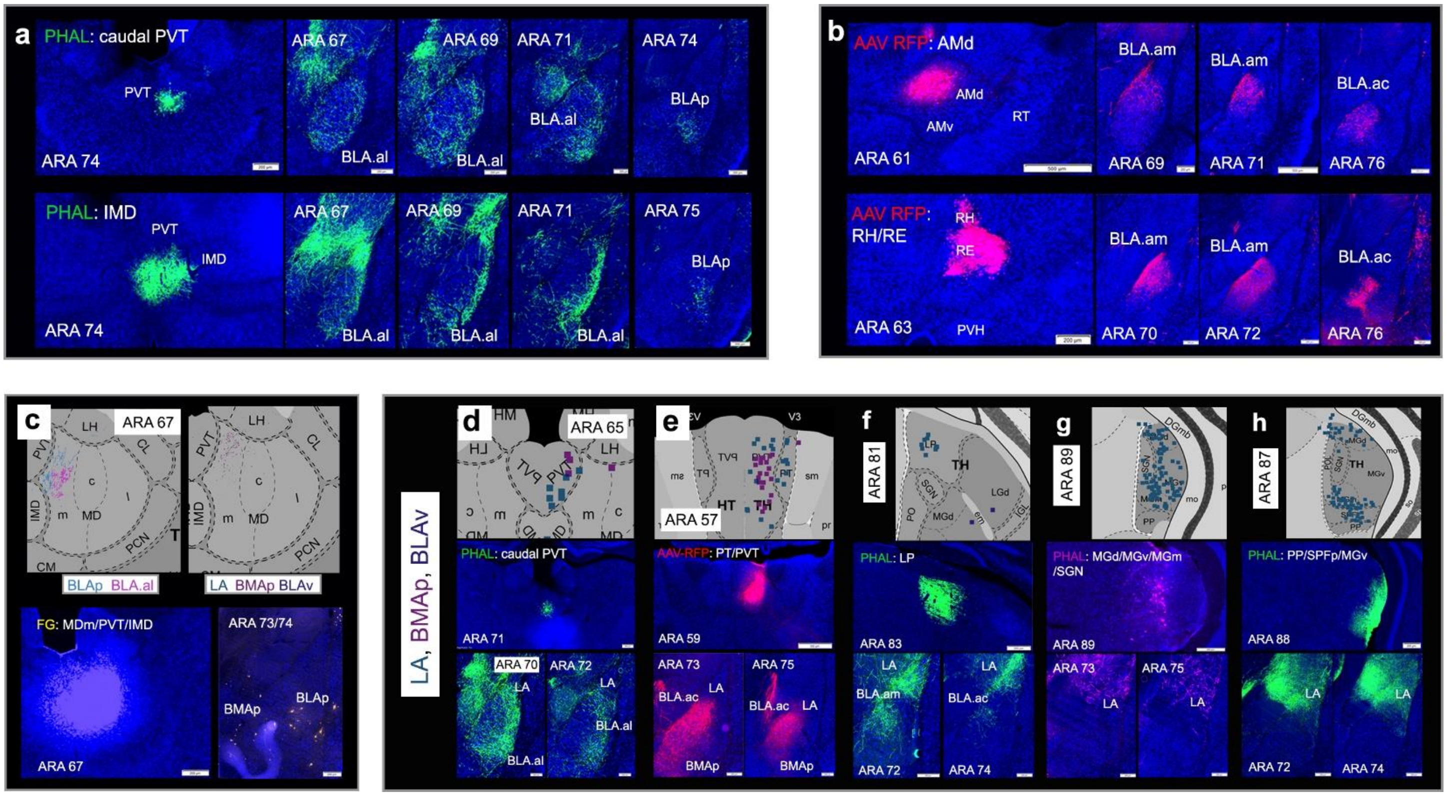

Supplementary Fig. 13: BLA-thalamic connections

a. Anterograde tracer injections in caudal PVT and IMD validate the caudal PVT/IMD→BLA.al connections. Caudal PVT also shows projections to BLAp (caudal PVT→BLAp). **b.** Anterograde tracer injections in AMd and RH/RE validate the AMd→BLA.am/BLA.ac and RH/RE→BLA.am/BLA.ac connections. **c.** Top: anterograde maps showing BLAp and BMAp neuron projections to MDm. Bottom: FG MDm injection validates the MDm→BLAp/BMAp connections. **d.** Top: retrograde map showing caudal PVT cells labeled following a retrograde tracer injection in LA. Bottom: PHAL tracer in caudal PVT validates the caudal PVT→LA connection and also caudal PVT→BLA.am/BLA.al projections. **e.** Top: retrograde map showing rostral PVT and PT cells labeled following retrograde tracer injections in LA and BMAp. Bottom: anterograde AAV-RFP injection validates rostral PVT/PT→LA/BMAp projections, but also rostral PVT/PT→BLA.ac projection. **f.** Top: retrograde map showing LP labeled cells following retrograde tracer injection in the LA. Bottom: PHAL injection in LP validates the LP→LA connection, but also shows LP→BLA.am connection. **g.** Top: retrograde map showing MGd/MGv labeled cells following retrograde tracer injection in LA. Bottom: PHAL injection in MGd/MGv validates MGd/MGv→LA projections. **h.** Top: retrograde map showing PP, SPFp, MGv labeled cells following retrograde tracer injection in the LA. Bottom: PHAL injection in PP/SPFp/MGv validates the PP/SPFp/MGv→LA connection. *Abbreviations: AMd: anteromedial nucleus of the thalamus, dorsal part; LP: lateral posterior thalamic nucleus; LP: lateral posterior thalamic nucleus; MDm: mediodorsal thalamic nucleus, medial part; MGd: medial geniculate complex, dorsal part; MGm: medial geniculate complex, medial part; MGv: medial geniculate complex, ventral part; RH: rhomboid nucleus; RE: reunions nucleus; RT: reticular nucleus of the thalamus; SPFp: subfascicular nucleus, parvocellular part.*

Supplementary Fig. 14

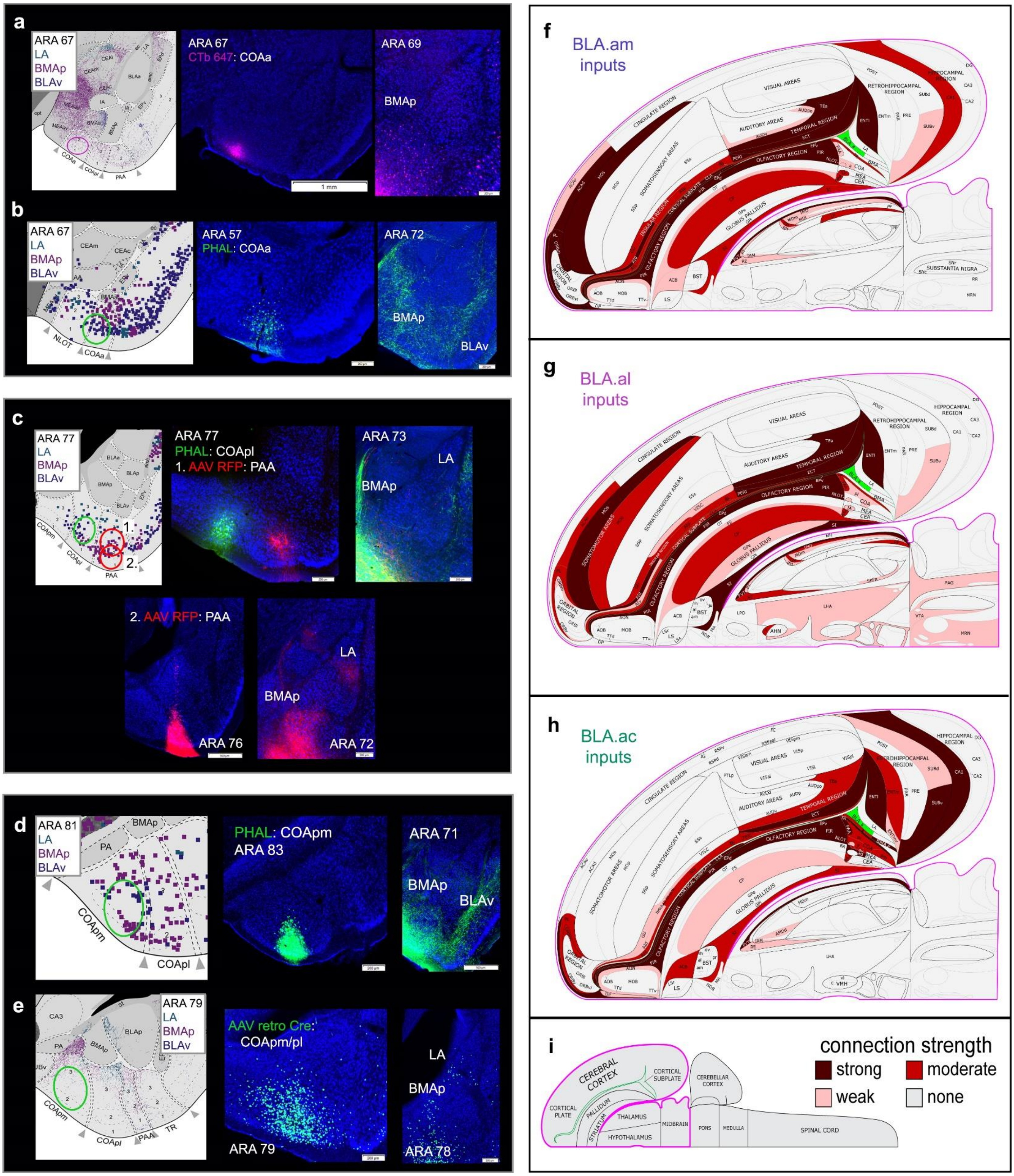

Supplementary Fig. 14: BLA connections with cortical amygdalar areas and flatmaps of BLAa retrograde tracing

a. Left: anterograde map showing BMAp neuron projections to COAa (cortical amygdala area, anterior part), a connection validated with a CTb injection in COAa that back-labels BMAp neurons (BMAp→COAa). Ellipse on map denotes location of COAa CTb injection site. **b.** Retrograde map shows COAa labeled cells from retrograde tracer injections in BMAp and BLAv. COAa→BMAp/BLAv connections are validated with a PHAL injection in COAa, which labels fibers in BMAp and BLAv. Ellipse on map denotes location of PHAL injection. **c.** Top: retrograde map shows back-labeled COApl (cortical amygdala area, posterolateral part) and PAA (piriform-amygdala area) cells following retrograde tracer injections in BMAp and LA. These connections are validated with anterograde PHAL (green ellipse) and AAV-RFP (1. red ellipse) injections in COApl and PAA, which label fibers in BMAp and LA (COApl/PAA→BMAp/LA). Bottom: An AAV-RFP injection in PAA (2. red ellipse) more clearly labels BMAp and LA as further validation of those connections. **d.** Retrograde map showing COApm (cortical amygdala area, posteromedial part) labeled cells following retrograde tracer injections in BMAp and BLAv. A PHAL injection (green ellipse) in COApm labels BMAp and BLAv validating those connections. **e.** Anterograde map shows BMAp projections to COApm. An AAV retro Cre injection (green ellipse) in COApm back-labels projection cells in BMAp validating BMAp→COApm connection. **f-i**. Inputs of BLA.am (**f**), BLA.al (**g**), and BLA.ac (**h**) domains are represented at the macroscale (gray matter region resolution) on a flatmap representation of the mouse brain. The strength values of detected connections were binned into tertiles, and these are represented qualitatively as strong (maroon), moderate (red), weak (pink), and none (gray). For list of abbreviations, see Table 1. **i.** Longitudinal half of the entire CNS flatmap showing orientation and major brain divisions (e.g., cerebral cortex, pallidum, thalamus). The key to the color codes for connection strength is also shown.

Supplementary Fig. 15

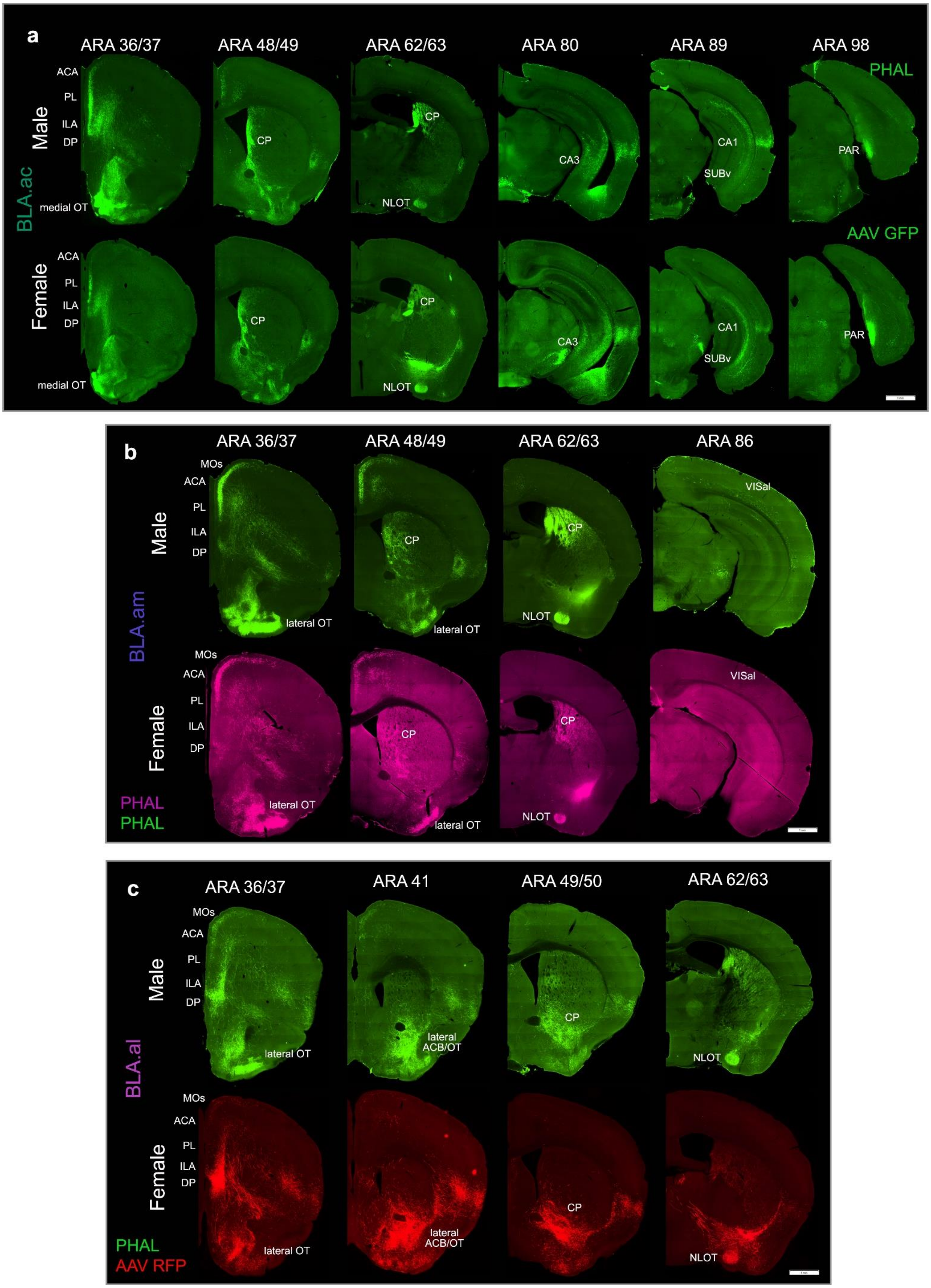

Supplementary Fig. 15: BLAa anterograde connections in male and female mice

a. Shows anterograde labeling across different ARA levels from anterograde tracer injections made primarily in the BLA.ac of male (PHAL: green) or female (AAV GFP: green) mice. Note the consistency of label patterns in medial prefrontal cortical areas like anterior cingulate cortex (ACA), prelimbic cortex (PL), infralimbic cortex (ILA), and dorsal peduncular area (DP), in medial olfactory tubercle (OT), caudoputamen (CP), nucleus of lateral olfactory tract (NLOT), and in hippocampal regions like CA3, CA1, subiculum (SUB), and parasubiculum (PAR). **b.** Shows anterograde labeling across different ARA levels from tracer injections made primarily in the BLA.am of male (PHAL: green) or female (PHAL: pink) mice. Note the consistency of label patterns in supplemental motor cortex (MOs), ACA, PL, ILA, DP, lateral OT, CP, NLOT, and the light labels across deep cortical layers including VISam. **c.** Shows anterograde labeling across different ARA levels from tracer injections made primarily in the BLA.al in male (PHAL: green) or female (AAV RFP: red) mice. Note the consistency of labels patterns in MOs, ACA, PL, ILA, DP, lateral accumbens (ACB), lateral OT, CP, and NLOT.

Supplementary Fig. 16

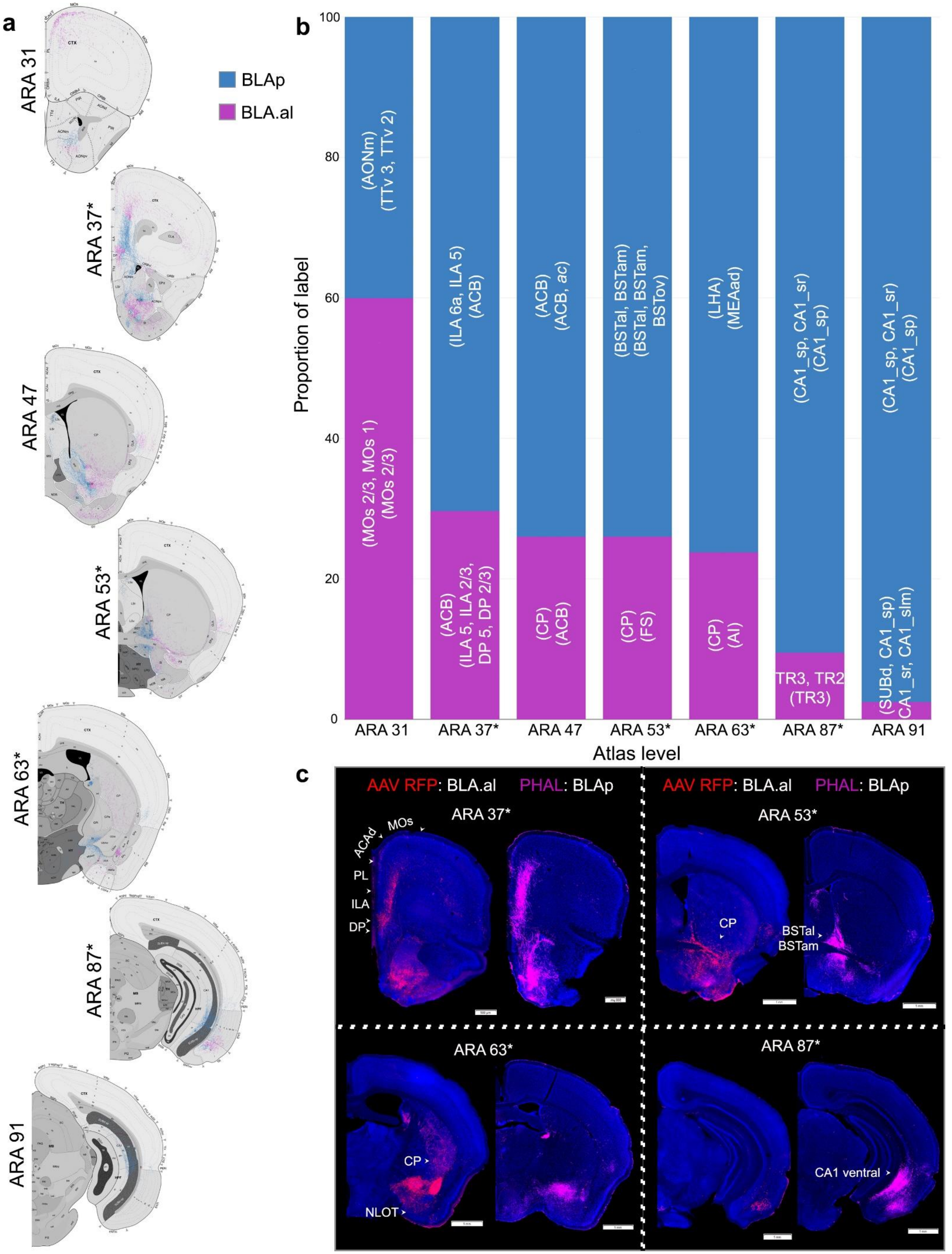

Supplementary Fig. 16: Distinct connections of BLA.al and BLAp

a. Anterograde map from ARA 31-91 showing distinct labeling from BLA.al and BLAp projection neurons. The complete BLA.al and BLAp anterograde map is available at https://mouseconnectomeproject.github.io/amygdalar/. **b.** Bar chart showing the proportion of anterograde labeling across ARA levels 31-91 resulting from representative injections in BLA.al and BLAp (n=1 each). ROIs for grids with strongest label from each injection are included for each ARA section. Note that a grid can include more than one ROI for example, (BSTal, BSTam). Also note how bar graph shows the different targets of BLA.al and BLAp neurons. For example, at level 53, the primary target of BLAp projection cells are the BST, while primary targets for BLA.al neurons are the CP and FS. At level 87, a primary target for BLAp neurons includes the CA1, while for BLA.al the main target at that level is the TR. Raw data from atlas levels with an * are shown in c. **c.** Raw data from atlas levels with an * in b are shown (ARA 37, 53, 63, and 87). Note the difference in ILA and PL labeling at ARA 37. At ARA 53, BLAp neurons target BST, while those in BLA.al target CP, at ARA 63 BLA.al neurons target the CP caudal ventral while there is an absence of label in CP from the BLAp injection. Finally, at level 87 BLAp neurons target CA1, while there is a paucity of label from the BLA.al injection to hippocampal regions. *Abbreviations: BSTam: bed nucleus of stria terminalis, anteromedial part; BSTal: bed nucleus of stria terminalis, anterolateral part; CP: caudoputamen; FS: fundus of striatum; TR: postpiriform transition area.*

Supplementary References

1 Hintiryan, H. *et al.* The mouse cortico-striatal projectome. *Nature neuroscience* **19**, 1100-1114, doi:10.1038/nn.4332 (2016).
