# Supplementary material for: Connectivity characterization of the mouse basolateral amygdalar complex
Source: Nat Commun. 2021 May 17;12:2859. doi: 10.1038/s41467-021-22915-5 (PMC8129205; doi:10.1038/s41467-021-22915-5)
Supplement: Supplementary file 2 — Description of Additional Supplementary Files [file 41467_2021_22915_MOESM2_ESM.docx]

**Description of Additional Supplementary Files**

File Name: Supplementary Movie 1.

Description: BLA.am projection neurons labeled for morphological assessment using a G-deleted rabies (RVΔG) injection made in the dorsomedial part of the caudal caudoputamen (CPc.dm).

File Name: Supplementary Movie 2.

Description: BLA.al projection neurons labeled for morphological assessment using a G-deleted rabies (RVΔG) injection made in the ventral part of the caudal caudoputamen (CPc.v).

File Name: Supplementary Movie 3.

Description: BLA.ac projection neurons labeled for morphological assessment using a G-deleted rabies (RVΔG) injection made in the medial part of the nucleus accumbens (ACB).

File Name: Supplementary Movie 4.

Description: 3D rendering of three reconstructed BLA.ac projection neurons that target the contralateral medial nucleus accumbens (ACB).

File Name: Supplementary Movie 5.

Description: 3D rendering of a single reconstructed BLA.ac projection neuron that targets the contralateral medial nucleus accumbens (ACB).
