# Supplementary material for: Connectivity characterization of the mouse basolateral amygdalar complex
Source: Nat Commun. 2021 May 17;12:2859. doi: 10.1038/s41467-021-22915-5 (PMC8129205; doi:10.1038/s41467-021-22915-5)
Supplement: Supplementary file 8 — Reporting Summary [file 41467_2021_22915_MOESM8_ESM.pdf]

nature research

Corresponding author(s): Hong-Wei Dong
Houri Hintiryan

Last updated by author(s): 2/17/2021

Reporting Summary

Nature Research wishes to improve the reproducibility of the work that we publish. This form provides structure for consistency and transparency in reporting. For further information on Nature Research policies, see Authors & Referees and the Editorial Policy Checklist .

Statistics

For all statistical analyses, confirm that the following items are present in the figure legend, table legend, main text, or Methods section.

n/a	Confirmed	
☐	☒	The exact sample size (*n*) for each experimental group/condition, given as a discrete number and unit of measurement
☐	☒	A statement on whether measurements were taken from distinct samples or whether the same sample was measured repeatedly
☐	☒	The statistical test(s) used AND whether they are one- or two-sided<br>*Only common tests should be described solely by name; describe more complex techniques in the Methods section.*
☒	☐	A description of all covariates tested
☐	☒	A description of any assumptions or corrections, such as tests of normality and adjustment for multiple comparisons
☐	☒	A full description of the statistical parameters including central tendency (e.g. means) or other basic estimates (e.g. regression coefficient) AND variation (e.g. standard deviation) or associated estimates of uncertainty (e.g. confidence intervals)
☐	☒	For null hypothesis testing, the test statistic (e.g. *F*, *t*, *r*) with confidence intervals, effect sizes, degrees of freedom and *P* value noted<br>*Give P values as exact values whenever suitable.*
☒	☐	For Bayesian analysis, information on the choice of priors and Markov chain Monte Carlo settings
☒	☐	For hierarchical and complex designs, identification of the appropriate level for tests and full reporting of outcomes
☒	☐	Estimates of effect sizes (e.g. Cohen's *d*, Pearson's *r*), indicating how they were calculated

Our web collection on statistics for biologists contains articles on many of the points above.

Software and code

Policy information about availability of computer code

Data collection	No software was used to collect the data.
Data analysis	An in-house software was used to process all image data presented in the manuscript. Connection Lens was specifically applied to register (warp), threshold (segment), and annotate the labels in all image data. This software has not been publicly released yet.<br><br>However, custom code developed for data analysis is available at https://github.com/ibowman/BLA. For the Louvain algorithm implementation, the Brain Connectivity Toolbox (BCT) was employed, which freely available at https://sites.google.com/site/bctnet/. The grid communities algorithm for matrix visualizations are available here at https://github.com/aestrivex/bctpy, https://sites.google.com/site/bctnet/. Geometric processing of neuron models was performed using the Quantitative Imaging Toolkit (QIT), available at http://cabeen.io/qitwiki. We also computed persistence diagrams using NeuronTools (https://github.com/Nevermore520/NeuronTools) and then computed inter-neuron distances using the Wasserstein metric (https://bitbucket.org/grey_narn/geom_matching/src). We used scikit-learn for SVM implementation, and scipy and seaborn to generate and visualize 2D hierarchical clustering results. Finally, a dynamic image carousel web application which features brain-wide pathway reconstructions of tracer-labeled connections from the amygdala was developed. Users can navigate colorful projections of both anterograde and retrograde labels mapped atop the standard ARA atlas. This application is currently hosted at GitHub web portal (https://mouseconnectomeproject.github.io/amygdalar/)<br>and the code repository can be found at https://github.com/mouseconnectomeproject/amygdalar.

For manuscripts utilizing custom algorithms or software that are central to the research but not yet described in published literature, software must be made available to editors/reviewers. We strongly encourage code deposition in a community repository (e.g. GitHub). See the Nature Research guidelines for submitting code & software for further information.

Data

Policy information about availability of data

All manuscripts must include a data availability statement. This statement should provide the following information, where applicable:

- Accession codes, unique identifiers, or web links for publicly available datasets
- A list of figures that have associated raw data
- A description of any restrictions on data availability

Raw data for amygdala cases used in the paper can be accessed through our Mouse Connectome Project website (https://mouseconnectomeproject.github.io/amygdalar/iconnectome). The color-coded visualized output of the community assignments for all injections are available (https://mouseconnectomeproject.github.io/amygdalar/), as well as the global wiring diagram (https://mouseconnectomeproject.github.io/amygdalar/wiringdiagram). Reconstructed BLAa neurons are cataloged in NeuroMorpho.org (neuromorpho.org/dableFiles/dong/Supplementary/Dong2020BLA.zip). Source data also are provided with this paper.

Field-specific reporting

Please select the one below that is the best fit for your research. If you are not sure, read the appropriate sections before making your selection.

☒ Life sciences ☐ Behavioural & social sciences ☐ Ecological, evolutionary & environmental sciences

For a reference copy of the document with all sections, see nature.com/documents/nr-reporting-summary-flat.pdf

Life sciences study design

All studies must disclose on these points even when the disclosure is negative.

Sample size	For connectivity data, 1 representative animal was used for each BLA nucleus for community detection analyses (as is typical for neuroanatomical investigations). As stated in the paper however, these injections were replicated in different animals and all reported connections were validated using different methods.<br><br>Some of the qualitative analysis was validated quantitatively. Output from BLA.am, BLA.al, and BLA.ac was statistically analyzed. Repeated tracer injections were made in the BLA.am (n=8), BLA.al (n=6), and BLA.ac (n=7).<br><br>For morphological analysis of BLAa neurons, 8 neurons were traced for BLA.am, 9 for BLA.al, and 6 for BLA.ac. These sample sizes were sufficient to reach statistical significance for many comparisons suggesting sufficient sample sizes.
Data exclusions	For morphological analyses, not all labeled neurons within a BLAa domain were traced. Many were excluded from the analysis due to our selectivity. First, to ensure the traced neurons in fact solely originated from their respective domains, for all cases, labeled cells visually grouped in the center of the boundaries of each domain (X-Y axes) were traced. Neurons located within the BLA.am were identified for inclusion in the analysis, but those close to the BLA.al or BLA.ac border were excluded. Similarly, neurons within BLA.ac were identified for inclusion, but those close to the border of the BLAp were omitted. In addition, domain-specific neurons in deeper parts of the tissue (z axis) farthest away from the edges of the sectioned tissue were selected for reconstruction. To assist in the accurate selection of neurons, maximum intensity projections of 25 µm were created that aided in the identification of the BLAa domain boundaries. In total, eight neurons were traced for the BLA.am, nine for the BLA.al, and six for the BLA.ac.<br><br>Further, due to the differences in slice thickness (250 µm for BLA.am and BLA.al and 400 µm for BLA.ac), we restricted our morphological analysis to neurites that were sufficiently close to the soma. We accomplished this by applying the NeuronTransform module in QIT to trim the contiguous portion of neurites that measured farther than 300 nm away from the center of the soma using the Euclidean distance.<br><br>All of this information is included in the Online Methods.
Replication	For connectivity data, data from injections in all different BLA nuclei were replicated. Further, reported connections were validated via different methods.
Randomization	Not relevant to the present studies since animals or neurons were not assigned to different experimental groups/conditions.
Blinding	For neuron tracing, blinding was not possible since significant effort was dedicated to ensure that only labeled neurons within the specific BLAa domains were traced.

Reporting for specific materials, systems and methods

We require information from authors about some types of materials, experimental systems and methods used in many studies. Here, indicate whether each material, system or method listed is relevant to your study. If you are not sure if a list item applies to your research, read the appropriate section before selecting a response.

Materials & experimental systems

n/a	Involved in the study	
☐	☒	Antibodies
☒	☐	Eukaryotic cell lines
☒	☐	Palaeontology
☐	☒	Animals and other organisms
☒	☐	Human research participants
☒	☐	Clinical data

Methods

n/a	Involved in the study	
☒	☐	ChIP-seq
☒	☐	Flow cytometry
☒	☐	MRI-based neuroimaging

Antibodies

Antibodies used

Primary antibodies
rabbit anti-Phal antibody (Vector Laboratories, #AS-2300)
mouse anti-Cre recombinase antibody (EMD Millipore, #MAB3120)
Secondary antibodies
anti-rabbit IgG conjugated with Alexa Fluor® 488 or 647 (Invitrogen, 488: #A-21206; 647: #A-31573) for Phal
anti-mouse IgG conjugated with Alexa Fluor® 488 or 647 (Life Technology, 488: #A-21202; 647: #A-31571) for Cre recombinase

Nissl stain
NeuroTrace® 435/455 (NT; 1:500; Invitrogen, #N21479)

Validation

All antibody staining has been validated in previous publications (both cited in the manuscript):
Hintiryan et al., The mouse cortico-striatal projectome. (2016), Nature Neuroscience, 19, 1100-1114. https://www.nature.com/articles/nn.4332

Bienkowski et al., Integration of gene expression and brain-wide connectivity reveals the multiscale organization of mouse hippocampal networks. (2018), Nature Neuroscience, 21, 1628-1643. https://www.nature.com/articles/s41593-018-0241-y

Animals and other organisms

Policy information about studies involving animals; ARRIVE guidelines recommended for reporting animal research

Laboratory animals

8-week-old C57BL/6J male and female mice

Wild animals

Study did not involve wild animals.

Field-collected samples

Study did not involve field-collected samples.

Ethics oversight

All experimental procedures were in animal protocols approved by the Institutional Animal Care and Use Committee at USC. A similar statement is included in the current version of the manuscript.

Note that full information on the approval of the study protocol must also be provided in the manuscript.
